# Supplementary material for: Routine antibiotics for infants less than 6 months of age with growth failure/faltering: a systematic review
Source: BMJ Open. 2023 May 10;13(5):e071393. doi: 10.1136/bmjopen-2022-071393 (PMC10174012; doi:10.1136/bmjopen-2022-071393)
Supplement: Supplementary data [file bmjopen-2022-071393supp002.pdf]

Antibiotic as an adjunct to treatment of severe acute malnutrition in infants less than six months of age. A protocol for the systematic review

List of Exclusions and Reasons for Exclusions.

Abate 2020[1]	Inappropriate study design and appropriate control was not available
Adam 1954 [2]	Inappropriate Study Design
Adem 2020[3]	Inappropriate Patient Population. Study was a cohort study and only included children 6-59 months of age
Ahmed 1999[4]	Inappropriate Patient Population. Even though all the children were severely malnourished, all of them had diarrhea. Also, the study was not a randomized study.
Ahmed 2001 [5]	Inappropriate Study Design. Authors discuss a protocol for treatment of severe acute malnutrition. The study was a descriptive review
Akbani 1977[6]	Inappropriate study population. Children with SAM who were infected with tuberculosis.
AmorelliGonzaga 1989[7]	Inappropriate study population. Study investigated miomycin and included both well-nourished and malnourished children.
Amza 2013[8]	Inappropriate study population. Study investigated mass azithromycin distribution and included children irrespective of the nutritional status.
Amza 2014[9]	Inappropriate study population. Study investigated mass azithromycin distribution and included children irrespective of the nutritional status.
Angelakis 2014[10]	Inappropriate study population. Study included patients with Q fever endocarditis patients treated with doxycycline and hydroxychloroquine
Ashorn 2018[11]	Inappropriate study population. Study included children with diarrhea, dysentery and malnutrition.
Ashraf 2012[12]	Inappropriate Comparator. Children aged 2-59 months having severe pneumonia with SAM were randomized to day-care or hospital-care. All the children received antibiotics.
Autret 1989[13]	Inappropriate study Design. Study to assess the pharmacokinetics of gentamicin.
Ayelign 2018[14]	Inappropriate study population. Children with urinary tract infections.
Ayiyi 1987[15]	Inappropriate study Design. The study was a systematic review
Aziz 2015[16]	Inappropriate Intervention. Study investigated vitamin K supplementation.
Berkley 2009 [17]	Wrong comparator
Berkley 2016[18]	Inappropriate Comparator. Both the intervention and comparison group were treated according to the WHO

Antibiotic as an adjunct to treatment of severe acute malnutrition in infants less than six months of age. A protocol for the systematic review

List of Exclusions and Reasons for Exclusions.

	protocol and then randomized to Co-trimoxazole vs. placebo.
Berkowitz 1992[19]	Inappropriate Study Design. Review article.
Bhatnagar 1992[20]	Inappropriate patient population. All the patients had diarrhea. All of them were given gentamicin
Bhatnagar 1996[21]	Inappropriate Study Design. Observation study in children with persistent diarrhea
Birindwa 2020[22]	Inappropriate Study Design. Observational study in children with severe pneumonia
Bleakly 2014[23]	Inappropriate Study Design. Before and after study to assess the effectiveness of a protocol to treat SAM.
Boesen 1953[24]	Inappropriate Indication. Antibiotics were given for treatment of gastroesophageal reflux.
Bolme 1980[25]	Inappropriate Study Design: Pharmacokinetic study for Penicillin in children
Bolme 1988[26]	Inappropriate Study Design: Pharmacokinetic study for streptomycin in children with Tuberculosis.
Bolme 1995[27]	Inappropriate Study Design. Pharmacokinetic study in malnourished children for Penicillin.
Bravo 1982[28]	Inappropriate study design: Study to assess the pharmacokinetics
Bravo 1984[29]	Inappropriate study design: Study to assess the pharmacokinetics
Bredow 1994[30]	Inappropriate Study Design. Observational study
Brodwall 2016[31]	Inappropriate Study Design. Letter to editor
Bruhn 2016[32]	Inappropriate Study Design. Observational study
Brunozi 2019[33]	Inappropriate Study Design. Observational study
Buchanan 1977[34]	Inappropriate Study Design. Letter to editor
Buchanan 1978[35]	Inappropriate Study Design. Observational study
Buchanan 1979 [36]	Inappropriate Study Design. Observational study
Bunn 2009[37]	Inappropriate Study Design. Commentary
Cakir 2012[38]	Inappropriate Patient Population. Children studied leukemia
Caksen 2000[39]	Inappropriate Study Design. Observational study
Caksen 2000[40]	Inappropriate Study Design. Observational study
Cedrato 1974[41]	Inappropriate Study Design. Observational study
Chisti 2015[42]	Inappropriate Patient Population. Children with respiratory symptoms only
Church 2015[43]	Inappropriate Study Design. Observational study
Cochlovius 1953[44]	Inappropriate intervention. Observational study
Dicko 2016[45]	Inappropriate study population. Intervention was given to all the children irrespective of nutritional status at the time of chemoprophylaxis for malaria.
Dieng 2014[46]	Inappropriate Study Design. Observational study
Dubray 2008[47]	Inappropriate study population. Both the study groups received antibiotics and these were given to children 6-59 months of age
Duffau 1990[48]	Inappropriate study population. Children with diarrhea

Antibiotic as an adjunct to treatment of severe acute malnutrition in infants less than six months of age. A protocol for the systematic review

List of Exclusions and Reasons for Exclusions.

Dutta 2006[49]	Inappropriate study design: Observational study
Eriksson 1983[50]	Inappropriate Patient Population. Also, observational study
Eriksson 1988[51]	Inappropriate Patient Population. Also, observational study
Ford 1976[52]	Inappropriate Study Design. Observational study
Friedrich 2016[53]	Inappropriate Study Design. Commentary
Garcia 1974[54]	Inappropriate Study Design. Observational study
Gardner 2011[55]	Inappropriate Study Design. Commentary
Gennaro 2017[56]	Inappropriate Study Design. Commentary
Gensch 1957[57]	Inappropriate Study Design. Observational study
Gernaat 1998[58]	Inappropriate Study Design. Observational study
Ghosh 1995[59]	Inappropriate Patient Population. Children with persistent diarrhea
Girma 2018[60]	Inappropriate Study Design. Observational study
Gore Langton 2020[61]	Inappropriate Patient Population. The antibiotics were given irrespective of nutritional status and study included children beyond six months of age. The comparison group was not appropriate.
Hecht 2015 [62]	Inappropriate Patient Population. The study included children beyond six months of age up to 18 years.
Heikens 1993 [63]	Inappropriate Patient Population, mean age of children at 1.2years.
Heikens 1993 [64]	Inappropriate Comparator, antibiotics compared with receiving health care from community healthcare aids
Hirschhorn 1971 [65]	Inappropriate Study Design, paper is a commentary on the current state of research.
Howard 1967 [66]	Inappropriate Study Design, all infants studied had acute diarrhea
Isaack 1992 [67]	Inappropriate Study Design, investigates nosocomial infections.
Isanaka 2020 [68]	Inappropriate Patient Population. All patients are above 6 months of age.
Islam 2021 [69]	Inappropriate Outcomes. Antibiotic usage was outcome not treatment group. Also all patients have diarrhea.
ISRCTN 2017 [70]	Wrong patient population. Patient age ranged from 2 months to 13 years, unable to obtain subgroup data for under 6 months.
Kabalo 2017 [71]	Inappropriate Patient Population. Patients aged over 6 months.
Lares-Asseff 1999 [72]	Inappropriate Study Design. Study evaluated blood levels for antibiotic not mortality outcome.
Lares-Asseff 2016 [73]	Inappropriate Comparator. Compared different doses of antibiotics to look for toxicity, not mortality.
Lattes 1954 [74]	Inappropriate Study Design and lack of full text.
Lebl 2001 [75]	Inappropriate Patient Population, age range 2 years to 18 years.

Antibiotic as an adjunct to treatment of severe acute malnutrition in infants less than six months of age. A protocol for the systematic review

List of Exclusions and Reasons for Exclusions.

Lelijveld 2021 [76]	Inappropriate Patient Population, age range 6 months to 59 months.
Lepage 1984 [77]	Inappropriate Study Design, patients all have bacterial infection treated with antibiotics.
Lewis 1956 [78]	Inappropriate Patient Population, patients all over 1 year old.
Maataoui 2020 [79]	Inappropriate Patient Population. Patients all over 6 months of age.
Macdougall 1957 [80]	Inappropriate Patient Population. Average age is 2 years.
MacLean 1980 [81]	Inappropriate Comparator. Antibiotic usage compared to enteral feeding.
Maitland 2006 [82]	Inappropriate Patient Population. Median age of 25 months.
Martischnig 1952 [83]	Inappropriate Intervention and lack of full text.
Mathew 2016 [84]	Wrong Patient Population. Patient all aged over 6months.
Mathew 2016 [85]	Wrong Patient Population. Patient all aged over 6months.
Melaku 1999 [86]	Inappropriate Patient Population. Patient median age 5.9 years.
Moschovis 2014 [87]	Inappropriate Study Design. Patients treated for pneumonia.
Mtango 1986 [88]	Inappropriate Intervention. Patients given antibiotics to treat acute respiratory infections.
Muhammad 2020 [89]	Inappropriate Comparator. Compares nutritional treatment with antibiotics usage
Mulholland 1995 [90]	Inappropriate Patient Population. All patients have pneumonia to treat.
Muller 1953 [91]	Inappropriate Study Design and lack of full text.
Murce 1955 [92]	Lack of Full Text.
Muwanguzi 2021 [93]	Inappropriate Patient Population. Children aged 1 year and above.
Nalwanga 2020 [94]	Inappropriate Patient Population. Mean age 14 months.
Nalwanga 2020 [95]	Inappropriate Patient Population. Mean age 14 months.
Nansumba 2018 [96]	Inappropriate Study Design. observational study
Nantanda 2008 [97]	Inappropriate patient population. paediatric patients with pneumonia
NCT 2009 [98]	Inappropriate patient population. paediatric patients with pneumonia
NCT 2009 [99]	Inappropriate Patient Population. children age 6 months to 5 years
NCT 2020 [100]	Inappropriate outcome. studied the effect of prophylactic azithromycin has on the microbiome
NCT 2015 [101]	Inappropriate patient population . hospitalized patients (not necessarily patients with diarrheal illness) receiving prophylactic course of azithromycin (not antibiotics to treat diarrheal illness) at the time of discharge, outcomes of malnourished patients not published
NCT 2012 [102]	Inappropriate patient population . age 6 months to 5 years
Nuzhat 2005 [103]	Inappropriate study design . observational study

Antibiotic as an adjunct to treatment of severe acute malnutrition in infants less than six months of age. A protocol for the systematic review

List of Exclusions and Reasons for Exclusions.

	Inappropriate population . children with a skin disease
O'Brien 2021 [104]	Inappropriate Patient Population . children age 6 to 59 months
O'Brien 2020 [105]	Inappropriate intervention . studied the effect on biannual prophylactic azithromycin (not antibiotics to treat a diarrheal illness) on the mortality rate of malnourished children
O'Brien 2020 [106]	Inappropriate study design . observational study<br>Inappropriate intervention . biannual prophylactic azithromycin (not antibiotics for diarrheal illness)
Page 2013 [107]	Inappropriate Patient Population . children age 6 to 59 months
Pai 1985 [108]	Inappropriate Study Design
Parpia 2020 [109]	Inappropriate Study Design . antibiotics were given prophylactically to the children in the study (not in the treatment of diarrheal illness)<br>Inappropriate patient population: children did not start receiving the prophylactic antibiotics until after 6 months of age
Pinto 2012 [110]	Inappropriate Setting . PICU<br>Inappropriate study design . observational study
Polster 1954 [111]	Lack of Full Text
Pombo 2017 [112]	Inappropriate patient population . patients were hospitalized with pneumonia, not diarrheal illness<br>Inappropriate study design . observational study
Prentice 2013 [113]	Inappropriate Study Design . this paper is a review of various studies on the management of malnutrition in children
Rasul 2006 [114]	Inappropriate Study Design . randomized control trial but no placebo control, controls were admitted to the hospital prior to the study and thus did not receive the WHO protocol<br>Inappropriate patient population . malnourished children age 1 month to 5 years, included hospitalizations for many reasons not just diarrheal illness<br>Inappropriate intervention . compared WHO protocol vs no WHO protocol, so patients in both groups may have received antibiotics and the difference between the groups includes many interventions other than antibiotics
Rawson 2016 [115]	Inappropriate Study Design . letter to the editor
Reed 1996 [116]	Inappropriate Intervention . desired outcome was the difference in incidence of bacteremia and the difference in mortality rate among bacteremic patients between malnourished and adequately nourished children<br>Inappropriate study design . observational study<br>Inappropriate patient population . age 0 to 5 years
Roboz 1955 [117]	Lack of Full Text
Rogawski 2015 [118]	Inappropriate Study design . observational study

Antibiotic as an adjunct to treatment of severe acute malnutrition in infants less than six months of age. A protocol for the systematic review

List of Exclusions and Reasons for Exclusions.

Rokstad 2013 [119]	Inappropriate Patient Population . this paper is a brief review of a study done by Trehan in 2013, which studied a population of children age 6 to 59 months
Rosenberg 1974 [120]	Inappropriate Study Design . review/opinion paper
Roy 2010 [121]	Inappropriate Patient Population . children age 5 to 12 years<br>Inappropriate indication . desired outcome as whether there was a difference in the pharmacokinetics of isoniazid in malnourished children vs adequately nourished children
Sala 1955 [122]	Inappropriate Intervention and lack of full text
Samotra 1985 [123]	Inappropriate Indication . desired outcome was whether or not there was a difference in the pharmacokinetics of gentamicin in malnourished children vs adequately nourished children
Samotra 1986 [124]	Inappropriate Indication . desired outcome was whether there was a difference in the pharmacokinetics of chloramphenicol in malnourished children vs adequately nourished children
Sanogo 2018 [125]	Inappropriate Indication . desired outcome was the level of adherence among healthcare providers to WHO guidelines regarding management (antibiotics, oral rehydration therapy, and zinc) of paediatric diarrheal illness in Bamako, Mali.
Santoro 2002 [126]	Inappropriate Study Design . observational study.<br>Inappropriate indication . desired outcome was to see how many children admitted to the PICU for lower respiratory tract infections needed ICU level care
Schapira 1971 [127]	Lack of Full Text
Sepehr 2009 [128]	Inappropriate Patient population . age 2 to 94 years<br>Inappropriate study design . observational study<br>Inappropriate indication . desired outcome was optimal length of post-operative antibiotic prophylaxis as well as risk factors associated with post-operative infection
Shahira 2002 [129]	Inappropriate Patient Population . population was all pediatric patients at a teaching hospital<br>Inappropriate study design . observational study<br>Inappropriate indication . desired outcome was risk factors for nosocomial infections
Soheir 1981 [130]	Inappropriate Indication . desired outcome was the appropriate dose of chloramphenicol for malnourished children, not mortality
Standing 2018 [131]	Inappropriate indication . studied the proper dose of IV ceftriaxone and PO metronidazole needed to achieve a therapeutic level in malnourished children
Tan 2020 [132]	Inappropriate Indication . studied the use of anthropometric measurements as opposed to clinical signs to identify malnourished children
Taubenslag 1950 [133]	Inappropriate Study Design .
Thame 2001 [134]	Inappropriate Study Design . observational study

Antibiotic as an adjunct to treatment of severe acute malnutrition in infants less than six months of age. A protocol for the systematic review

List of Exclusions and Reasons for Exclusions.

Thorson 1989 [135]	Inappropriate Indication .
Tilg 2013 [136]	Inappropriate Study Design . this paper is a review of a few different studies.<br>Inappropriate patient population . Of interest, it discusses the results from Trehan 2013, which has a patient population of children age 6 months to 59 months
Tornberg-Belanger 2017 [137]	Inappropriate Study Design . observational study<br>Inappropriate topic . studied adherence to antibiotic guidelines of various hospitals
Trehan 2010 [138]	Inappropriate Patient Population . was children age 6 to 59 months<br>Inappropriate study design . observational study
Trehan 2013 [139]	Inappropriate Patient Population . children age 6 to 59 months
Trehan 2016 [140]	Inappropriate Study Design . same exact study as trehan 2013<br>Inappropriate patient population . children age 6 to 59 months
Trehan 2016 [141]	Inappropriate Patient Population . letter to editor
Usman 2019 [142]	Inappropriate Study Design . observational study
Uzan-Yulzari 2021 [143]	Wrong patient population. Age of patients range from birth to 2 years.
Vather 2018 [144]	Inappropriate Study Design . observational study
Verani 2019 [145]	Inappropriate Study Design . observational study.
Viso Gurovich 2003 [146]	Inappropriate study design . observational study.<br>Inappropriate Intervention . studied the outcomes of paediatric patients who received antibiotics to treat various infections, most commonly bronchopneumonia, complicated pneumonia, and conjunctivitis.
Vygen 2013 [147]	Inappropriate Study Design . case series (descriptive study design) looking at the outcomes of malnourished infants who received care at a nutritional rehab center
Walsh 2018 [148]	Inappropriate Intervention . tested the effectiveness of a lactose free legume based nutritional feed to promote the resolution of diarrhea
Weingaertner 1961 [149]	Lack of Full Text
Wittmann 1967 [150]	Inappropriate Patient Population. age rage was 2 months to 2 years and not all were malnourished
Woodd-Walker 1972 [151]	Inappropriate Study Design, inappropriate patient population . age range was from 1 year to 4 years
Zecca 1951 [152]	Lack of Full Text and study is from 1951
BMJ 2013 [153]	Wrong study design. Research news article.
Langdon 2018 [154]	Wrong patient population. Patient ages ranged from 6 weeks to 60 weeks with no subgroup data available for under 6 months.
Journal of Agricultural and Food Chemistry 1954 [155]	Wrong study design. News report article.
Akush 1951 [156]	Wrong indication. Patients all treated for pneumonia.

Antibiotic as an adjunct to treatment of severe acute malnutrition in infants less than six months of age. A protocol for the systematic review

List of Exclusions and Reasons for Exclusions.

Alam 2020 [157]	Wrong study design. All patients treated for diarrhea.

Antibiotic as an adjunct to treatment of severe acute malnutrition in infants less than six months of age. A protocol for the systematic review

List of Exclusions and Reasons for Exclusions.

1. Abate BB, Tilahun BD, Kassie AM, Kassaw MW. Treatment outcome of Severe Acute Malnutrition and associated factors among under-five children in outpatient therapeutics unit in Gubalafto Wereda, North Wollo Zone, Ethiopia, 2019. PLoS One 2020;15(9):e0238231. DOI: 10.1371/journal.pone.0238231.
2. Adam A. [Antibacterial and symbiontic nutrition of infants; a contribution to the problem of dysbacteria]. Arztl Forsch 1954;8(4):I/168-74. (In und).
3. Adem F, Edessa D, Bayissa B, Mohammed Hassen M, Mohammed MA. Treatment Outcomes and Associated Factors in Hospitalised Children with Severe Acute Malnutrition: A Prospective Cohort Study. Pediatric Health Med Ther 2020;11:235-243. DOI: 10.2147/PHMT.S253396.
4. Ahmed T, Ali M, Ullah MM, et al. Mortality in severely malnourished children with diarrhoea and use of a standardised management protocol. The Lancet 1999;353(9168):1919-1922. DOI: https://doi.org/10.1016/S0140-6736(98)07499-6.
5. Ahmed T, Begum B, Badiuzzaman, Ali M, Fuchs G. Management of severe malnutrition and diarrhea. The Indian Journal of Pediatrics 2001;68(1):45-51. DOI: 10.1007/BF02728857.
6. AKBANI Y, BOLME P, LINDBLAD BS, RAHIMTOOLA RJ. CONTROL OF STREPTOMYCIN AND ISONIAZID IN MALNOURISHED CHILDREN TREATED FOR TUBERCULOSIS. Acta Paediatrica 1977;66(2):237-240. DOI: https://doi.org/10.1111/j.1651-2227.1977.tb07840.x.
7. Amorelli Gonzaga M, Alce M, Mesquita da Cunha MO, Da Sil va Peres PR. The efficacy of miocamycin in eutrophic and malnourished children. Jornal de Pediatria 1989;65(6):223-230.
8. Amza A, Kadri B, Nassirou B, et al. A cluster-randomized controlled trial evaluating the effects of mass azithromycin treatment on growth and nutrition in Niger. Am J Trop Med Hyg 2013;88(1):138-143. (In eng). DOI: 10.4269/ajtmh.2012.12-0284.
9. Amza A, Yu SN, Kadri B, et al. Does mass azithromycin distribution impact child growth and nutrition in Niger? A cluster-randomized trial. PLoS Negl Trop Dis 2014;8(9):e3128. (In eng). DOI: 10.1371/journal.pntd.0003128.
10. Angelakis E, Million M, Kankoe S, et al. Abnormal Weight Gain and Gut Microbiota Modifications Are Side Effects of Long-Term Doxycycline and Hydroxychloroquine Treatment. Antimicrobial Agents and Chemotherapy 2014;58(6):3342-3347. DOI: doi:10.1128/AAC.02437-14.
11. Ashorn P. Empirical antibiotic treatment for children suffering from dysentery, cholera, pneumonia, sepsis or severe acute malnutrition. Paediatrics and International Child Health 2018;38(sup1):S1-S2. DOI: 10.1080/20469047.2018.1443546.
12. Ashraf H, Alam N, Gyr N. 126 Randomized Controlled Trial of Day-Care and Hospitalized Management of Severe Pneumonia with Severe Acute Malnutrition in Children in Bangladesh. Archives of Disease in Childhood 2012;97(Suppl 2):A35-A35. DOI: 10.1136/archdischild-2012-302724.0126.
13. Autret E, Gendrel D, Breteau M. [Digestive absorption of gentamicin in malnourished children]. Presse Med 1989;18(15):776. (In fre).
14. Ayelign B, Abebe B, Shibeshi A, et al. Bacterial isolates and their antimicrobial susceptibility patterns among pediatric patients with urinary tract infections. Turk J Urol 2018;44(1):62-69. (In eng). DOI: 10.5152/tud.2017.33678.
15. Ayiyi B, Hazoume FA, Dan V. A review of purulent meningitis in children in Benin. Revue de Pediatrie 1987;23(4):156-160.
16. Aziz F, Patil P. Role of Prophylactic Vitamin K in Preventing Antibiotic Induced Hypoprothrombinemia. The Indian Journal of Pediatrics 2015;82(4):363-367. DOI: 10.1007/s12098-014-1584-3.
17. Berkley JA. Cotrimoxazole Prophylaxis in Severely Malnourished Children. Clinical Trials (US National Library of Medicine) 2009 (https://clinicaltrials.gov/ct2/show/NCT00934492).

Antibiotic as an adjunct to treatment of severe acute malnutrition in infants less than six months of age. A protocol for the systematic review

List of Exclusions and Reasons for Exclusions.

18. Berkley JA, Ngari M, Thitiri J, et al. Daily co-trimoxazole prophylaxis to prevent mortality in children with complicated severe acute malnutrition: a multicentre, double-blind, randomised placebo-controlled trial. Lancet Glob Health 2016;4(7):e464-73. DOI: 10.1016/S2214-109X(16)30096-1.
19. BERKOWITZ FE. Infections in children with severe protein-energy malnutrition. The Pediatric Infectious Disease Journal 1992;11(9):750-759. (https://journals.lww.com/pidj/Fulltext/1992/09000/Infections_in_children_with_severe_protein_energy.15.aspx).
20. Bhatnagar S, Bhan MK, Sazawal S, et al. Efficacy of Massive Dose Oral Gentamicin Therapy in Nonbloody Persistent Diarrhea with Associated Malnutrition. Journal of Pediatric Gastroenterology and Nutrition 1992;15(2):117-124. (https://journals.lww.com/jpgn/Fulltext/1992/08000/Efficacy_of_Massive_Dose_Oral_Gentamicin_Therapy.3.aspx).
21. Bhatnagar S, Bhan MK, Singh KD, Shrivastav R. Prognostic Factors in Hospitalized Children with Persistent Diarrhea: Implications for Diet Therapy. Journal of Pediatric Gastroenterology and Nutrition 1996;23(2):151-158. (https://journals.lww.com/jpgn/Fulltext/1996/08000/Prognostic_Factors_in_Hospitalized_Children_with.10.aspx).
22. Birindwa AM, Manegabe JT, Mindja A, Nordén R, Andersson R, Skovbjerg S. Decreased number of hospitalized children with severe acute lower respiratory infection after introduction of the pneumococcal conjugate vaccine in the Eastern Democratic Republic of the Congo. Pan Afr Med J 2020;37:211. (In eng). DOI: 10.11604/pamj.2020.37.211.22589.
23. Bleakly NT, Axelrod DM, Maldonado YA, Chisti MJ, Kache S. ABSTRACT 597: CLINICAL OUTCOMES AND MORTALITY IN MALNOURISHED CHILDREN BEFORE AND AFTER IMPLEMENTATION OF A PEDIATRIC SEPSIS PROTOCOL. Pediatric Critical Care Medicine 2014;15(4_suppl):135. DOI: 10.1097/01.pcc.0000449323.13704.d1.
24. Boesen I, SamsØE-Jensen T. [Intramuscular penicillin and oral dihydrostreptomycin therapy of acute dyspepsia in bottlefed infants under 7 months]. Nord Med 1953;50(29):1023-4. (In und).
25. Bolme P, Gebre-Ab T, Hadgu P, Meeuwisse G, Stintzing G. Absorption and elimination of penicillin in children with malnutrition. Ethiop Med J 1980;18(4):151-7. (In eng).
26. Bolme P, Eriksson M, Habte D, Paalzow L. Pharmacokinetics of streptomycin in Ethiopian children with tuberculosis and of different nutritional status. European Journal of Clinical Pharmacology 1988;33(6):647-649. DOI: 10.1007/BF00542504.
27. Bolme P, Eriksson M, Paalzow L, Stintzing G, Zerihun G, Woldemariam T. Malnutrition and Pharmacokinetics of Penicillin in Ethiopian Children. Pharmacology & Toxicology 1995;76(4):259-262. DOI: https://doi.org/10.1111/j.1600-0773.1995.tb00140.x.
28. Bravo ME, Arancibia A, Jarpa S, Carpentier PM, Jahn AN. Pharmacokinetics of gentamicin in malnourrished infants. European Journal of Clinical Pharmacology 1982;21(6):499-504. DOI: 10.1007/BF00542045.
29. Bravo IG. The pharmacokinetics of cotrimoxazole sulphonamide in malnourished (marasmic) infants. Pediatric Pharmacology 01-1984;4(3):167-176.
30. Bredow MT, Jackson AA. Community based, effective, low cost approach to the treatment of severe malnutrition in rural Jamaica. Archives of Disease in Childhood 1994;71(4):297-303. DOI: 10.1136/adc.71.4.297.
31. Brodwall K. Uncertain effect of antibiotics for malnourished children. Tidsskrift for Den Norske Laegeforening 2016;136(6):532.
32. Bruhn C. Reducing the inconceivable deficiency: Studies on the use of antibiotics in undernourished African children. Deutsche Apotheker Zeitung 2016;156(30):425-435.

Antibiotic as an adjunct to treatment of severe acute malnutrition in infants less than six months of age. A protocol for the systematic review

List of Exclusions and Reasons for Exclusions.

33. Brunozi NA, David FL. Antibioticoterapia em crianças indígenas e não indígenas desnutridas. ABCS health sci 44(2):85-91.
34. Buchanan N. Letter To The Editor. Acta Paediatrica 1977;66(5):663-663. DOI: https://doi.org/10.1111/j.1651-2227.1977.tb07966.x.
35. Buchanan N, Eyberg C. Intramuscular tobramycin administration in kwashiorkor. S Afr Med J 1978;53(8):273-4. (In eng).
36. Buchanan N, Davis M, Eyberg C. Gentamicin pharmacokinetics in kwashiorkor. British Journal of Clinical Pharmacology 1979;8(5):451-453. DOI: https://doi.org/10.1111/j.1365-2125.1979.tb01024.x.
37. Bunn JEG. Severe acute malnutrition and HIV in African children. Future HIV Therapy 2009;3(6):595-611.
38. Cakir FB, Aydogan G, Timur C, et al. Effects of malnutrition on oxidative burst functions and infection episodes in children with acute lymphoblastic leukemia. International Journal of Laboratory Hematology 2012;34(6):648-654. DOI: https://doi.org/10.1111/j.1751-553X.2012.01451.x.
39. Çaksen H, Cesur Y, Üner A, et al. Urinary tract infection and antibiotic susceptibility in malnourished children. International Urology and Nephrology 2000;32(2):245-247. DOI: 10.1023/A:1007104326689.
40. Caksen H, Arslan S, Cesur Y, Sar S, Celebi V, Kuru M. Urinary tract infection and antibiotic susceptibility in malnourished children. Ceylon Med J 2000;45(2):77-9. (In eng). DOI: 10.4038/cmj.v45i2.8007.
41. Cedrato A, Larguia A, Galan HM. Administration of cefapirin every 12 hours in children with pneumonia: response according to protein nutritional status. Folha Medica 1974;68(1):1-4.
42. Chisti MJ, Salam MA, Bardhan PK, et al. Treatment Failure and Mortality amongst Children with Severe Acute Malnutrition Presenting with Cough or Respiratory Difficulty and Radiological Pneumonia. PLOS ONE 2015;10(10):e0140327. DOI: 10.1371/journal.pone.0140327.
43. Church JA, Fitzgerald F, Walker AS, Gibb DM, Prendergast AJ. The expanding role of co-trimoxazole in developing countries. The Lancet Infectious Diseases 2015;15(3):327-339. DOI: 10.1016/S1473-3099(14)71011-4.
44. Cochlovius A, Daecke K, Loeschke A. [Effect of various antibiotics in infant nutrition disorders]. Dtsch Med Wochenschr 1953;78(37):1266-70. (In und). DOI: 10.1055/s-0028-1114923.
45. Dicko A, Ouedraogo JB, Zongo I, et al. A trial of seasonal malaria chemoprevention plus azithromycin in African children. The American Society of Tropical Medicine and Hygiene 2016;95(5):481. (In English). DOI: 10.4269/ajtmh.abstract2016.
46. Dieng H, Aboussaleh Y, Sbaibi R, Bengueddour R. Evaluation of the care of infants and children under 5 years with severe undernutrition at the CRENAS of Nouakchott, Mauritania. Nutritional Therapy & Metabolism 2014;32:140-145. DOI: 10.5301/NTM.2014.12648.
47. Dubray C, Ibrahim SA, Abdelmutalib M, et al. Treatment of severe malnutrition with 2-day intramuscular ceftriaxone vs 5-day amoxicillin. Annals of Tropical Paediatrics 2008;28(1):13-22. DOI: 10.1179/146532808X270635.
48. Duffau G, Alvarez P, Chala E, Cruz C. [Manifestations of paralytic ileus in infants hospitalized for acute diarrheal syndrome]. Rev Chil Pediatr 1990;61(6):322-6. (In spa).
49. Dutta S, Chowdhary G, Kumar P, Mukhopadhay K, Narang A. Ciprofloxacin Administration to Very Low Birth Weight Babies has no Effect on Linear Growth in Infancy. Journal of Tropical Pediatrics 2005;52(2):103-106. DOI: 10.1093/tropej/fmi070.

Antibiotic as an adjunct to treatment of severe acute malnutrition in infants less than six months of age. A protocol for the systematic review

List of Exclusions and Reasons for Exclusions.

50. Eriksson M, Paalzow L, Bolme P, Mariam TW. Chloramphenicol pharmacokinetics in Ethiopian children of differing nutritional status. European Journal of Clinical Pharmacology 1983;24(6):819-823. DOI: 10.1007/BF00607094.
51. ERIKSSON M, BOLME P, HABTE D, PAALZOW L. INH and Streptomycin in Ethiopian Children with Tuberculosis and Different Nutritional Status. Acta Paediatrica 1988;77(6):890-894. DOI: https://doi.org/10.1111/j.1651-2227.1988.tb10774.x.
52. Ford GW, Belbin R, Jose DG, Vorbach EA, Kirke DK. Growth and Immune Function in Aboriginal Children during Recovery from Malnutrition and Infection. Australian and New Zealand Journal of Medicine 1976;6(4):321-328. DOI: https://doi.org/10.1111/imj.1976.6.4.321.
53. Friedrich MJ. Antibiotics of No Benefit in Treating Children With Malnutrition. JAMA 2016;315(11):1100-1100. DOI: 10.1001/jama.2016.2266.
54. Garcia de Olarte D, Trujillo H, Agudelo N, Nelson JD, Haltalin KC. Treatment of diarrhea in malnourished infants and children. A double-blind study comparing ampicillin and placebo. Am J Dis Child 1974;127(3):379-88. DOI: 10.1001/archpedi.1974.02110220077011.
55. Gardner R, Dwyer C, Morrison C. MINERVA. BMJ: British Medical Journal (Overseas & Retired Doctors Edition) 2011;343(7821):486.
56. Gennaro S, O'Connor C, Marx M. Global Health of Babies and Children. MCN: The American Journal of Maternal/Child Nursing 2017;42(3):132-138. DOI: 10.1097/nmc.0000000000000322.
57. Gensch F, Burmann R, Goertler H. Anstaltsdyspepsien und Kolisubstitution. Dtsch Med Wochenschr 1957;82(20):810-818. (In De).
58. Gernaat HBPE, Dechering WHJC, Voorhoeve HWA. Mortality in Severe Protein-energy Malnutrition at Nchelenge, Zambia. Journal of Tropical Pediatrics 1998;44(4):211-217. DOI: 10.1093/tropej/44.4.211.
59. Ghosh G, Chakraborty S, Ray J, Mukherjee SK. Parenteral ciprofloxacin in persistent diarrhoea in children. J Indian Med Assoc 1995;93(10):382, 384. (In eng).
60. Girma S, Sisay M, Mengistu G, Amare F, Edessa D. Antimicrobial Utilization Pattern in Pediatric Patients in Tertiary Care Hospital, Eastern Ethiopia: The Need for Antimicrobial Stewardship. Hosp Pharm 2018;53(1):44-54. DOI: 10.1177/0018578717737429.
61. Gore-Langton GR, Cairns M, Compaore YD, et al. Effect of adding azithromycin to the antimalarials used for seasonal malaria chemoprevention on the nutritional status of African children. Trop Med Int Health 2020;25(6):740-750. DOI: 10.1111/tmi.13390.
62. Hecht C, Weber M, Grote V, et al. Disease associated malnutrition correlates with length of hospital stay in children. Clin Nutr 2015;34(1):53-9. (In eng). DOI: 10.1016/j.clnu.2014.01.003.
63. Heikens GT, Schofield WN, Christie CD, Gernay J, Dawson S. The Kingston Project. III. The effects of high energy supplement and metronidazole on malnourished children rehabilitated in the community: morbidity and growth. Eur J Clin Nutr 1993;47(3):174-91. (In eng).
64. Heikens GT, Schofield WN, Dawson S. The Kingston Project. II. The effects of high energy supplement and metronidazole on malnourished children rehabilitated in the community: anthropometry. Eur J Clin Nutr 1993;47(3):160-73. (In eng).
65. Hirschhorn N. Can small daily doses of antibiotics prevent the cycle of diarrhea, malabsorption, and malnutrition in children? The American Journal of Clinical Nutrition 1971;24(7):872-875. DOI: 10.1093/ajcn/24.7.872.
66. Howard JE, Beca JP. CLINICAL ASSESSMENT OF HETACILLIN IN ACUTE DIARRHEA OF MALNOURISHED INFANTS. Annals of the New York Academy of Sciences 1967;145(2):436-441. DOI: https://doi.org/10.1111/j.1749-6632.1967.tb50243.x.

Antibiotic as an adjunct to treatment of severe acute malnutrition in infants less than six months of age. A protocol for the systematic review

List of Exclusions and Reasons for Exclusions.

67. Isaack H, Mbise RL, Hirji KF. Nosocomial bacterial infections among children with severe protein energy malnutrition. East Afr Med J 1992;69(8):433-6. (In eng).
68. Isanaka S, Grantz KH, Berthé F, Schaefer M, Adehossi E, Grais RF. Extended Follow-up From a Randomized Clinical Trial of Routine Amoxicillin in the Treatment of Uncomplicated Severe Acute Malnutrition in Niger. JAMA Pediatrics 2020;174(3):295-297. DOI: 10.1001/jamapediatrics.2019.5189.
69. Islam MR, Nuzhat S, Fahim SM, et al. Antibiotic exposure among young infants suffering from diarrhoea in Bangladesh. Journal of Paediatrics and Child Health 2021;57(3):395-402. DOI: https://doi.org/10.1111/jpc.15233.
70. ISRCTN. A study to compare antibiotics used to treat children with severe acute malnutrition. 2017 (https://trialsearch.who.int/?TrialID=ISRCTN18051843).
71. Kabalo MY, Seifu CN. Treatment outcomes of severe acute malnutrition in children treated within Outpatient Therapeutic Program (OTP) at Wolaita Zone, Southern Ethiopia: retrospective cross-sectional study. Journal of Health, Population and Nutrition 2017;36(1):7. DOI: 10.1186/s41043-017-0083-3.
72. Lares-Asseff I, Lugo-Goytia G, Pérez-Guillé MG, et al. [Bayesian prediction of chloramphenicol blood levels in children with sepsis and malnutrition]. Rev Invest Clin 1999;51(3):159-65. (In spa).
73. Lares-Asseff I, Pérz-Guillé MG, Camacho Vieyra GA, Pérez AG, Peregrina NB, Lugo Goytia G. Population Pharmacokinetics of Gentamicin in Mexican Children With Severe Malnutrition. The Pediatric Infectious Disease Journal 2016;35(8):872-878. DOI: 10.1097/inf.0000000000001204.
74. Lattes E, Piovano L. [Treatment of acute infant nutrition disorders and tests of sensitivity to antibiotics; clinical considerations]. Minerva Pediatr 1954;6(7):247-8. (In und).
75. Lebl J, Zahradníková M, Bartosová J, Zemková D, Pechová M, Vávrová V. Insulin-like growth factor-I and insulin-like growth factor-binding protein-3 in cystic fibrosis: a positive effect of antibiotic therapy and hyperalimentation. Acta Paediatr 2001;90(8):868-72. (In eng).
76. Lelijveld N, Godbout C, Krietemeyer D, et al. Treating high-risk moderate acute malnutrition using therapeutic food compared with nutrition counseling (Hi-MAM Study): a cluster-randomized controlled trial. The American Journal of Clinical Nutrition 2021;114(3):955-964. DOI: 10.1093/ajcn/nqab137.
77. Lepage P, Bogaerts J, Nsengumuremyi F, et al. Severe multiresistant Salmonella typhimurium systemic infections in Central Africa – clinical features and treatment in a paediatric department. Journal of Antimicrobial Chemotherapy 1984;14(suppl_B):153-159. DOI: 10.1093/jac/14.suppl_B.153.
78. Lewis RA, Bhagat MP, Wagle MM, Kulkarni BS, Satoskar RS. Antibiotic Dietary Supplements in the Therapy of Childhood Protein Mainutrition. The American Journal of Tropical Medicine and Hygiene 1956;5(3):483-496. (In English). DOI: 10.4269/ajtmh.1956.5.483.
79. Maataoui N, Langendorf C, Berthe F, et al. Increased risk of acquisition and transmission of ESBL-producing Enterobacteriaceae in malnourished children exposed to amoxicillin. Journal of Antimicrobial Chemotherapy 2019;75(3):709-717. DOI: 10.1093/jac/dkz487.
80. MACDOUGALL LG. THE EFFECT OF AUREOMYCIN ON UNDERNOURISHED AFRICAN CHILDREN. Journal of Tropical Pediatrics 1957;3(2):74-81. DOI: 10.1093/oxfordjournals.tropej.a057461.
81. MacLean WC, Jr., Lopez de Romaña G, Massa E, Graham GG. Nutritional management of chronic diarrhea and malnutrition: Primary reliance on oral feeding. The Journal of Pediatrics 1980;97(2):316-323. DOI: 10.1016/S0022-3476(80)80507-5.
82. Maitland K, Berkley JA, Shebbe M, Peshu N, English M, Newton CR. Children with severe malnutrition: can those at highest risk of death be identified with the WHO protocol? PLoS Med 2006;3(12):e500. (In eng). DOI: 10.1371/journal.pmed.0030500.

Antibiotic as an adjunct to treatment of severe acute malnutrition in infants less than six months of age. A protocol for the systematic review

List of Exclusions and Reasons for Exclusions.

83. Martischnig E. [Antibiotic therapy of severe nutritional disorders in infants]. Ann Paediatr 1952;179(3):168-87. (In und).
84. Mathew JL. Does Routine Antibiotic Therapy Benefit Children With Severe Acute Malnutrition?: Evidence-based Medicine Viewpoint. Indian Pediatr 2016;53(4):329-32. (In eng). DOI: 10.1007/s13312-016-0846-8.
85. Mathew JL, Rai R. Does routine antibiotic therapy benefit children with severe acute malnutrition? Indian Pediatrics 2016;53(4):329-333. DOI: 10.1007/s13312-016-0846-8.
86. Melaku A, Lulseged S. Chronic suppurative otitis media in a children's hospital in Addis Ababa, Ethiopia. Ethiop Med J 1999;37(4):237-46. (In eng).
87. Moschovis PP, Addo-Yobo EOD, Banajeh S, et al. Stunting is associated with poor outcomes in childhood pneumonia. Tropical medicine & international health : TM & IH 2015;20(10):1320-1328. (In eng). DOI: 10.1111/tmi.12557.
88. Mtango FDE, Neuvians D. Acute respiratory infections in children under five years. Control project in Bagamoyo District, Tanzania. Transactions of The Royal Society of Tropical Medicine and Hygiene 1986;80(6):851-858. DOI: 10.1016/0035-9203(86)90241-5.
89. Muhammad A SY, Nisar MI, Baloch B, Yazdani AT, Yazdani N, Jehan F. . Nutritional support for lactating women with or without azithromycin for infants compared to breastfeeding counseling alone in improving the 6-month growth outcomes among infants of peri-urban slums in Karachi, Pakistan-the protocol for a multiarm assessor-blinded randomized controlled trial Trials 2020;1(21):756.
90. MULHOLLAND EK, FALADE AG, CORRAH PT, et al. A randomized trial of chloramphenicol vs. trimethoprim-sulfamethoxazole for the treatment of malnourished children with community-acquired pneumonia. The Pediatric Infectious Disease Journal 1995;14(11):959-964. (https://journals.lww.com/pidj/Fulltext/1995/11000/A_randomized_trial_of_chloramphenicol_vs_.7.aspx).
91. Muller R. [Antibiotic therapy of nutrition disorders]. Kinderarztl Prax 1953:195-200. (In ger).
92. Murce A. [Current problems concerning etiology and therapy of acute nutritional disorders in early infancy; effect of chemotherapeutic agents and antibiotics]. Rev Bras Med 1955;12(5):306-13. (In por).
93. Muwanguzi E, Oboi JE, Nabbamba A, Wanyama R. Treatment outcome and associated factors for severely malnourished children (1–5 years) admitted to Lacor Hospital and Gulu Regional Referral Hospital in Uganda. Journal of Nutritional Science 2021;10:e33. DOI: 10.1017/jns.2021.11.
94. Nalwanga D, Musiime V, Kizito S, et al. Mortality among children under five years admitted for routine care of severe acute malnutrition: a prospective cohort study from Kampala, Uganda. BMC Pediatrics 2020;20(1):182. DOI: 10.1186/s12887-020-02094-w.
95. Nalwanga D, Musiime V, Kizito S, Kiggundu JB, Musoke P, Tumwine JK. Mortality among Children Hospitalized for Routine Care of Severe Acute Malnutrition. Pediatrics 2020;146(1 MeetingAbstract):299-299. DOI: 10.1542/peds.146.1_MeetingAbstract.299.
96. Nansumba M, Kumbakumba E, Orikiriza P, et al. Treatment outcomes and tolerability of the revised WHO anti-tuberculosis drug dosages for children. The International Journal of Tuberculosis and Lung Disease 2018;22(2):151-157. DOI: 10.5588/ijtld.17.0535.
97. Nantanda R, Hildenwall H, Peterson S, Kaddu-Mulindwa D, Kalyesubula I, K.Tumwine J. Bacterial aetiology and outcome in children with severe pneumonia in Uganda. Annals of Tropical Paediatrics 2008;28(4):253-260. DOI: 10.1179/146532808X375404.
98. International Centre for Diarrhoeal Disease Research B. Trial in Childhood Pneumonia With Malnutrition. 2009 (https://clinicaltrials.gov/ct2/show/NCT00968370).

Antibiotic as an adjunct to treatment of severe acute malnutrition in infants less than six months of age. A protocol for the systematic review

List of Exclusions and Reasons for Exclusions.

99. Medicine WUSo. Effectiveness of Oral Antibiotics in the Treatment of Severe Acute Malnutrition. 2009 (https://clinicaltrials.gov/ct2/show/NCT01000298).
100. University of California SF. Gut and Azithromycin Mechanisms in Infants and Children II (GAMINII). 2020 (https://clinicaltrials.gov/ct2/show/NCT04315272).
101. Washington Uo. Azithromycin to Prevent Post-discharge Morbidity and Mortality in Kenyan Children (Toto Bora). 2015 (https://clinicaltrials.gov/ct2/show/NCT02414399).
102. Epicentre. The Effect of Routine Antibiotic Use in the Outpatient Treatment of Severely Malnourished Children Without Complications. 2012 (https://clinicaltrials.gov/ct2/show/NCT01613547).
103. Yasmeen N, Khan MR. Spectrum of common childhood skin diseases: a single centre experience. J Pak Med Assoc 2005;55(2):60-3. (In eng).
104. O'Brien KS, Sié A, Dah C, et al. Azithromycin for uncomplicated severe acute malnutrition: study protocol for a pilot randomized controlled trial. Pilot and Feasibility Studies 2021;7(1):97. DOI: 10.1186/s40814-021-00836-w.
105. O'Brien KS, Arzika AM, Maliki R, et al. Biannual azithromycin distribution and child mortality among malnourished children: A subgroup analysis of the MORDOR cluster-randomized trial in Niger. PLoS Med 2020;17(9):e1003285. DOI: 10.1371/journal.pmed.1003285.
106. Kieran S. O'Brien AMA, Ramatou Maliki,Farouk Manzo,Alio K. Mamkara,Elodie Lebas,Catherine Cook,Robin L. Bailey,Sheila K. West,Catherine E. Oldenburg,Travis C. Porco,Benjamin Arnold,Jeremy D. Keenan,Thomas M. Lietman. The role of nutritional status in the efficacy of azithromycin to reduce child mortality in Niger: A subgroup analysis of the mordor trial. American Journal of Tropical Medicine and Hygiene 2019. DOI: 10.4269/ajtmh.abstract2019.
107. Page A-L, de Rekeneire N, Sayadi S, et al. Infections in Children Admitted with Complicated Severe Acute Malnutrition in Niger. PLOS ONE 2013;8(7):e68699. DOI: 10.1371/journal.pone.0068699.
108. Pai PM, Desai M, Vachharajani A. Drug therapy in malnourished children. 1985;25:25-28.
109. Parpia TC, Elwood SE, Scharf RJ, et al. Baseline Characteristics of Study Participants in the Early Life Interventions for Childhood Growth and Development in Tanzania (ELICIT) Trial. The American Journal of Tropical Medicine and Hygiene 2020;103(4):1397-1404. (In English). DOI: 10.4269/ajtmh.19-0918.
110. Pinto V. American Academy of Pediatrics Section on Critical Care Scientific Program Scientific Abstracts and Posters AAP National Conference and Exhibition October 21, 2012 – New Orleans, LA. Pediatric Critical Care Medicine 2012;13(6):704-710. DOI: 10.1097/PCC.0b013e31826df088.
111. Polster H. [Chloramphenicol treatment of acute nutrition disorders]. Kinderarztl Prax 1954;22(3):109-13. (In und).
112. Pombo-March P. Clinical profile of children and adolescents with and without comorbidities hospitalized with community acquired pneumonia. Pediatric Pulmonology 2017;52(S46):S100-S176. DOI: https://doi.org/10.1002/ppul.23731.
113. Prentice AM, Nabwera H, Kwambana B, Antonio M, Moore SE. Microbes and the Malnourished Child. Science Translational Medicine 2013;5(180):180fs11-180fs11. DOI: doi:10.1126/scitranslmed.3006212.
114. Rasul C, Aa M, Hasanuzzaman. Outcome of management of severe malnutrition in a medical institution. Paediatrics, Child and Adolescent Health Journal 2006;Paediatrics, Child and Adolescent Health Journal (Singapore) 2006; 46: 156-60.
115. Grais TMRLMAHITKSMMSIEAR. Amoxicillin for Severe Acute Malnutrition in Children. New England Journal of Medicine 2016;375:190-192. (https://www.nejm.org/doi/pdf/10.1056%2FNEJMc1605388).

Antibiotic as an adjunct to treatment of severe acute malnutrition in infants less than six months of age. A protocol for the systematic review

List of Exclusions and Reasons for Exclusions.

116. Reed RP, Wegerhoff FO, Rothberg AD. Bacteraemia in malnourished rural African children. Annals of Tropical Paediatrics 1996;16(1):61-68. DOI: 10.1080/02724936.1996.11747805.
117. Roboz P, Kadar M. [Increase of weight in infant dystrophy following isoniazid therapy]. Orv Hetil 1955;96(9):234-5. (In hun).
118. Rogawski ET, Westreich DJ, Adair LS, et al. Early Life Antibiotic Exposure Is Not Associated with Growth in Young Children of Vellore, India. J Pediatr 2015;167(5):1096-102.e3. (In eng). DOI: 10.1016/j.jpeds.2015.08.015.
119. Rokstad IS. Antibiotics in severe malnutrition in children. 2013. DOI: 10.4045/tidsskr.13.0366.
120. Rosenberg IH, Beisel WR, Gordon JE, et al. Infant and child enteritis-malabsorption-malnutrition: the potential of limited studies with low-dose antibiotic feeding. The American Journal of Clinical Nutrition 1974;27(3):304-309. DOI: 10.1093/ajcn/27.3.304.
121. Roy V, Gupta D, Gupta P, Sethi GR, Mishra TK. Pharmacokinetics of isoniazid in moderately malnourished children with tuberculosis. Int J Tuberc Lung Dis 2010;14(3):374-6. (In eng).
122. Sala F. [Observations on the use of combined chloramphenicol-sulfonamide therapy in acute nutritional disorders in pediatrics]. Gazz Med Ital 1955;114(3):54-8. (In ita).
123. Samotra K, Gupte S, Raina RK. Pharmacokinetics of gentamicin in protein-energy malnutrition. European Journal of Clinical Pharmacology 1985;29(2):255-256. DOI: 10.1007/BF00547433.
124. Samotra K, Gupte S, Raina RK. Effect of malnutrition on chloramphenicol kinetics in Indian children. Zhongguo Yao Li Xue Bao 1986;7(2):162-4. (In eng).
125. Sanogo D, Keita A, Roose A, et al. MANAGEMENT OF DIARRHEA IN MALI: ASSESSMENT OF THE ADHERENCE TO WHO RECOMMENDATIONS IN THE VACCINE IMPACT ON DIARRHEA IN AFRICA (VIDA) STUDY. AMERICAN JOURNAL OF TROPICAL MEDICINE AND HYGIENE: AMER SOC TROP MED & HYGIENE 8000 WESTPARK DR, STE 130, MCLEAN, VA 22101 USA; 2018:591-591.
126. SANTORO A, FERREIRA E, FERRARI AM. Infecciones respiratorias agudas bajas en niños menores de tres años: Referencia a unidades de cuidado intensivo. Archivos de Pediatría del Uruguay 2002;73:196-202. (http://www.scielo.edu.uy/scielo.php?script=sci_arttext&pid=S1688-12492002000400003&nrm=iso).
127. Schapira Y, Richtie J. [Combined therapy of Pseudomonas infection and severe malnutrition]. Harefuah 1971;80(11):608-10. (In heb).
128. Sepehr A, Santos B-JG, Chou C, et al. Antibiotics in head and neck surgery in the setting of malnutrition, tracheotomy, and diabetes. The Laryngoscope 2009;119(3):549-553. DOI: https://doi.org/10.1002/lary.20078.
129. Shahira S. BHAR, El Metwaly; Shoikar, Abdel Salam. Bacterial nosocomial infections among children admitted to wards at El-Shatby university children's hospital. Alexandria Journal of Pediatrics 2002;16:297-304.
130. Soheir M. DH, Shatla; Mona, Salem; Laila, El Sayed; Tahani E.S., Kholeif. Pharmacokinetics of cefazolin and chloramphenicol in protein-energy malnuirition. Journal of the Egyptian Society of Pharmacology and Experimental Therapeutics 1981;2:91-100.
131. Standing JF, Ongas MO, Ogwang C, et al. Dosing of Ceftriaxone and Metronidazole for Children With Severe Acute Malnutrition. Clinical Pharmacology & Therapeutics 2018;104(6):1165-1174. DOI: https://doi.org/10.1002/cpt.1078.
132. Tan R, Kagoro F, Levine GA, et al. Clinical Outcome of Febrile Tanzanian Children with Severe Malnutrition Using Anthropometry in Comparison to Clinical Signs. The American Journal of Tropical Medicine and Hygiene 2020;102(2):427-435. (In English). DOI: 10.4269/ajtmh.19-0553.

Antibiotic as an adjunct to treatment of severe acute malnutrition in infants less than six months of age. A protocol for the systematic review

List of Exclusions and Reasons for Exclusions.

133. Taubenslag L. [Streptomycin in acute nutritional disorders of infants in first half-year]. Dia Med 1950;22(48):1965-9. (In und).

134. Thame M, Stephen C, Wilks R, Forrester TE. The appropriateness of the current antibiotic empiric therapy based on the bacteria isolated from severely malnourished Jamaican children. West Indian Med J 2001;50(2):140-3. (In eng).

135. Thorson SM. Multiple-drug resistant Salmonella enteritis in malnourished children: Dramatic response to cefotaxime. 1989;11:279-287.

136. Tilg H, Moschen AR. Malnutrition and microbiota—a new relationship? Nature Reviews Gastroenterology & Hepatology 2013;10(5):261-262. DOI: 10.1038/nrgastro.2013.48.

137. Tornberg-Belanger S, Tickell K, Mangale D, et al. Pediatric inpatient antibiotic prescription practices in the chain network hospitals at baseline. American Society of Tropical Medicine and Hygiene; 2017.

138. Trehan I, Amthor RE, Maleta K, Manary MJ. Evaluation of the routine use of amoxicillin as part of the home-based treatment of severe acute malnutrition. Tropical Medicine & International Health 2010;15(9):1022-1028. (https://doi.org/10.1111/j.1365-3156.2010.02580.x). DOI: https://doi.org/10.1111/j.1365-3156.2010.02580.x.

139. Trehan I, Goldbach HS, LaGrone LN, et al. Antibiotics as Part of the Management of Severe Acute Malnutrition. New England Journal of Medicine 2013;368(5):425-435. DOI: 10.1056/NEJMoa1202851.

140. Trehan I, Goldbach HS, LaGrone LN, et al. Antibiotics as part of the management of severe acute malnutrition. Malawi Med J 2016;28(3):123-130. (In eng).

141. Trehan I. Amoxicillin for Severe Acute Malnutrition in Children. New England Journal of Medicine 2016;375(2):190-192. DOI: 10.1056/NEJMc1605388.

142. Farina Usman SS, Muhammad Amir, Muhammad Nadeem Chohan, Muhammad Touseef. Efficacy of Ampicillin and Gentamycin Combination Therapy in Children Having Severe Acute Malnutrition Presenting with Acute Diarrhea at Civil Hospital Hyderabad. Medical Forum Monthly 2019.

143. Atara Uzan-Yulzari OT, Anna Belogolovski, Oren Ziv, Christina Kunz, Sarah Perschbacher, Hadar Neuman, Edoardo Pasolli, Aia Oz, Hila Ben-Amram, Himanshu Kumar, Helena Ollila, Anne Kaljonen, Erika Isolauri, Seppo Salminen, Hanna Lagström, Nicola Segata, Itai Sharon, Yoram Louzoun, Regina Ensenauer,. Neonatal antibiotic exposure impairs child growth during the first six years of life by perturbing intestinal microbial colonization. Nature Communications 2021. DOI: 10.1038/s41467-020-20495-4.

144. Vather A, McKerrow NH, Morgan ME. Factors underlying the changing of antibiotics in a paediatric intensive care unit in Pietermaritzburg, KwaZulu-Natal2018.

145. Verani. Abstract Book. The American Journal of Tropical Medicine and Hygiene 2019;101(5_Suppl):1-668. (In English). DOI: 10.4269/ajtmh.abstract2019.

146. Gurovich F, Gómez-Oliván L, Velasco JM. Assessment of the use of chloramphenicol at the Neonatal Unit of a Mexican Paediatric Hospital. Pharmaceutical Care Espana 2003;5:182-185.

147. Vygen SB, Roberfroid D, Captier V, Kolsteren P. Treatment of Severe Acute Malnutrition in Infants Aged <6 Months in Niger. The Journal of Pediatrics 2013;162(3):515-521.e3. DOI: 10.1016/j.jpeds.2012.09.008.

148. Walsh K, Calder N, Olupot-Olupot P, et al. Modifying Intestinal Integrity and MicroBiome in Severe Malnutrition with Legume-Based Feeds (MIMBLE 2.0): protocol for a phase II refined feed and intervention trial [version 1; peer review: 2 approved]. Wellcome Open Research 2018;3(95). DOI: 10.12688/wellcomeopenres.14706.1.

149. Weingaertner L. [A new antibiotic, colistin, in the treatment of infant nutrition disorders]. Munch Med Wochenschr 1961;103:1628-31. (In ger).

Antibiotic as an adjunct to treatment of severe acute malnutrition in infants less than six months of age. A protocol for the systematic review

List of Exclusions and Reasons for Exclusions.

150. Wittmann W, Moodie AD, Hansen JD. A comparison of two antibiotic regimens in the treatment of gastroenteritis in malnourished children with a one-year follow-up of their subsequent progress. S Afr Med J 1967;41(16):414-7. (In eng).

151. WOODD-WALKER RB, HANSEN JDL, SAUNDERS SJ. THE IN VITRO UPTAKE OF LYSINE AND ALANINE BY HUMAN JEJUNAL MUCOSA IN PROTEIN-CALORIE MALNUTRITION, IN GASTROENTERITIS AND AFTER NEOMYCIN. Acta Paediatrica 1972;61(2):140-144. DOI: https://doi.org/10.1111/j.1651-2227.1972.tb15917.x.

152. Zecca E. [Streptomycin therapy in acute and chronic nutrition disorders in hospital]. Lattante 1951;22(9):536-40. (In und).

153. BMJ. Treating malnutrition with antibiotics. 2013:356. (https://regroup-production.s3.amazonaws.com/documents/ReviewReference/357822763/Treating%20malnutrition%20with%20antibiotics.pdf?AWSAccessKeyId=AKIAJBZQODCMKJA4H7DA&Expires=1643053445&Signature=SvDdx2aaEu%2B8i5CwDvlRAgoL52s%3D).

154. Amy Elizabeth Langdon XS, Sheila Isanaka, Gautam, Dantas. Prophylactic broad-spectrum antibiotics for childhood malnutrition. Journal of clinical and translational science 2018:46. (https://regroup-production.s3.amazonaws.com/documents/ReviewReference/357814158/Langdon%202018%20%20.pdf?AWSAccessKeyId=AKIAJBZQODCMKJA4H7DA&Expires=1643053619&Signature=UGJETRPj9mELVYWfjDUkVF%2Fhmqw%3D).

155. News: Antibiotic Supplements Overcome Growth Failure in School Children. Journal of Agricultural and Food Chemistry 1954;2(23):1148-1148. DOI: 10.1021/jf60043a605.

156. [Antibiotic albomycin in the treatment of pneumonias and toxemias in infants during the first year of life]. Feldsher Akush 1951;12:38. (In und).

157. Alam T, Ahmed D, Ahmed T, et al. A double-blind placebo-controlled trial of azithromycin to reduce mortality and improve growth in high-risk young children with non-bloody diarrhoea in low resource settings: the Antibiotics for Children with Diarrhoea (ABCD) trial protocol. Trials 2020;21(1):71. DOI: 10.1186/s13063-019-3829-y.
