# Supplementary material for: Routine antibiotics for infants less than 6 months of age with growth failure/faltering: a systematic review
Source: BMJ Open. 2023 May 10;13(5):e071393. doi: 10.1136/bmjopen-2022-071393 (PMC10174012; doi:10.1136/bmjopen-2022-071393)
Supplement: Author's manuscript [file bmjopen-2022-071393.draft_revisions.pdf]

BMJ Open

BMJ Open is committed to open peer review. As part of this commitment we make the peer review history of every article we publish publicly available.

When an article is published we post the peer reviewers' comments and the authors' responses online. We also post the versions of the paper that were used during peer review. These are the versions that the peer review comments apply to.

The versions of the paper that follow are the versions that were submitted during the peer review process. They are not the versions of record or the final published versions. They should not be cited or distributed as the published version of this manuscript.

BMJ Open is an open access journal and the full, final, typeset and author-corrected version of record of the manuscript is available on our site with no access controls, subscription charges or pay-per-view fees (http://bmjopen.bmj.com).

If you have any questions on BMJ Open's open peer review process please email info.bmjopen@bmj.com

BMJ Open

Routine antibiotics for infants less than six months of age with growth failure/faltering. A systematic review

Journal:	*BMJ Open*
Manuscript ID	bmjopen-2022-071393
Article Type:	Original research
Date Submitted by the Author:	03-Jan-2023
Complete List of Authors:	Imdad, Aamer; SUNY Upstate Medical University, Pediatrics<br>Chen, Fanny F; Upstate University Hospital Department of Medicine,<br>François, Melissa; SUNY Upstate Medical University,<br>Sana, Momal; Monash Health,<br>Tanner-Smith, Emily; University of Oregon,<br>Smith, Abigail; SUNY Upstate Medical University, Health Science Library<br>Tsistinas, Olivia; SUNY Upstate Medical University<br>Das, Jai K.; Aga Khan University, Division of Women and Child Health<br>Bhutta, Zulfiqar; The Aga Khan University, Division of Women and Child Health; Hospital for Sick Children Research Institute, Global Child Health
Keywords:	Nutrition < TROPICAL MEDICINE, INFECTIOUS DISEASES, PUBLIC HEALTH

SCHOLARONE™
Manuscripts

I, the Submitting Author has the right to grant and does grant on behalf of all authors of the Work (as defined in the below author licence), an exclusive licence and/or a non-exclusive licence for contributions from authors who are: i) UK Crown employees; ii) where BMJ has agreed a CC-BY licence shall apply, and/or iii) in accordance with the terms applicable for US Federal Government officers or employees acting as part of their official duties; on a worldwide, perpetual, irrevocable, royalty-free basis to BMJ Publishing Group Ltd ("BMJ") its licensees and where the relevant Journal is co-owned by BMJ to the co-owners of the Journal, to publish the Work in this journal and any other BMJ products and to exploit all rights, as set out in our licence.

The Submitting Author accepts and understands that any supply made under these terms is made by BMJ to the Submitting Author unless you are acting as an employee on behalf of your employer or a postgraduate student of an affiliated institution which is paying any applicable article publishing charge ("APC") for Open Access articles. Where the Submitting Author wishes to make the Work available on an Open Access basis (and intends to pay the relevant APC), the terms of reuse of such Open Access shall be governed by a Creative Commons licence – details of these licences and which Creative Commons licence will apply to this Work are set out in our licence referred to above.

Other than as permitted in any relevant BMJ Author's Self Archiving Policies, I confirm this Work has not been accepted for publication elsewhere, is not being considered for publication elsewhere and does not duplicate material already published. I confirm all authors consent to publication of this Work and authorise the granting of this licence.

For peer review only

Routine antibiotics for infants less than six months of age with growth failure/faltering. A systematic review

Aamer Imdad, Fanny F Chen, Melissa François, Momal Sana, Emily Tanner-Smith, Abigail Smith, Olivia Tsistinas, Jai Das, Zulfiqar Ahmed Bhutta

Authors' information

Contact Author
Aamer Imdad, MBBS, MPH
Assistant Professor of Pediatrics
Division of Pediatric Gastroenterology, Hepatology and Nutrition
SUNY Upstate Medical University, Syracuse, NY
725 Irving Street, Syracuse, NY 13210
Email: imdada@upstate.edu
Phone: 315-464-8444
Fax: 315-464-8443

Fanny Chen
SUNY Upstate Medical University
ChenF@upstate.edu

Melissa Francois
SUNY Upstate Medical University
FrancoMe@upstate.edu

Momal Sana
Quaid e Azam Medical College
momalsana42@gmail.com

Emily Tanner-Smith
University of Oregon
etanners@uoregon.edu

Abigail Smith
SUNY Upstate Medical University
SmithAb@upstate.edu

Olivia Tsistinas
SUNY Upstate Medical University
TsistiJO@upstate.edu

Jai Das
Aga Khan University
Jai.das@aku.edu

Zulfiqar Ahmed Bhutta
Institute for Global Health & Development, The Aga Khan University

For peer review only

zulfiqar.bhutta@aku.edu

Key Words: Antibiotics, wasting, malnutrition, growth failure

For peer review only

Abstract

Objective: This systematic review commissioned by WHO aimed to synthesize evidence from current literature on the effects of systematically given, routine use of antibiotics for infants under six months of age with growth failure/faltering.

Settings: Low and Middle income countries

Participants: The study population was infants less than six months of age with growth failure/faltering.

Intervention: The intervention group was infants who received no antibiotics or antibiotics other than recommended in 2013 guidelines by WHO to treat childhood severe acute malnutrition. The comparison group was infants who received antibiotics according to the aforementioned guidelines.

Primary and Secondary outcomes: The primary outcome was all-cause mortality and secondary outcomes: clinical deterioration, antimicrobial resistance, recovery from co-morbidity, adverse events, markers of intestinal inflammation, markers of systemic inflammation, hospital-acquired infections, non-response. The Grading of Recommendations Assessment, Development, and Evaluation (GRADE) approach was considered to report the overall evidence quality for an outcome.

Results: We screened 5137 titles and abstract and reviewed full text of 157 studies. None of the studies from literature search qualified to answer the question for this systematic review.

Conclusions: There is paucity of evidence on routine use of antibiotics for treatment of malnutrition in infants less than 6 months of age. Future studies with adequate sample sizes are needed to assess the potential risks and benefits from the use of antibiotics in malnourished infants less than 6 months of age.

Systematic Review registration: This study was registered at PROSPERO website (ID=CRD42021277073)

Strengths and Limitations

- This World Health Organization funded systematic review was conducted by following the standard methods of Cochrane Collaboration.
- Even though data were available for use of antibiotics in severely malnourished children 6-59 months of age, no randomized trials were found in infants less than six months of age
- The work was limited due to lack of studies in this age group. Future studies with large sample size are needed to assess the efficacy and safety of antibiotics in malnourished infants less than six months of age.

For peer review only

Main Manuscript

Introduction

The World Health Organization (WHO) and the United Nations Children's Fund (UNICEF) estimate that nearly 14 million children suffer from severe wasting (low weight for height) worldwide[1]. Infants less than six months of age are particularly vulnerable to the effects of inadequate nutrition. Higher mortality rates secondary to growth failure are seen in this age group compared to older infants and children[2,3]. Despite excess mortality risk and increasing prevalence of wasting in this population, limited studies exist to guide the management of young infants with growth failure and faltering[2-4]. Malnutrition in children increases the risk of severe infections in children and triples the risk of mortality from pneumonia, measles, or diarrhea[5]. Therefore, the current practice for children 6 months to 5 years of age with wasting is to prescribe routine antibiotics when they get into a nutrition program, inpatient or outpatient[5]. However, current recommendations state that the same general medical care should be used for infants with SAM who are less than six months of age as infants above six months of age, even though there is limited evidence to support this recommendation[2]. Furthermore, even though antibiotics have been shown to be effective in children 6-59 months of age with SAM, this practice in infants has the potential to harm due to recently identified risks of antibiotic use in infancy, including the diminishment of infant gut microbiome[6], future development of obesity, allergic disorders[7], and autoimmune disorders[8]. The urgency in more targeted management guidelines is further underscored by the physiological differences in renal and gastrointestinal function in infants compared to older children[8]. The WHO recently started the process of guideline development for the prevention and treatment of wasting in children, including growth failure/faltering in infants less than six months. The aim of this systematic review was to synthesize evidence from current literature on the effect of systematically given, routine use of antibiotics for infants less than six months of age with growth failure/faltering.

OBJECTIVE

Primary Objective: In infants <6 months with growth failure/faltering, what are the effects of no routine antibiotics or different approaches (e.g., types of antibiotics, doses) compared to routine antibiotics following treatment protocols in the 2013 WHO guidelines[2] on the morbidity and mortality outcomes?

METHODS AND ANALYSIS

This systematic review was conducted according to methods described in Cochrane Handbook[9] and reported using PRISMA guidelines 2020[10]. We registered the protocol on the PROSPERO website (ID=CRD42021277073).

Types of studies: We considered both individual and cluster randomized trials. We also considered non-randomised trials and cohort studies with a controlled arm. We excluded case-control studies, case reports, case series, and commentaries.

Population

The population of interest was infants under six months of age with growth failure/faltering. As there is no standard definition of growth failure/faltering, we considered author definitions. We considered studies irrespective of whether they were done in community or hospital settings. We considered studies that included infants infected with HIV. We considered studies with low birth weight or preterm infants; however, we excluded studies on infants admitted to neonatal intensive care units. We excluded studies that only included infants with congenital anomalies.

For peer review only

Intervention
We considered all antibiotic treatments given systemically, such as amoxicillin, Augmentin, cephalosporins, and macrolides. We considered studies irrespective of dosage, frequency, duration, or route of administration; however, topical application of antibiotics was not considered. We considered studies if antibiotics were given empirically at the time of diagnosis of growth failure or faltering, irrespective of the indication, e.g. to treat an infection. We excluded studies where antibiotics were given for the prevention of wasting in otherwise healthy infants specifically non-malnourished children or infants with no growth failure/faltering. We also excluded studies where antibiotics were given for other reasons, such as suspected serious bacterial infections in otherwise healthy infants specifically non-malnourished children or infants with no growth failure/faltering.

Comparison
The comparison group was routine antibiotics following treatment protocols detailed in the 2013 WHO guideline[2].

Outcomes
-Mortality (Dichotomous outcome)
-Clinical deterioration [Dichotomous outcome, defined by the development of any danger signs (obstructed breathing, respiratory distress, cyanosis, shock, severe anemia, convulsion, severe dehydration, profuse watery diarrhea, intractable vomiting, and/or impaired consciousness)]
-Recovery from co-morbidity, (Dichotomous outcome)
-Markers of intestinal inflammation-fecal calprotectin (Continuous outcome)
-Markers of systemic inflammation-serum C reactive protein (Continuous outcome)
-Hospital-acquired infections (Dichotomous outcome)
-Non-response (e.g., not achieving recovery within four months of initiating treatment) (Dichotomous outcome)

All the primary analyses were considered at the longest follow-up. For the outcome of recovery from morbidity, the recovery could be recovery from diarrhea, pneumonia, measles etc.

LITERATURE SEARCH

We conducted systematic electronic queries using key terms in multiple databases, including MEDLINE via PubMed, EMBASE, Web of Science, CINAHL, Scopus, LILACS, WHO Global Index Medicus, and BIOSIS Previews. There were no search restrictions on outcomes, publication year, publication status, or publication language. The search strategies for different databases are available in Appendix 1. The references of formerly published reviews and recently published studies were examined for potential inclusion. We also used The Cochrane Central Register for Controlled Trials and ISRCTN registry to identify studies currently underway. We also searched the websites of pertinent international agencies such as the WHO (including WHO's Reproductive Health Library, electronic Library of Evidence of Nutrition Actions and Global database on the Implementation of Nutrition Action), UNICEF, Global Alliance for Improved Nutrition, International Food Policy Research Institute, International Initiative for Impact Evaluation, Nutrition International, World Bank, USAID and affiliates (e.g., FANTA, SPRING), and the World Food Programme. We searched the abstracts of major conferences, such as annual pediatric academic society meetings. Finally, we used the citation tracking function of the included studies in PubMed to look for any other eligible studies.

DATA EXTRACTION AND SYNTHESIS

For peer review only

Selection of studies

Studies identified during the literature search were collected in an electronic reference manager EndNote[11] literature and duplicated studies were removed. At least two authors screened study titles and abstracts to assess potential eligibility. Studies selected during this initial phase then underwent a full-text review by two authors. The software Covidence[12] was used during the screening process. Disagreements were resolved by discussion and the senior author on the team assisted as needed.

Data extraction

We planned to extract the data for study region/country, study year, study type, intervention exposure (dose, duration, frequency), comparison, outcomes, population characteristics detailed in subgroup analysis, and risk of bias. We also planned to extract the information on feasibility, acceptability, equity, and resource use of the intervention and reported these data in separate tables. We planned to extract raw values for the number of events in the intervention and control group in case of dichotomous outcomes. To avoid reviewer bias, we decided a priori the order of preference for extracting outcomes when data were available in several formats.

Studies with missing data

If a study was only available in an abstract, we contacted the authors for full text. If the full text could not be obtained from any sources, we considered the abstract if sufficient details of study design and outcomes were available. We attempted to find the protocol of each of the potentially included study to assess the details of the methods. If the study protocol was not publicly available, we contacted the authors for the same. If the results of the randomized trial were published in more than one report, we considered all the publications related to that study as one study.

Assessment of risk of bias in included studies

We aimed to evaluate the risk of bias from randomized controlled trials with the Cochrane risk of bias (ROB 2.0)[13]. The risk of bias assessment according to ROB-2 is done for each outcome and not for a particular study[13].

Data Synthesis

We planned to report the review findings both qualitatively and quantitatively. A narrative synthesis was considered to report the characteristics of all included studies and their results. A random-effects meta-analysis was planned when at least two studies possessed sufficient clinical and methodological uniformity for synthesis. The software RevMan was considered for statistical analysis[14]. We planned to assess the dichotomous outcomes using relative risk (RR) effect sizes and continuous outcomes with a mean difference and report with 95 % CIs.

Assessment of heterogeneity

We aimed to analyze statistical heterogeneity in the pooled data using Tau^2, χ^2, and I^2 statistics. We also aimed to assess statistical heterogeneity through visual inspection of forest plots, utilizing the χ^2 test (assessing the p-value) and calculating the Tau^2 and I^2 statistics. We considered it significant statistical heterogeneity when the p-value were less than 0.1, the I^2 value exceeds 50%, and the inspection of forest plots shows substantial variability in the effect of the intervention. Finally, we considered subgroup analysis to identify reasons for eligible statistical heterogeneity.

Assessment of reporting bias

We aimed to assess publication bias of small studies using funnel plots and regression tests for funnel plot asymmetry when the meta-analysis included at least ten studies.

Subgroup analysis and investigation of heterogeneity

We planned the following subgroup analysis however none of them were possible due to lack of studies.

- By different types/definitions of growth failure/faltering
- Age at presentation [newborn (0-28 days), 1-3 months, 4-6 months]
- Gestational age: Preterm birth (< 37 weeks) vs. full-term birth (> 37 weeks)
- Birth weight: Low birth weight (<2500 g) vs. Normal birth weight (> 2500 g)
- HIV exposure: Studies with Participants exposed to HIV VS. Studies with no HIV exposure
- Presentation: Participants with Oedema vs. Patricians with no oedema
- Co-morbidities: With or without co-morbidities
- Nutrition: Babies Breastfeeding or non-breastfed babies
- Location of the treatment: inpatient or outpatient/community
- Dose of antibiotics
- Duration of antibiotics: 7 days vs. > 7 days
- Type of antibiotics

Sensitivity analysis

We planned to complete sensitivity analysis by the use of model for meta-analysis.

Rating of overall quality of evidence

The Grading of Recommendations Assessment, Development, and Evaluation (GRADE) approach was considered to evaluate the overall certainty of evidence using the software GRADEpro[15]. The GRADE approach is a comprehensive framework used to assess the overall certainty of the evidence for an outcome utilizing study characteristics such as study design, inconsistency, indirectness of evidence, risk of bias, publication bias, and imprecision estimates.

RESULTS

Literature search

We screened 5137 titles and abstracts; 157 eligible studies were screened for full text reviews. None of the available studies qualified for inclusion in this review. Figure 1 shows the results of the literature search. The reasons for exclusions are available in table of excluded studies (supplementary document 1). In summary, 85 studies were excluded because of wrong study design, 55 studies were excluded due to ineligible patient population, 7 studies were excluded because of the ineligible comparison group, 8 studies were excluded because of ineligible intervention, 2 studies were excluded because of wrong indication.

DISCUSSION

This systematic review aimed to assess the effect of routine use of antibiotics for treatment of malnutrition in infants less than 6 months of age. Even though we reviewed more than 4000 titles and about 157 full text studies, none of the studies qualified for inclusion in this review.

We followed the methodology of the Cochrane Collaboration to conduct this systematic review. A detailed protocol was prepared prior to commencing the review process and was both externally

For peer review only

reviewed and registered publicly on PROSPERO's international database of prospective systematic reviews. The title and abstracts of the studies were screened independently by two study authors. We planned to study risk of bias using the revised Cochrane risk of bias 2.0 tool for each outcome within a study rather than determining the risk of bias based on all outcomes for a particular study. However, no eligible study was found to answer the clinical question in this review.

We noted two studies that were of great interest to our population of interest[16,17]. These studies were excluded because of lack of a comparison group that was treated with antibiotics according to current guideline of World health Organization for treatment of wasting.[2] Both the studies included participants that fall in the age range (i.e. < 6 months) considered for this review however they also included participants beyond six months of age[16,17]. Both studies were randomized double-blind placebo-controlled trials. One study was conducted in Kenya[16] and another study was a multicounty trial[17]. The study from Kenya [16] used co-trimoxazole in the community settings for prevention of mortality in severely malnourished children 2-59 months of age after they were treated according to WHO protocol (both the groups received antibiotics according to WHO protocol during the stabilization period). Daily Co-trimoxazole after initial treatment for severe malnutrition did not lead to prevention of mortality in children 2-59 months of age (Hazard ratio 0·90, 95% CI 0·71-1·16). The results for infants 2 months to 5 months of age were similar to overall results[16]. The other study used biannual azithromycin in children 1-59 months of age irrespective of nutritional status[17] and showed a 13.5 % reduction in mortality in the azithromycin group compared to placebo. A subgroup analysis for malnourished children showed similar results[18]. There were two other studies that could have been included; however, both of these studies included both nourished and malnourished participants and we could not obtain the randomized data for our population of interest[19,20]. We also identified a recently completed trial[21] and requested the data for inclusion to this review, however study investigators were in process of submission of their results to a peer review journal and are not yet willing to share those data.

Implications for research

Future randomized studies, with adequate sample size of infants under the age of 6 months with malnutrition and/or growth faltering are needed to confirm therapeutic effect of antibiotic treatment observed in children 6-59 months of age[2]. Additional data are also required to assess the appropriate antibiotic, dose, route, frequency and duration of antibiotic treatment. Safety profile in terms of acute reactions such as allergic reaction, gastrointestinal disturbance etc. and long-term effects such as effects on gut microbiome and antibiotic resistance also need further investigation.

CONCLUSIONS

There is paucity of evidence to assess the effect of routine use of antibiotics in infants less than 6 months of age with malnutrition. Future studies with large sample sizes are needed to evaluate the potential risks and benefits from the use of antibiotics in malnourished children less than 6 months of age.

For peer review only

Amendments
We conducted literature searches, screening of titles, selection of studies, data extraction, and analysis according to the plan outlined in the protocol.

Ethics statements
Patient consent for publication
This study does not involve human participants and ethical approval was not required

Patient and public involvement
Patients and/or the public were not involved in the design, or conduct, or reporting, or dissemination plans of this research.

Data Sharing Statement
We provide the list of excluded studies and our search strategies. The results of literature search are available on request.

Funding
This work is funded by World Health Organization grant no 202725572. WHO also provided technical support for this work.

Acknowledgements
We are very thankful to Allison Daniel, Jaden Bendabenda and Zita Weise Prinzo, Kirrily De Polnay for their input to improve this review.

Competing Interest
There are no conflicts of interests identified in the conduction of this research.

Author Contributions: Conceptualization, A.I..; methodology, A.I., F.C.; validation, A.I., F.C., M.F., M.S..; formal analysis, A.I., F.C.; resources, E.TS., A.S., O.T; data curation, F.C., M.F., M.S.; writing, A.I., F.C.; review and editing, A.I., F.C., J.D., Z.B.. All authors have read and agreed to the published version of the manuscript.

References

1. United Nations Children's Fund (UNICEF), World Health Organization, International Bank for Reconstruction and Development/The World Bank. Levels and trends in child malnutrition: Key Findings of the 2020 Edition of the Joint Child Malnutrition Estimates. Geneva: World Health Organization. Licence: CC BY-NC-SA 3.0 IGO. 2020.
2. WHO. Guideline: Updates on the management of severe acute malnutrition in infants and children. Geneva: World Health Organization. 2013.
3. Kerac M, Blencowe H, Grijalva-Eternod C, et al. Prevalence of wasting among under 6-month-old infants in developing countries and implications of new case definitions using WHO growth standards: a secondary data analysis. *Arch Dis Child.* 2011;96(11):1008-1013.

For peer review only

4. Patwari AK, Kumar S, Beard J. Undernutrition among infants less than 6 months of age: an underestimated public health problem in India. *Matern Child Nutr.* 2015;11(1):119-126.
5. Alcoba G, Kerac M, Breysse S, et al. Do children with uncomplicated severe acute malnutrition need antibiotics? A systematic review and meta-analysis. *PLoS One.* 2013;8(1):e53184.
6. Stam J, van Stuijvenberg M, Gruber C, et al. Antibiotic use in infants in the first year of life in five European countries. *Acta Paediatr.* 2012;101(9):929-934.
7. Droste JH, Wieringa MH, Weyler JJ, Nelen VJ, Vermeire PA, Van Bever HP. Does the use of antibiotics in early childhood increase the risk of asthma and allergic disease? *Clin Exp Allergy.* 2000;30(11):1547-1553.
8. Trasande L, Blustein J, Liu M, Corwin E, Cox LM, Blaser MJ. Infant antibiotic exposures and early-life body mass. *Int J Obes (Lond).* 2013;37(1):16-23.
9. Higgins JPT TJ, Chandler J, Cumpston M, Li T, Page MJ, Welch VA (editors). Cochrane Handbook for Systematic Reviews of Interventions version 6.2 Available from www.training.cochrane.org/handbook. 2021.
10. Page MJ, McKenzie JE, Bossuyt PM, et al. The PRISMA 2020 statement: an updated guideline for reporting systematic reviews. *BMJ.* 2021;372:n71.
11. *EndNote* [computer program]. Version EndNote X9. Philadelphia, PA: Clarivate; 2013.
12. *Covidence systematic review software* [computer program]. Melbourne, Australia: www.covidence.org; 2021.
13. Sterne JAC, Savovic J, Page MJ, et al. RoB 2: a revised tool for assessing risk of bias in randomised trials. *BMJ.* 2019;366:l4898.
14. *Review Manager (RevMan)* [computer program]. Version 5.3. Copenhagen: The Nordic Cochrane Center TCC; 2014.
15. *GRADEpro GDT: GRADEpro guideline development tool* [computer program]. McMaster University: Evidence Prime, Inc; 2020.
16. Berkley JA, Ngari M, Thitiri J, et al. Daily co-trimoxazole prophylaxis to prevent mortality in children with complicated severe acute malnutrition: a multicentre, double-blind, randomised placebo-controlled trial. *Lancet Glob Health.* 2016;4(7):e464-473.
17. Keenan JD, Bailey RL, West SK, et al. Azithromycin to Reduce Childhood Mortality in Sub-Saharan Africa. *N Engl J Med.* 2018;378(17):1583-1592.
18. O'Brien KS, Arzika AM, Maliki R, et al. Biannual azithromycin distribution and child mortality among malnourished children: A subgroup analysis of the MORDOR cluster-randomized trial in Niger. *PLoS Med.* 2020;17(9):e1003285.
19. Gore-Langton GR, Cairns M, Compaore YD, et al. Effect of adding azithromycin to the antimalarials used for seasonal malaria chemoprevention on the nutritional status of African children. *Trop Med Int Health.* 2020;25(6):740-750.
20. Pavlinac PB, Singa BO, Tickell KD, et al. Azithromycin for the prevention of rehospitalisation and death among Kenyan children being discharged from hospital: a double-blind, placebo-controlled, randomised controlled trial. *Lancet Glob Health.* 2021;9(11):e1569-e1578.
21. Muhammad A SY, Nisar MI, Baloch B, Yazdani AT, Yazdani N, Jehan F. . Nutritional support for lactating women with or without azithromycin for infants compared to

For peer review only

breastfeeding counseling alone in improving the 6-month growth outcomes among infants of peri-urban slums in Karachi, Pakistan-the protocol for a multiarm assessor-blinded randomized controlled trial *Trials.* 2020;1(21):756.

For peer review only

Figure 1: PRISMA flow diagram

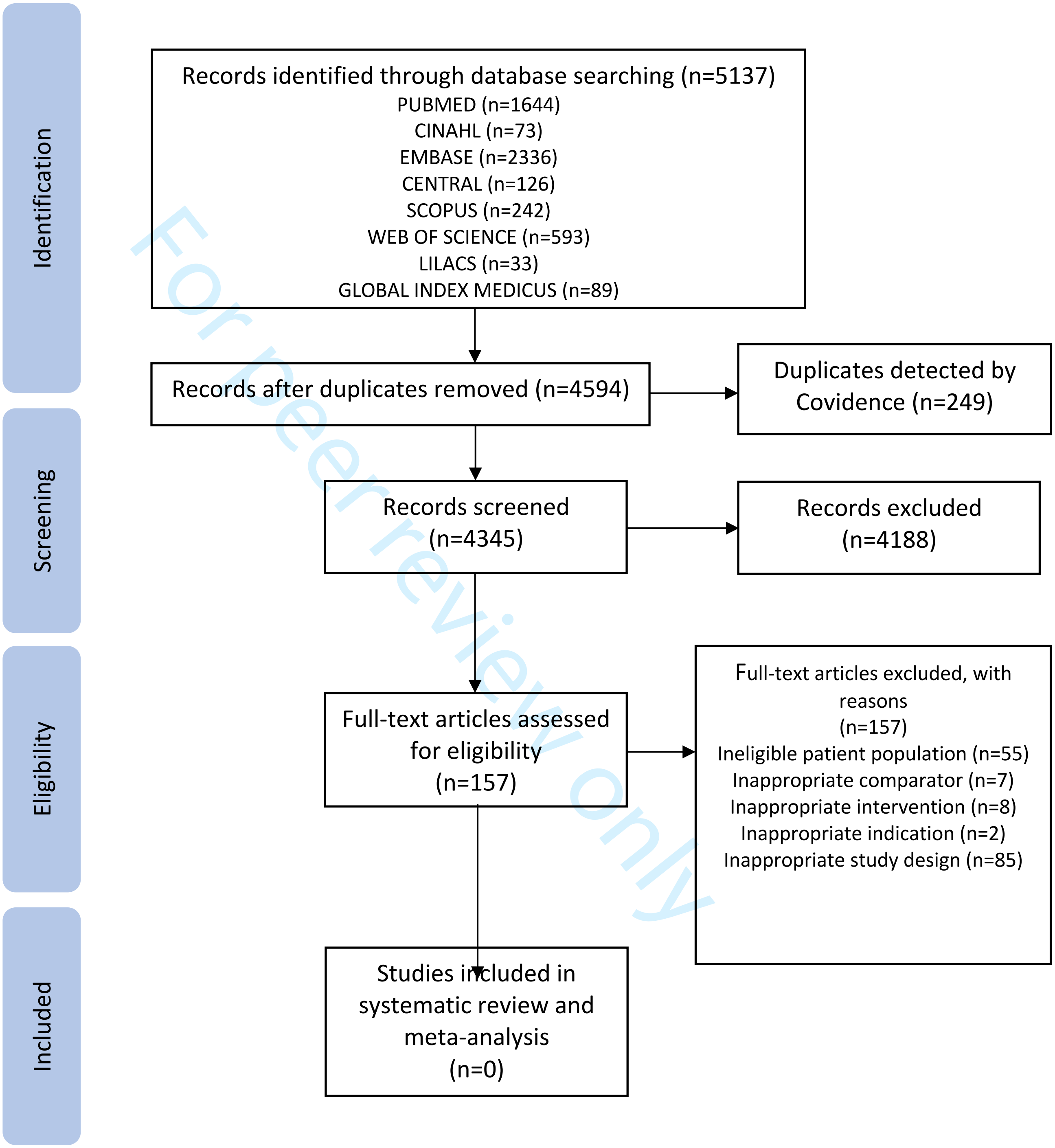

Appendix 1
Search Strategies for Growth Failure AND Antibiotics
PubMed

(("Infant"[MeSH Terms] OR "Child"[MeSH Terms:noexp] OR "Premature Birth"[MeSH Terms] OR ("infant*"[Title/Abstract] OR "infancy"[Title/Abstract] OR "baby"[Title/Abstract] OR "babies"[Title/Abstract] OR "neonat*"[Title/Abstract] OR "neo nat*"[Title/Abstract] OR "newborn*"[Title/Abstract] OR "new born*"[Title/Abstract] OR "newly born*"[Title/Abstract] OR "preterm"[Title/Abstract] OR "preterms"[Title/Abstract] OR "pre term"[Title/Abstract] OR "pre terms"[Title/Abstract] OR "lbw"[Title/Abstract] OR "vlbw"[Title/Abstract] OR "elbw"[Title/Abstract] OR "child*"[Title/Abstract] OR "youth"[Title/Abstract] OR "juvenile*"[Title/Abstract]) OR ("premature*"[Title/Abstract] AND ("birth*"[Title/Abstract] OR "born"[Title/Abstract] OR "deliver*"[Title/Abstract])) OR ("low"[Title/Abstract] AND ("birthweight*"[Title/Abstract] OR "birth weight*"[Title/Abstract]))) AND ("Malnutrition"[MeSH Terms] OR "Failure to Thrive"[MeSH Terms] OR "Growth Disorders"[MeSH Terms:noexp] OR "Severe Acute Malnutrition"[MeSH Terms] OR "Wasting Syndrome"[MeSH Terms:noexp] OR "Infant Nutrition Disorders"[MeSH Terms:noexp] OR ("growth failure*"[Title/Abstract] OR "growth falter*"[Title/Abstract] OR "slow growth"[Title/Abstract] OR "slowed growth"[Title/Abstract] OR "Malnutrition"[Title/Abstract] OR "malnourish*"[Title/Abstract] OR "Severe Acute Malnutrition"[Title/Abstract] OR "SAM"[Title/Abstract] OR "wasting"[Title/Abstract] OR "wasted"[Title/Abstract] OR "growth restrict*"[Title/Abstract] OR "growth retard*"[Title/Abstract] OR "Failure to Thrive"[Title/Abstract] OR "FTT"[Title/Abstract] OR "growth disorder*"[Title/Abstract] OR "growth arrest*"[Title/Abstract] OR "growth deficien*"[Title/Abstract] OR "growth disturb*"[Title/Abstract])) AND ("Anti-Bacterial Agents"[Pharmacological Action] OR "Anti-Bacterial Agents"[MeSH Terms] OR ("antibiotic*"[Title/Abstract] OR "anti bacterial agent*"[Title/Abstract] OR "antibacterial*"[Title/Abstract] OR "anti bacterial*"[Title/Abstract] OR "bactericid*"[Title/Abstract] OR "bacteriocid*"[Title/Abstract] OR "anti mycobacterial*"[Title/Abstract] OR "antimycobacterial*"[Title/Abstract]))) NOT ("Animals"[MeSH Terms] NOT ("Animals"[MeSH Terms] AND "Humans"[MeSH Terms]))

CINAHL

S1 (MH "Infant+")
S2 (MH "Child")
S3 (MH "Childbirth, Premature")
S4 TI (infant* OR infancy OR baby OR babies OR neonat* OR "neo nat*" OR newborn* OR "new born*" OR "newly born*" OR preterm OR preterms OR "pre term" OR "pre terms" OR lbw OR vlbw OR elbw OR child* OR youth OR juvenile*)
S5 AB (infant* OR infancy OR baby OR babies OR neonat* OR "neo nat*" OR newborn* OR "new born*" OR "newly born*" OR preterm OR preterms OR "pre term" OR "pre terms" OR lbw OR vlbw OR elbw OR child* OR youth OR juvenile*)
S6 TI (premature* N2 (birth* OR born OR deliver*))
S7 AB (premature* N2 (birth* OR born OR deliver*))
S8 TI (low N2 (birthweight* OR "birth weight*"))
S9 AB (low N2 (birthweight* OR "birth weight*"))
S10 S1 OR S2 OR S3 OR S4 OR S5 OR S6 OR S7 OR S8 OR S9

S11 (MH "Malnutrition")
S12 (MH "Failure to Thrive")
S13 (MH "Growth Disorders")
S14 (MH "Wasting Syndrome")
S15 (MH "Infant Nutrition Disorders")
S16 TI ((grow*) N2 (fail* OR falter* OR slow* OR restrict* OR retard* OR deficien* OR arrest* OR disturb* OR disorder*))
S17 AB ((grow*) N2 (fail* OR falter* OR slow* OR restrict* OR retard* OR deficien* OR arrest* OR disturb* OR disorder*))
S18 TI ((fail*) W2 (thrive))
S19 AB ((fail*) W2 (thrive))
S20 TI (Malnutrition OR malnourish* OR "Severe Acute Malnutrition" OR SAM OR wasting OR wasted OR FTT)
S21 AB (Malnutrition OR malnourish* OR "Severe Acute Malnutrition" OR SAM OR wasting OR wasted OR FTT)
S22 S11 OR S12 OR S13 OR S14 OR S15 OR S16 OR S17 OR S18 OR S19 OR S20 OR S21
S23 (MH "Antibiotics+")
S24 TI (antibiotic* OR "anti bacterial*" OR "antibacterial*" OR bactericid*OR bacteriocid* OR "anti mycobacterial*" OR "antimycobacterial*")
S25 AB (antibiotic* OR "anti bacterial*" OR "antibacterial*" OR bactericid* OR bacteriocid* OR "anti mycobacterial*" OR "antimycobacterial*")
S26 S23 OR S24 OR S25
S27 (MH "Animals") NOT ((MH "Animals") AND (MH "Human"))
S28 S10 AND S22 AND S26
S29 S28 NOT S27
S30 S28 NOT S27 Exclude Medline Records

Embase

#1'infant'/exp OR 'newborn'/exp OR 'prematurity'/exp OR 'low birth weight'/exp OR 'very low birth weight'/exp OR 'extremely low birth weight'/exp OR 'juvenile'/de OR 'child'/de
#2 infant*:ti,ab OR infancy:ti,ab OR baby:ti,ab OR babies:ti,ab OR neonat*:ti,ab OR 'neo nat*':ti,ab OR newborn*:ti,ab OR 'new born*':ti,ab OR 'newly born*':ti,ab OR preterm:ti,ab OR preterms:ti,ab OR 'pre term':ti,ab OR 'pre terms':ti,ab OR lbw:ti,ab OR vlbw:ti,ab OR elbw:ti,ab OR child*:ti,ab OR youth:ti,ab OR juvenile*:ti,ab
#3 (premature* NEAR/2 (birth* OR born OR deliver*)):ti,ab
#4 (low NEAR/2 (birthweight* OR 'birth weight*')):ti,ab
#5 #1 OR #2 OR #3 OR #4

#6 'malnutrition'/exp OR 'failure to thrive'/exp OR 'growth disorder'/de OR 'wasting syndrome'/de OR 'growth retardation'/de
#7(grow* NEAR/2 (fail* OR falter* OR slow* OR restrict* OR retard* OR deficien* OR arrest* OR disturb* OR disorder*)):ti,ab
#8 (fail* NEXT/2 thrive):ti,ab
#9 malnutrition:ti,ab OR malnourish*:ti,ab OR 'severe acute malnutrition':ti,ab OR sam:ti,ab OR

For peer review only

wasting:ti,ab OR wasted:ti,ab OR ftt:ti,ab
#10 #6 OR #7 OR #8 OR #9
#11'antibiotic agent'/exp OR 'bactericide'/de OR 'antimycobacterial agent'/de
#12 antibiotic*:ti,ab OR 'anti bacterial*':ti,ab OR 'antibacterial*':ti,ab OR bactericid*:ti,ab OR
bacteriocid*:ti,ab OR antimycobacterial*:ti,ab OR 'anti mycobacterial*':ti,ab
#13 #11 OR #12
#14 #5 AND #10 AND #13
#15 #14 NOT ('animals'/exp NOT 'humans'/exp)
#16 #15 NOT [medline]/lim
CENTRAL
#1 MeSH descriptor: [Infant] explode all trees
#2 MeSH descriptor: [Child] this term only
#3 MeSH descriptor: [Premature Birth] explode all trees
#4 infant*:ti,ab OR infancy:ti,ab OR baby:ti,ab OR babies:ti,ab OR neonat*:ti,ab OR newborn*:ti,ab
OR preterm:ti,ab OR preterms:ti,ab OR lbw:ti,ab OR vlbw:ti,ab OR elbw:ti,ab OR child*:ti,ab OR
youth:ti,ab OR juvenile*:ti,ab
#5 (neo NEXT nat* OR new NEXT born* OR newly NEXT born* OR pre NEXT term OR pre NEXT
terms):ti,ab
#6 (premature* NEAR/2 (birth* OR born OR deliver*)):ti,ab
#7 (low NEAR/2 (birthweight* OR birth NEXT weight*)):ti,ab
#8 #1 OR #2 OR #3 OR #4 OR #5 OR #6 OR #7
#9 MeSH descriptor: [Malnutrition] explode all trees
#10 MeSH descriptor: [Failure to Thrive] explode all trees
#11 MeSH descriptor: [Growth Disorders] this term only
#12 MeSH descriptor: [Severe Acute Malnutrition] explode all trees
#13 MeSH descriptor: [Wasting Syndrome] this term only
#14 MeSH descriptor: [Infant Nutrition Disorders] this term only
#15 ((grow*) NEAR/2 (fail* OR falter* OR slow* OR restrict* OR retard* OR deficien* OR arrest* OR
disturb* OR disorder*)):ti,ab

#16 ((fail*) NEXT/2 (thrive)):ti,ab
#17 (severe NEXT acute NEXT malnutrition):ti,ab
#18 malnutrition:ti,ab OR malnourish*:ti,ab OR sam:ti,ab OR wasting:ti,ab OR wasted:ti,ab OR
ftt:ti,ab
#19 #9 OR #10 OR #11 OR #12 OR #13 OR #14 OR #15 OR #16 OR #17 OR #18
#20 MeSH descriptor: [Anti-Bacterial Agents] explode all trees
#21 antibiotic*:ti,ab OR antibacterial*:ti,ab OR bactericid*:ti,ab OR bacteriocid*:ti,ab OR
antimycobacterial*:ti,ab
#22 (anti NEXT bacterial*):ti,ab
#23 (anti NEXT mycobacterial*):ti,ab
#24 #20 OR #21 OR #22 OR #23
#25 #8 AND #19 AND #24
#26 MeSH descriptor: [Animals] explode all trees
#27 MeSH descriptor: [Humans] explode all trees
#28 (#26 NOT (#26 AND #27))
#29 #25 NOT #28
#30 "accession number" NEAR pubmed
#31 #29 NOT #30
Scopus
((TITLE-ABS (infant* OR infancy OR baby OR babies OR neonat* OR "neo nat*" OR
newborn* OR "new born*" OR "newly born*" OR preterm OR preterms OR "pre term" OR
"pre terms" OR lbw OR vlbw OR elbw OR child* OR youth OR juvenile*)) OR (TITLE-ABS
(low W/2 (birthweight* OR "birth weight*"))) OR (TITLE-ABS (premature* W/2 (birth* OR
born OR deliver*)))) AND ((TITLE-ABS ((grow*) W/2 (fail* OR falter* OR slow* OR
restrict* OR retard* OR deficien* OR arrest* OR disturb* OR disorder*))) OR (TITLE-ABS (
(fail*) PRE/2 (thrive))) OR (TITLE-ABS (malnutrition OR malnourish* OR "Severe Acute
Malnutrition" OR sam OR wasting OR wasted OR ftt))) AND (TITLE-ABS (antibiotic* OR
"anti bacterial*" OR "antibacterial*" OR bactericid* OR bacteriocid* OR "anti mycobacterial*"
OR "antimycobacterial*")) AND NOT INDEX (medline)
Web of Science
#1 TI=(infant* OR infancy OR baby OR babies OR neonat* OR "neo nat*" OR newborn* OR
"new born*" OR "newly born*" OR preterm OR preterms OR "pre term" OR "pre terms" OR
lbw OR vlbw OR elbw OR child* OR youth OR juvenile*)
#2 AB=(infant* OR infancy OR baby OR babies OR neonat* OR "neo nat*" OR newborn* OR
"new born*" OR "newly born*" OR preterm OR preterms OR "pre term" OR "pre terms" OR
lbw OR vlbw OR elbw OR child* OR youth OR juvenile*)
#3 TI=(premature* NEAR/2 (birth* OR born OR deliver*))

#4 AB=(premature* NEAR/2 (birth* OR born OR deliver*))
#5 TI=(low NEAR/2 (birthweight* OR "birth weight*"))
#6 AB=(low NEAR/2 (birthweight* OR "birth weight*"))
#7 #6 OR #5 OR #4 OR #3 OR #2 OR #1
#8 TI=((grow*) NEAR/2 (fail* OR falter* OR slow* OR restrict* OR retard* OR deficien* OR arrest*
OR disturb* OR disorder*))
#9 AB=((grow*) NEAR/2 (fail* OR falter* OR slow* OR restrict* OR retard* OR deficien* OR arrest*
OR disturb* OR disorder*))
#10 TI=((fail*) NEAR/2 (thrive))
#11 AB=((fail*) NEAR/2 (thrive))
#12 TI =(Malnutrition OR malnourish* OR "Severe Acute Malnutrition" OR SAM OR wasting OR
wasted OR FTT)
#13 AB=(Malnutrition OR malnourish* OR "Severe Acute Malnutrition" OR SAM OR wasting OR
wasted OR FTT)
#14 #13 OR #12 OR #11 OR #10 OR #9 OR #8
#15 TI=(antibiotic* OR "anti bacterial*" OR "antibacterial*" OR bactericid* OR bacteriocid* OR "anti
mycobacterial*" OR "antimycobacterial*")
#16 AB=(antibiotic* OR "anti bacterial*" OR "antibacterial*" OR bactericid* OR bacteriocid* OR
"anti mycobacterial*" OR "antimycobacterial*")
#17 #16 OR #15

For peer review only

#18 #17 AND #14 AND #7
LILACS
(mh:(infant)) OR ((mh:(child))) OR ((mh:("Premature Birth"))) OR ((ti:((premature* AND (birth* OR born OR deliver*))))) OR ((ab:((premature* AND (birth* OR born OR deliver*))))) OR ((ti:((low AND (birthweight* OR "birth weight*"))))) OR ((ab:((low AND (birthweight* OR "birth weight*"))))) OR ((ti:(infant* OR infancy OR baby OR babies OR neonat* OR "neo nat*" OR newborn* OR "new born*" OR "newly born*" OR preterm OR preterms OR "pre term" OR "pre terms" OR lbw OR vlbw OR elbw OR child* OR youth OR juvenile*))) OR ((ab:(infant* OR infancy OR baby OR babies OR neonat* OR "neo nat*" OR newborn* OR "new born*" OR "newly born*" OR preterm OR preterms OR "pre term" OR "pre terms" OR lbw OR vlbw OR elbw OR child* OR youth OR juvenile*))) AND ((mh:(malnutrition)) OR ((mh:("Failure to Thrive"))) OR ((mh:("Growth Disorders"))) OR ((mh:("Severe Acute Malnutrition"))) OR ((mh:("Wasting Syndrome"))) OR ((mh:("Infant Nutrition Disorders"))) OR ((ti:("growth failure*" OR "growth falter*" OR "slow growth" OR "slowed growth" OR malnutrition OR malnourish* OR "Severe Acute Malnutrition" OR sam OR wasting OR wasted OR "growth restrict*" OR "growth retard*" OR "Failure to Thrive" OR ftt OR "growth disorder*" OR "growth arrest*" OR "growth deficien*" OR "growth disturb*"))) OR ((ab:("growth failure*" OR "growth falter*" OR "slow growth" OR "slowed growth" OR malnutrition OR malnourish* OR "Severe Acute Malnutrition" OR sam OR wasting OR wasted OR "growth restrict*" OR "growth retard*" OR "Failure to Thrive" OR ftt OR "growth disorder*" OR "growth arrest*" OR "growth deficien*" OR "growth disturb*")))) AND ((mh:("Anti-Bacterial Agents")) OR ((ti:(antibiotic* OR "anti bacterial agent*" OR antibacterial* OR "anti bacterial*" OR "bactericid*" OR "bacteriocid*"

OR "anti mycobacterial*" OR antimycobacterial*))) OR ((ab:(antibiotic* OR "anti bacterial agent*" OR antibacterial* OR "anti bacterial*" OR "bactericid*" OR "bacteriocid*" OR "anti mycobacterial*" OR antimycobacterial*)))) AND NOT ((mh:(animals)) AND NOT ((mh:(animals)) AND ((mh:(humans)))))

Global Index Medicus
(mh:(infant)) OR ((mh:(child))) OR ((mh:("Premature Birth"))) OR ((ti:((premature* AND (birth* OR born OR deliver*))))) OR ((ab:((premature* AND (birth* OR born OR deliver*))))) OR ((ti:((low AND (birthweight* OR "birth weight*"))))) OR ((ab:((low AND (birthweight* OR "birth weight*"))))) OR ((ti:(infant* OR infancy OR baby OR babies OR neonat* OR "neo nat*" OR newborn* OR "new born*" OR "newly born*" OR preterm OR preterms OR "pre term" OR "pre terms" OR lbw OR vlbw OR elbw OR child* OR youth OR juvenile*))) OR ((ab:(infant* OR infancy OR baby OR babies OR neonat* OR "neo nat*" OR newborn* OR "new born*" OR "newly born*" OR preterm OR preterms OR "pre term" OR "pre terms" OR lbw OR vlbw OR elbw OR child* OR youth OR juvenile*))) AND ((mh:(malnutrition)) OR ((mh:("Failure to Thrive"))) OR ((mh:("Growth Disorders"))) OR ((mh:("Severe Acute Malnutrition"))) OR ((mh:("Wasting Syndrome"))) OR ((mh:("Infant Nutrition Disorders"))) OR ((ti:("growth failure*" OR "growth falter*" OR "slow growth" OR "slowed growth" OR malnutrition OR malnourish* OR "Severe Acute Malnutrition" OR sam OR wasting OR wasted OR "growth restrict*" OR "growth retard*" OR "Failure to Thrive" OR ftt OR "growth disorder*" OR "growth arrest*" OR "growth deficien*" OR "growth disturb*"))) OR ((ab:("growth failure*" OR "growth falter*" OR "slow growth" OR "slowed growth" OR malnutrition OR malnourish* OR "Severe Acute Malnutrition" OR sam OR wasting OR wasted OR "growth restrict*" OR "growth retard*" OR "Failure to Thrive" OR ftt OR "growth disorder*" OR "growth arrest*" OR "growth deficien*" OR "growth disturb*")))) AND ((mh:("Anti-Bacterial Agents")) OR ((ti:(antibiotic* OR "anti bacterial agent*" OR antibacterial* OR "anti bacterial*" OR "bactericid*" OR "bacteriocid*" OR "anti mycobacterial*" OR antimycobacterial*))) OR ((ab:(antibiotic* OR "anti bacterial agent*" OR antibacterial* OR "anti bacterial*" OR "bactericid*" OR "bacteriocid*" OR "anti mycobacterial*" OR antimycobacterial*)))) AND NOT ((mh:(animals)) AND NOT ((mh:(animals)) AND ((mh:(humans))))) AND (collection_gim:("IMSEAR" OR "WPRIM" OR "IMEMR" OR "AIM"))

BIOSIS Previews:

S1 TI (infant* OR infancy OR baby OR babies OR neonat* OR "neo nat*" OR newborn* OR "new born*" OR "newly born*" OR preterm OR preterms OR "pre term" OR "pre terms" OR lbw OR vlbw OR elbw OR child* OR youth OR juvenile*)
S2 AB (infant* OR infancy OR baby OR babies OR neonat* OR "neo nat*" OR newborn* OR "new born*" OR "newly born*" OR preterm OR preterms OR "pre term" OR "pre terms" OR lbw OR vlbw OR elbw OR child* OR youth OR juvenile*)
S3 TI (premature* N2 (birth* OR born OR deliver*))
S4 AB (premature* N2 (birth* OR born OR deliver*))
S5 TI (low N2 (birthweight* OR "birth weight*"))
S6 AB (low N2 (birthweight* OR "birth weight*"))
S7 S1 OR S2 OR S3 OR S4 OR S5 OR S6
S8 TI ((grow*) N2 (fail* OR falter* OR slow* OR restrict* OR retard* OR deficien* OR arrest* OR disturb* OR disorder*))
S9 AB ((grow*) N2 (fail* OR falter* OR slow* OR restrict* OR retard* OR deficien* OR arrest* OR disturb* OR disorder*))
S10 TI ((fail*) W2 (thrive))
S11 AB ((fail*) W2 (thrive))
S12 TI (Malnutrition OR malnourish* OR "Severe Acute Malnutrition" OR SAM OR wasting OR wasted OR FTT)
S13 AB (Malnutrition OR malnourish* OR "Severe Acute Malnutrition" OR SAM OR wasting OR wasted OR FTT)
S14 S8 OR S9 OR S10 OR S11 OR S12 OR S13
S15 TI (antibiotic* OR "anti bacterial*" OR antibacterial* OR bactericid* OR bacteriocid* OR "anti mycobacterial*" OR antimycobacterial*)
S16 AB (antibiotic* OR "anti bacterial*" OR antibacterial* OR bactericid* OR bacteriocid* OR "anti mycobacterial*" OR antimycobacterial*)
S17 S15 OR S16
S18 S7 AND S14 AND S17

For peer review only

Antibiotic as an adjunct to treatment of severe acute malnutrition in infants less than six months of age. A protocol for the systematic review

List of Exclusions and Reasons for Exclusions.

Abate 2020[1]	Inappropriate study design and appropriate control was not available
Adam 1954 [2]	Inappropriate Study Design
Adem 2020[3]	Inappropriate Patient Population. Study was a cohort study and only included children 6-59 months of age
Ahmed 1999[4]	Inappropriate Patient Population. Even though all the children were severely malnourished, all of them had diarrhea. Also, the study was not a randomized study.
Ahmed 2001 [5]	Inappropriate Study Design. Authors discuss a protocol for treatment of severe acute malnutrition. The study was a descriptive review
Akbani 1977[6]	Inappropriate study population. Children with SAM who were infected with tuberculosis.
AmorelliGonzaga 1989[7]	Inappropriate study population. Study investigated miomycin and included both well-nourished and malnourished children.
Amza 2013[8]	Inappropriate study population. Study investigated mass azithromycin distribution and included children irrespective of the nutritional status.
Amza 2014[9]	Inappropriate study population. Study investigated mass azithromycin distribution and included children irrespective of the nutritional status.
Angelakis 2014[10]	Inappropriate study population. Study included patients with Q fever endocarditis patients treated with doxycycline and hydroxychloroquine
Ashorn 2018[11]	Inappropriate study population. Study included children with diarrhea, dysentery and malnutrition.
Ashraf 2012[12]	Inappropriate Comparator. Children aged 2-59 months having severe pneumonia with SAM were randomized to day-care or hospital-care. All the children received antibiotics.
Autret 1989[13]	Inappropriate study Design. Study to assess the pharmacokinetics of gentamicin.
Ayelign 2018[14]	Inappropriate study population. Children with urinary tract infections.
Ayiyi 1987[15]	Inappropriate study Design. The study was a systematic review
Aziz 2015[16]	Inappropriate Intervention. Study investigated vitamin K supplementation.
Berkley 2009 [17]	Wrong comparator
Berkley 2016[18]	Inappropriate Comparator. Both the intervention and comparison group were treated according to the WHO

Antibiotic as an adjunct to treatment of severe acute malnutrition in infants less than six months of age. A protocol for the systematic review

List of Exclusions and Reasons for Exclusions.

	protocol and then randomized to Co-trimoxazole vs. placebo.
Berkowitz 1992[19]	Inappropriate Study Design. Review article.
Bhatnagar 1992[20]	Inappropriate patient population. All the patients had diarrhea. All of them were given gentamicin
Bhatnagar 1996[21]	Inappropriate Study Design. Observation study in children with persistent diarrhea
Birindwa 2020[22]	Inappropriate Study Design. Observational study in children with severe pneumonia
Bleakly 2014[23]	Inappropriate Study Design. Before and after study to assess the effectiveness of a protocol to treat SAM.
Boesen 1953[24]	Inappropriate Indication. Antibiotics were given for treatment of gastroesophageal reflux.
Bolme 1980[25]	Inappropriate Study Design: Pharmacokinetic study for Penicillin in children
Bolme 1988[26]	Inappropriate Study Design: Pharmacokinetic study for streptomycin in children with Tuberculosis.
Bolme 1995[27]	Inappropriate Study Design. Pharmacokinetic study in malnourished children for Penicillin.
Bravo 1982[28]	Inappropriate study design: Study to assess the pharmacokinetics
Bravo 1984[29]	Inappropriate study design: Study to assess the pharmacokinetics
Bredow 1994[30]	Inappropriate Study Design. Observational study
Brodwall 2016[31]	Inappropriate Study Design. Letter to editor
Bruhn 2016[32]	Inappropriate Study Design. Observational study
Brunozi 2019[33]	Inappropriate Study Design. Observational study
Buchanan 1977[34]	Inappropriate Study Design. Letter to editor
Buchanan 1978[35]	Inappropriate Study Design. Observational study
Buchanan 1979 [36]	Inappropriate Study Design. Observational study
Bunn 2009[37]	Inappropriate Study Design. Commentary
Cakir 2012[38]	Inappropriate Patient Population. Children studied leukemia
Caksen 2000[39]	Inappropriate Study Design. Observational study
Caksen 2000[40]	Inappropriate Study Design. Observational study
Cedrato 1974[41]	Inappropriate Study Design. Observational study
Chisti 2015[42]	Inappropriate Patient Population. Children with respiratory symptoms only
Church 2015[43]	Inappropriate Study Design. Observational study
Cochlovius 1953[44]	Inappropriate intervention. Observational study
Dicko 2016[45]	Inappropriate study population. Intervention was given to all the children irrespective of nutritional status at the time of chemoprophylaxis for malaria.
Dieng 2014[46]	Inappropriate Study Design. Observational study
Dubray 2008[47]	Inappropriate study population. Both the study groups received antibiotics and these were given to children 6-59 months of age
Duffau 1990[48]	Inappropriate study population. Children with diarrhea

For peer review only

Antibiotic as an adjunct to treatment of severe acute malnutrition in infants less than six months of age. A protocol for the systematic review

List of Exclusions and Reasons for Exclusions.

Dutta 2006[49]	Inappropriate study design: Observational study
Eriksson 1983[50]	Inappropriate Patient Population. Also, observational study
Eriksson 1988[51]	Inappropriate Patient Population. Also, observational study
Ford 1976[52]	Inappropriate Study Design. Observational study
Friedrich 2016[53]	Inappropriate Study Design. Commentary
Garcia 1974[54]	Inappropriate Study Design. Observational study
Gardner 2011[55]	Inappropriate Study Design. Commentary
Gennaro 2017[56]	Inappropriate Study Design. Commentary
Gensch 1957[57]	Inappropriate Study Design. Observational study
Gernaat 1998[58]	Inappropriate Study Design. Observational study
Ghosh 1995[59]	Inappropriate Patient Population. Children with persistent diarrhea
Girma 2018[60]	Inappropriate Study Design. Observational study
Gore Langton 2020[61]	Inappropriate Patient Population. The antibiotics were given irrespective of nutritional status and study included children beyond six months of age. The comparison group was not appropriate.
Hecht 2015 [62]	Inappropriate Patient Population. The study included children beyond six months of age up to 18 years.
Heikens 1993 [63]	Inappropriate Patient Population, mean age of children at 1.2years.
Heikens 1993 [64]	Inappropriate Comparator, antibiotics compared with receiving health care from community healthcare aids
Hirschhorn 1971 [65]	Inappropriate Study Design, paper is a commentary on the current state of research.
Howard 1967 [66]	Inappropriate Study Design, all infants studied had acute diarrhea
Isaack 1992 [67]	Inappropriate Study Design, investigates nosocomial infections.
Isanaka 2020 [68]	Inappropriate Patient Population. All patients are above 6 months of age.
Islam 2021 [69]	Inappropriate Outcomes. Antibiotic usage was outcome not treatment group. Also all patients have diarrhea.
ISRCTN 2017 [70]	Wrong patient population. Patient age ranged from 2 months to 13 years, unable to obtain subgroup data for under 6 months.
Kabalo 2017 [71]	Inappropriate Patient Population. Patients aged over 6 months.
Lares-Asseff 1999 [72]	Inappropriate Study Design. Study evaluated blood levels for antibiotic not mortality outcome.
Lares-Asseff 2016 [73]	Inappropriate Comparator. Compared different doses of antibiotics to look for toxicity, not mortality.
Lattes 1954 [74]	Inappropriate Study Design and lack of full text.
Lebl 2001 [75]	Inappropriate Patient Population, age range 2 years to 18 years.

For peer review only

Antibiotic as an adjunct to treatment of severe acute malnutrition in infants less than six months of age. A protocol for the systematic review

List of Exclusions and Reasons for Exclusions.

Lelijveld 2021 [76]	Inappropriate Patient Population, age range 6 months to 59 months.
Lepage 1984 [77]	Inappropriate Study Design, patients all have bacterial infection treated with antibiotics.
Lewis 1956 [78]	Inappropriate Patient Population, patients all over 1 year old.
Maataoui 2020 [79]	Inappropriate Patient Population. Patients all over 6 months of age.
Macdougall 1957 [80]	Inappropriate Patient Population. Average age is 2 years.
MacLean 1980 [81]	Inappropriate Comparator. Antibiotic usage compared to enteral feeding.
Maitland 2006 [82]	Inappropriate Patient Population. Median age of 25 months.
Martischnig 1952 [83]	Inappropriate Intervention and lack of full text.
Mathew 2016 [84]	Wrong Patient Population. Patient all aged over 6months.
Mathew 2016 [85]	Wrong Patient Population. Patient all aged over 6months.
Melaku 1999 [86]	Inappropriate Patient Population. Patient median age 5.9 years.
Moschovis 2014 [87]	Inappropriate Study Design. Patients treated for pneumonia.
Mtango 1986 [88]	Inappropriate Intervention. Patients given antibiotics to treat acute respiratory infections.
Muhammad 2020 [89]	Inappropriate Comparator. Compares nutritional treatment with antibiotics usage
Mulholland 1995 [90]	Inappropriate Patient Population. All patients have pneumonia to treat.
Muller 1953 [91]	Inappropriate Study Design and lack of full text.
Murce 1955 [92]	Lack of Full Text.
Muwanguzi 2021 [93]	Inappropriate Patient Population. Children aged 1 year and above.
Nalwanga 2020 [94]	Inappropriate Patient Population. Mean age 14 months.
Nalwanga 2020 [95]	Inappropriate Patient Population. Mean age 14 months.
Nansumba 2018 [96]	Inappropriate Study Design. observational study
Nantanda 2008 [97]	Inappropriate patient population. paediatric patients with pneumonia
NCT 2009 [98]	Inappropriate patient population. paediatric patients with pneumonia
NCT 2009 [99]	Inappropriate Patient Population. children age 6 months to 5 years
NCT 2020 [100]	Inappropriate outcome. studied the effect of prophylactic azithromycin has on the microbiome
NCT 2015 [101]	Inappropriate patient population . hospitalized patients (not necessarily patients with diarrheal illness) receiving prophylactic course of azithromycin (not antibiotics to treat diarrheal illness) at the time of discharge, outcomes of malnourished patients not published
NCT 2012 [102]	Inappropriate patient population . age 6 months to 5 years
Nuzhat 2005 [103]	Inappropriate study design . observational study

For peer review only

Antibiotic as an adjunct to treatment of severe acute malnutrition in infants less than six months of age. A protocol for the systematic review

List of Exclusions and Reasons for Exclusions.

	Inappropriate population . children with a skin disease
O'Brien 2021 [104]	Inappropriate Patient Population . children age 6 to 59 months
O'Brien 2020 [105]	Inappropriate intervention . studied the effect on biannual prophylactic azithromycin (not antibiotics to treat a diarrheal illness) on the mortality rate of malnourished children
O'Brien 2020 [106]	Inappropriate study design . observational study<br>Inappropriate intervention . biannual prophylactic azithromycin (not antibiotics for diarrheal illness)
Page 2013 [107]	Inappropriate Patient Population . children age 6 to 59 months
Pai 1985 [108]	Inappropriate Study Design
Parpia 2020 [109]	Inappropriate Study Design . antibiotics were given prophylactically to the children in the study (not in the treatment of diarrheal illness)<br>Inappropriate patient population: children did not start receiving the prophylactic antibiotics until after 6 months of age
Pinto 2012 [110]	Inappropriate Setting . PICU<br>Inappropriate study design . observational study
Polster 1954 [111]	Lack of Full Text
Pombo 2017 [112]	Inappropriate patient population . patients were hospitalized with pneumonia, not diarrheal illness<br>Inappropriate study design . observational study
Prentice 2013 [113]	Inappropriate Study Design . this paper is a review of various studies on the management of malnutrition in children
Rasul 2006 [114]	Inappropriate Study Design . randomized control trial but no placebo control, controls were admitted to the hospital prior to the study and thus did not receive the WHO protocol<br>Inappropriate patient population . malnourished children age 1 month to 5 years, included hospitalizations for many reasons not just diarrheal illness<br>Inappropriate intervention . compared WHO protocol vs no WHO protocol, so patients in both groups may have received antibiotics and the difference between the groups includes many interventions other than antibiotics
Rawson 2016 [115]	Inappropriate Study Design . letter to the editor
Reed 1996 [116]	Inappropriate Intervention . desired outcome was the difference in incidence of bacteremia and the difference in mortality rate among bacteremic patients between malnourished and adequately nourished children<br>Inappropriate study design . observational study<br>Inappropriate patient population . age 0 to 5 years
Roboz 1955 [117]	Lack of Full Text
Rogawski 2015 [118]	Inappropriate Study design . observational study

Antibiotic as an adjunct to treatment of severe acute malnutrition in infants less than six months of age. A protocol for the systematic review

List of Exclusions and Reasons for Exclusions.

Rokstad 2013 [119]	Inappropriate Patient Population . this paper is a brief review of a study done by Trehan in 2013, which studied a population of children age 6 to 59 months
Rosenberg 1974 [120]	Inappropriate Study Design . review/opinion paper
Roy 2010 [121]	Inappropriate Patient Population . children age 5 to 12 years<br>Inappropriate indication . desired outcome as whether there was a difference in the pharmacokinetics of isoniazid in malnourished children vs adequately nourished children
Sala 1955 [122]	Inappropriate Intervention and lack of full text
Samotra 1985 [123]	Inappropriate Indication . desired outcome was whether or not there was a difference in the pharmacokinetics of gentamicin in malnourished children vs adequately nourished children
Samotra 1986 [124]	Inappropriate Indication . desired outcome was whether there was a difference in the pharmacokinetics of chloramphenicol in malnourished children vs adequately nourished children
Sanogo 2018 [125]	Inappropriate Indication . desired outcome was the level of adherence among healthcare providers to WHO guidelines regarding management (antibiotics, oral rehydration therapy, and zinc) of paediatric diarrheal illness in Bamako, Mali.
Santoro 2002 [126]	Inappropriate Study Design . observational study.<br>Inappropriate indication . desired outcome was to see how many children admitted to the PICU for lower respiratory tract infections needed ICU level care
Schapira 1971 [127]	Lack of Full Text
Sepehr 2009 [128]	Inappropriate Patient population . age 2 to 94 years<br>Inappropriate study design . observational study<br>Inappropriate indication . desired outcome was optimal length of post-operative antibiotic prophylaxis as well as risk factors associated with post-operative infection
Shahira 2002 [129]	Inappropriate Patient Population . population was all pediatric patients at a teaching hospital<br>Inappropriate study design . observational study<br>Inappropriate indication . desired outcome was risk factors for nosocomial infections
Soheir 1981 [130]	Inappropriate Indication . desired outcome was the appropriate dose of chloramphenicol for malnourished children, not mortality
Standing 2018 [131]	Inappropriate indication . studied the proper dose of IV ceftriaxone and PO metronidazole needed to achieve a therapeutic level in malnourished children
Tan 2020 [132]	Inappropriate Indication . studied the use of anthropometric measurements as opposed to clinical signs to identify malnourished children
Taubenslag 1950 [133]	Inappropriate Study Design .
Thame 2001 [134]	Inappropriate Study Design . observational study

For peer review only

Antibiotic as an adjunct to treatment of severe acute malnutrition in infants less than six months of age. A protocol for the systematic review

List of Exclusions and Reasons for Exclusions.

Thorson 1989 [135]	Inappropriate Indication .
Tilg 2013 [136]	Inappropriate Study Design . this paper is a review of a few different studies.<br>Inappropriate patient population . Of interest, it discusses the results from Trehan 2013, which has a patient population of children age 6 months to 59 months
Tornberg-Belanger 2017 [137]	Inappropriate Study Design . observational study<br>Inappropriate topic . studied adherence to antibiotic guidelines of various hospitals
Trehan 2010 [138]	Inappropriate Patient Population . was children age 6 to 59 months<br>Inappropriate study design . observational study
Trehan 2013 [139]	Inappropriate Patient Population . children age 6 to 59 months
Trehan 2016 [140]	Inappropriate Study Design . same exact study as trehan 2013<br>Inappropriate patient population . children age 6 to 59 months
Trehan 2016 [141]	Inappropriate Patient Population . letter to editor
Usman 2019 [142]	Inappropriate Study Design . observational study
Uzan-Yulzari 2021 [143]	Wrong patient population. Age of patients range from birth to 2 years.
Vather 2018 [144]	Inappropriate Study Design . observational study
Verani 2019 [145]	Inappropriate Study Design . observational study.
Viso Gurovich 2003 [146]	Inappropriate study design . observational study.<br>Inappropriate Intervention . studied the outcomes of paediatric patients who received antibiotics to treat various infections, most commonly bronchopneumonia, complicated pneumonia, and conjunctivitis.
Vygen 2013 [147]	Inappropriate Study Design . case series (descriptive study design) looking at the outcomes of malnourished infants who received care at a nutritional rehab center
Walsh 2018 [148]	Inappropriate Intervention . tested the effectiveness of a lactose free legume based nutritional feed to promote the resolution of diarrhea
Weingaertner 1961 [149]	Lack of Full Text
Wittmann 1967 [150]	Inappropriate Patient Population. age rage was 2 months to 2 years and not all were malnourished
Woodd-Walker 1972 [151]	Inappropriate Study Design, inappropriate patient population . age range was from 1 year to 4 years
Zecca 1951 [152]	Lack of Full Text and study is from 1951
BMJ 2013 [153]	Wrong study design. Research news article.
Langdon 2018 [154]	Wrong patient population. Patient ages ranged from 6 weeks to 60 weeks with no subgroup data available for under 6 months.
Journal of Agricultural and Food Chemistry 1954 [155]	Wrong study design. News report article.
Akush 1951 [156]	Wrong indication. Patients all treated for pneumonia.

For peer review only

Antibiotic as an adjunct to treatment of severe acute malnutrition in infants less than six months of age. A protocol for the systematic review

List of Exclusions and Reasons for Exclusions.

Alam 2020 [157]	Wrong study design. All patients treated for diarrhea.

For peer review only

Antibiotic as an adjunct to treatment of severe acute malnutrition in infants less than six months of age. A protocol for the systematic review

List of Exclusions and Reasons for Exclusions.

1. Abate BB, Tilahun BD, Kassie AM, Kassaw MW. Treatment outcome of Severe Acute Malnutrition and associated factors among under-five children in outpatient therapeutics unit in Gubalafto Wereda, North Wollo Zone, Ethiopia, 2019. PLoS One 2020;15(9):e0238231. DOI: 10.1371/journal.pone.0238231.
2. Adam A. [Antibacterial and symbiontic nutrition of infants; a contribution to the problem of dysbacteria]. Arztl Forsch 1954;8(4):I/168-74. (In und).
3. Adem F, Edessa D, Bayissa B, Mohammed Hassen M, Mohammed MA. Treatment Outcomes and Associated Factors in Hospitalised Children with Severe Acute Malnutrition: A Prospective Cohort Study. Pediatric Health Med Ther 2020;11:235-243. DOI: 10.2147/PHMT.S253396.
4. Ahmed T, Ali M, Ullah MM, et al. Mortality in severely malnourished children with diarrhoea and use of a standardised management protocol. The Lancet 1999;353(9168):1919-1922. DOI: https://doi.org/10.1016/S0140-6736(98)07499-6.
5. Ahmed T, Begum B, Badiuzzaman, Ali M, Fuchs G. Management of severe malnutrition and diarrhea. The Indian Journal of Pediatrics 2001;68(1):45-51. DOI: 10.1007/BF02728857.
6. AKBANI Y, BOLME P, LINDBLAD BS, RAHIMTOOLA RJ. CONTROL OF STREPTOMYCIN AND ISONIAZID IN MALNOURISHED CHILDREN TREATED FOR TUBERCULOSIS. Acta Paediatrica 1977;66(2):237-240. DOI: https://doi.org/10.1111/j.1651-2227.1977.tb07840.x.
7. Amorelli Gonzaga M, Alce M, Mesquita da Cunha MO, Da Sil va Peres PR. The efficacy of miocamycin in eutrophic and malnourished children. Jornal de Pediatria 1989;65(6):223-230.
8. Amza A, Kadri B, Nassirou B, et al. A cluster-randomized controlled trial evaluating the effects of mass azithromycin treatment on growth and nutrition in Niger. Am J Trop Med Hyg 2013;88(1):138-143. (In eng). DOI: 10.4269/ajtmh.2012.12-0284.
9. Amza A, Yu SN, Kadri B, et al. Does mass azithromycin distribution impact child growth and nutrition in Niger? A cluster-randomized trial. PLoS Negl Trop Dis 2014;8(9):e3128. (In eng). DOI: 10.1371/journal.pntd.0003128.
10. Angelakis E, Million M, Kankoe S, et al. Abnormal Weight Gain and Gut Microbiota Modifications Are Side Effects of Long-Term Doxycycline and Hydroxychloroquine Treatment. Antimicrobial Agents and Chemotherapy 2014;58(6):3342-3347. DOI: doi:10.1128/AAC.02437-14.
11. Ashorn P. Empirical antibiotic treatment for children suffering from dysentery, cholera, pneumonia, sepsis or severe acute malnutrition. Paediatrics and International Child Health 2018;38(sup1):S1-S2. DOI: 10.1080/20469047.2018.1443546.
12. Ashraf H, Alam N, Gyr N. 126 Randomized Controlled Trial of Day-Care and Hospitalized Management of Severe Pneumonia with Severe Acute Malnutrition in Children in Bangladesh. Archives of Disease in Childhood 2012;97(Suppl 2):A35-A35. DOI: 10.1136/archdischild-2012-302724.0126.
13. Autret E, Gendrel D, Breteau M. [Digestive absorption of gentamicin in malnourished children]. Presse Med 1989;18(15):776. (In fre).
14. Ayelign B, Abebe B, Shibeshi A, et al. Bacterial isolates and their antimicrobial susceptibility patterns among pediatric patients with urinary tract infections. Turk J Urol 2018;44(1):62-69. (In eng). DOI: 10.5152/tud.2017.33678.
15. Ayiyi B, Hazoume FA, Dan V. A review of purulent meningitis in children in Benin. Revue de Pediatrie 1987;23(4):156-160.
16. Aziz F, Patil P. Role of Prophylactic Vitamin K in Preventing Antibiotic Induced Hypoprothrombinemia. The Indian Journal of Pediatrics 2015;82(4):363-367. DOI: 10.1007/s12098-014-1584-3.
17. Berkley JA. Cotrimoxazole Prophylaxis in Severely Malnourished Children. Clinical Trials (US National Library of Medicine) 2009 (https://clinicaltrials.gov/ct2/show/NCT00934492).

Antibiotic as an adjunct to treatment of severe acute malnutrition in infants less than six months of age. A protocol for the systematic review

List of Exclusions and Reasons for Exclusions.

18. Berkley JA, Ngari M, Thitiri J, et al. Daily co-trimoxazole prophylaxis to prevent mortality in children with complicated severe acute malnutrition: a multicentre, double-blind, randomised placebo-controlled trial. Lancet Glob Health 2016;4(7):e464-73. DOI: 10.1016/S2214-109X(16)30096-1.
19. BERKOWITZ FE. Infections in children with severe protein-energy malnutrition. The Pediatric Infectious Disease Journal 1992;11(9):750-759. (https://journals.lww.com/pidj/Fulltext/1992/09000/Infections_in_children_with_severe_protein_energy.15.aspx).
20. Bhatnagar S, Bhan MK, Sazawal S, et al. Efficacy of Massive Dose Oral Gentamicin Therapy in Nonbloody Persistent Diarrhea with Associated Malnutrition. Journal of Pediatric Gastroenterology and Nutrition 1992;15(2):117-124. (https://journals.lww.com/jpgn/Fulltext/1992/08000/Efficacy_of_Massive_Dose_Oral_Gentamicin_Therapy.3.aspx).
21. Bhatnagar S, Bhan MK, Singh KD, Shrivastav R. Prognostic Factors in Hospitalized Children with Persistent Diarrhea: Implications for Diet Therapy. Journal of Pediatric Gastroenterology and Nutrition 1996;23(2):151-158. (https://journals.lww.com/jpgn/Fulltext/1996/08000/Prognostic_Factors_in_Hospitalized_Children_with.10.aspx).
22. Birindwa AM, Manegabe JT, Mindja A, Nordén R, Andersson R, Skovbjerg S. Decreased number of hospitalized children with severe acute lower respiratory infection after introduction of the pneumococcal conjugate vaccine in the Eastern Democratic Republic of the Congo. Pan Afr Med J 2020;37:211. (In eng). DOI: 10.11604/pamj.2020.37.211.22589.
23. Bleakly NT, Axelrod DM, Maldonado YA, Chisti MJ, Kache S. ABSTRACT 597: CLINICAL OUTCOMES AND MORTALITY IN MALNOURISHED CHILDREN BEFORE AND AFTER IMPLEMENTATION OF A PEDIATRIC SEPSIS PROTOCOL. Pediatric Critical Care Medicine 2014;15(4_suppl):135. DOI: 10.1097/01.pcc.0000449323.13704.d1.
24. Boesen I, SamsØE-Jensen T. [Intramuscular penicillin and oral dihydrostreptomycin therapy of acute dyspepsia in bottlefed infants under 7 months]. Nord Med 1953;50(29):1023-4. (In und).
25. Bolme P, Gebre-Ab T, Hadgu P, Meeuwisse G, Stintzing G. Absorption and elimination of penicillin in children with malnutrition. Ethiop Med J 1980;18(4):151-7. (In eng).
26. Bolme P, Eriksson M, Habte D, Paalzow L. Pharmacokinetics of streptomycin in Ethiopian children with tuberculosis and of different nutritional status. European Journal of Clinical Pharmacology 1988;33(6):647-649. DOI: 10.1007/BF00542504.
27. Bolme P, Eriksson M, Paalzow L, Stintzing G, Zerihun G, Woldemariam T. Malnutrition and Pharmacokinetics of Penicillin in Ethiopian Children. Pharmacology & Toxicology 1995;76(4):259-262. DOI: https://doi.org/10.1111/j.1600-0773.1995.tb00140.x.
28. Bravo ME, Arancibia A, Jarpa S, Carpentier PM, Jahn AN. Pharmacokinetics of gentamicin in malnourrished infants. European Journal of Clinical Pharmacology 1982;21(6):499-504. DOI: 10.1007/BF00542045.
29. Bravo IG. The pharmacokinetics of cotrimoxazole sulphonamide in malnourished (marasmic) infants. Pediatric Pharmacology 01-1984;4(3):167-176.
30. Bredow MT, Jackson AA. Community based, effective, low cost approach to the treatment of severe malnutrition in rural Jamaica. Archives of Disease in Childhood 1994;71(4):297-303. DOI: 10.1136/adc.71.4.297.
31. Brodwall K. Uncertain effect of antibiotics for malnourished children. Tidsskrift for Den Norske Laegeforening 2016;136(6):532.
32. Bruhn C. Reducing the inconceivable deficiency: Studies on the use of antibiotics in undernourished African children. Deutsche Apotheker Zeitung 2016;156(30):425-435.

For peer review only - http://bmjopen.bmj.com/site/about/guidelines.xhtml

Antibiotic as an adjunct to treatment of severe acute malnutrition in infants less than six months of age. A protocol for the systematic review

List of Exclusions and Reasons for Exclusions.

33. Brunozi NA, David FL. Antibioticoterapia em crianças indígenas e não indígenas desnutridas. ABCS health sci 44(2):85-91.
34. Buchanan N. Letter To The Editor. Acta Paediatrica 1977;66(5):663-663. DOI: https://doi.org/10.1111/j.1651-2227.1977.tb07966.x.
35. Buchanan N, Eyberg C. Intramuscular tobramycin administration in kwashiorkor. S Afr Med J 1978;53(8):273-4. (In eng).
36. Buchanan N, Davis M, Eyberg C. Gentamicin pharmacokinetics in kwashiorkor. British Journal of Clinical Pharmacology 1979;8(5):451-453. DOI: https://doi.org/10.1111/j.1365-2125.1979.tb01024.x.
37. Bunn JEG. Severe acute malnutrition and HIV in African children. Future HIV Therapy 2009;3(6):595-611.
38. Cakir FB, Aydogan G, Timur C, et al. Effects of malnutrition on oxidative burst functions and infection episodes in children with acute lymphoblastic leukemia. International Journal of Laboratory Hematology 2012;34(6):648-654. DOI: https://doi.org/10.1111/j.1751-553X.2012.01451.x.
39. Çaksen H, Cesur Y, Üner A, et al. Urinary tract infection and antibiotic susceptibility in malnourished children. International Urology and Nephrology 2000;32(2):245-247. DOI: 10.1023/A:1007104326689.
40. Caksen H, Arslan S, Cesur Y, Sar S, Celebi V, Kuru M. Urinary tract infection and antibiotic susceptibility in malnourished children. Ceylon Med J 2000;45(2):77-9. (In eng). DOI: 10.4038/cmj.v45i2.8007.
41. Cedrato A, Larguia A, Galan HM. Administration of cefapirin every 12 hours in children with pneumonia: response according to protein nutritional status. Folha Medica 1974;68(1):1-4.
42. Chisti MJ, Salam MA, Bardhan PK, et al. Treatment Failure and Mortality amongst Children with Severe Acute Malnutrition Presenting with Cough or Respiratory Difficulty and Radiological Pneumonia. PLOS ONE 2015;10(10):e0140327. DOI: 10.1371/journal.pone.0140327.
43. Church JA, Fitzgerald F, Walker AS, Gibb DM, Prendergast AJ. The expanding role of co-trimoxazole in developing countries. The Lancet Infectious Diseases 2015;15(3):327-339. DOI: 10.1016/S1473-3099(14)71011-4.
44. Cochlovius A, Daecke K, Loeschke A. [Effect of various antibiotics in infant nutrition disorders]. Dtsch Med Wochenschr 1953;78(37):1266-70. (In und). DOI: 10.1055/s-0028-1114923.
45. Dicko A, Ouedraogo JB, Zongo I, et al. A trial of seasonal malaria chemoprevention plus azithromycin in African children. The American Society of Tropical Medicine and Hygiene 2016;95(5):481. (In English). DOI: 10.4269/ajtmh.abstract2016.
46. Dieng H, Aboussaleh Y, Sbaibi R, Bengueddour R. Evaluation of the care of infants and children under 5 years with severe undernutrition at the CRENAS of Nouakchott, Mauritania. Nutritional Therapy & Metabolism 2014;32:140-145. DOI: 10.5301/NTM.2014.12648.
47. Dubray C, Ibrahim SA, Abdelmutalib M, et al. Treatment of severe malnutrition with 2-day intramuscular ceftriaxone vs 5-day amoxicillin. Annals of Tropical Paediatrics 2008;28(1):13-22. DOI: 10.1179/146532808X270635.
48. Duffau G, Alvarez P, Chala E, Cruz C. [Manifestations of paralytic ileus in infants hospitalized for acute diarrheal syndrome]. Rev Chil Pediatr 1990;61(6):322-6. (In spa).
49. Dutta S, Chowdhary G, Kumar P, Mukhopadhay K, Narang A. Ciprofloxacin Administration to Very Low Birth Weight Babies has no Effect on Linear Growth in Infancy. Journal of Tropical Pediatrics 2005;52(2):103-106. DOI: 10.1093/tropej/fmi070.

For peer review only

Antibiotic as an adjunct to treatment of severe acute malnutrition in infants less than six months of age. A protocol for the systematic review

List of Exclusions and Reasons for Exclusions.

50. Eriksson M, Paalzow L, Bolme P, Mariam TW. Chloramphenicol pharmacokinetics in Ethiopian children of differing nutritional status. European Journal of Clinical Pharmacology 1983;24(6):819-823. DOI: 10.1007/BF00607094.
51. ERIKSSON M, BOLME P, HABTE D, PAALZOW L. INH and Streptomycin in Ethiopian Children with Tuberculosis and Different Nutritional Status. Acta Paediatrica 1988;77(6):890-894. DOI: https://doi.org/10.1111/j.1651-2227.1988.tb10774.x.
52. Ford GW, Belbin R, Jose DG, Vorbach EA, Kirke DK. Growth and Immune Function in Aboriginal Children during Recovery from Malnutrition and Infection. Australian and New Zealand Journal of Medicine 1976;6(4):321-328. DOI: https://doi.org/10.1111/imj.1976.6.4.321.
53. Friedrich MJ. Antibiotics of No Benefit in Treating Children With Malnutrition. JAMA 2016;315(11):1100-1100. DOI: 10.1001/jama.2016.2266.
54. Garcia de Olarte D, Trujillo H, Agudelo N, Nelson JD, Haltalin KC. Treatment of diarrhea in malnourished infants and children. A double-blind study comparing ampicillin and placebo. Am J Dis Child 1974;127(3):379-88. DOI: 10.1001/archpedi.1974.02110220077011.
55. Gardner R, Dwyer C, Morrison C. MINERVA. BMJ: British Medical Journal (Overseas & Retired Doctors Edition) 2011;343(7821):486.
56. Gennaro S, O'Connor C, Marx M. Global Health of Babies and Children. MCN: The American Journal of Maternal/Child Nursing 2017;42(3):132-138. DOI: 10.1097/nmc.0000000000000322.
57. Gensch F, Burmann R, Goertler H. Anstaltsdyspepsien und Kolisubstitution. Dtsch Med Wochenschr 1957;82(20):810-818. (In De).
58. Gernaat HBPE, Dechering WHJC, Voorhoeve HWA. Mortality in Severe Protein-energy Malnutrition at Nchelenge, Zambia. Journal of Tropical Pediatrics 1998;44(4):211-217. DOI: 10.1093/tropej/44.4.211.
59. Ghosh G, Chakraborty S, Ray J, Mukherjee SK. Parenteral ciprofloxacin in persistent diarrhoea in children. J Indian Med Assoc 1995;93(10):382, 384. (In eng).
60. Girma S, Sisay M, Mengistu G, Amare F, Edessa D. Antimicrobial Utilization Pattern in Pediatric Patients in Tertiary Care Hospital, Eastern Ethiopia: The Need for Antimicrobial Stewardship. Hosp Pharm 2018;53(1):44-54. DOI: 10.1177/0018578717737429.
61. Gore-Langton GR, Cairns M, Compaore YD, et al. Effect of adding azithromycin to the antimalarials used for seasonal malaria chemoprevention on the nutritional status of African children. Trop Med Int Health 2020;25(6):740-750. DOI: 10.1111/tmi.13390.
62. Hecht C, Weber M, Grote V, et al. Disease associated malnutrition correlates with length of hospital stay in children. Clin Nutr 2015;34(1):53-9. (In eng). DOI: 10.1016/j.clnu.2014.01.003.
63. Heikens GT, Schofield WN, Christie CD, Gernay J, Dawson S. The Kingston Project. III. The effects of high energy supplement and metronidazole on malnourished children rehabilitated in the community: morbidity and growth. Eur J Clin Nutr 1993;47(3):174-91. (In eng).
64. Heikens GT, Schofield WN, Dawson S. The Kingston Project. II. The effects of high energy supplement and metronidazole on malnourished children rehabilitated in the community: anthropometry. Eur J Clin Nutr 1993;47(3):160-73. (In eng).
65. Hirschhorn N. Can small daily doses of antibiotics prevent the cycle of diarrhea, malabsorption, and malnutrition in children? The American Journal of Clinical Nutrition 1971;24(7):872-875. DOI: 10.1093/ajcn/24.7.872.
66. Howard JE, Beca JP. CLINICAL ASSESSMENT OF HETACILLIN IN ACUTE DIARRHEA OF MALNOURISHED INFANTS. Annals of the New York Academy of Sciences 1967;145(2):436-441. DOI: https://doi.org/10.1111/j.1749-6632.1967.tb50243.x.

For peer review only

Antibiotic as an adjunct to treatment of severe acute malnutrition in infants less than six months of age. A protocol for the systematic review

List of Exclusions and Reasons for Exclusions.

67. Isaack H, Mbise RL, Hirji KF. Nosocomial bacterial infections among children with severe protein energy malnutrition. East Afr Med J 1992;69(8):433-6. (In eng).
68. Isanaka S, Grantz KH, Berthé F, Schaefer M, Adehossi E, Grais RF. Extended Follow-up From a Randomized Clinical Trial of Routine Amoxicillin in the Treatment of Uncomplicated Severe Acute Malnutrition in Niger. JAMA Pediatrics 2020;174(3):295-297. DOI: 10.1001/jamapediatrics.2019.5189.
69. Islam MR, Nuzhat S, Fahim SM, et al. Antibiotic exposure among young infants suffering from diarrhoea in Bangladesh. Journal of Paediatrics and Child Health 2021;57(3):395-402. DOI: https://doi.org/10.1111/jpc.15233.
70. ISRCTN. A study to compare antibiotics used to treat children with severe acute malnutrition. 2017 (https://trialsearch.who.int/?TrialID=ISRCTN18051843).
71. Kabalo MY, Seifu CN. Treatment outcomes of severe acute malnutrition in children treated within Outpatient Therapeutic Program (OTP) at Wolaita Zone, Southern Ethiopia: retrospective cross-sectional study. Journal of Health, Population and Nutrition 2017;36(1):7. DOI: 10.1186/s41043-017-0083-3.
72. Lares-Asseff I, Lugo-Goytia G, Pérez-Guillé MG, et al. [Bayesian prediction of chloramphenicol blood levels in children with sepsis and malnutrition]. Rev Invest Clin 1999;51(3):159-65. (In spa).
73. Lares-Asseff I, Pérz-Guillé MG, Camacho Vieyra GA, Pérez AG, Peregrina NB, Lugo Goytia G. Population Pharmacokinetics of Gentamicin in Mexican Children With Severe Malnutrition. The Pediatric Infectious Disease Journal 2016;35(8):872-878. DOI: 10.1097/inf.0000000000001204.
74. Lattes E, Piovano L. [Treatment of acute infant nutrition disorders and tests of sensitivity to antibiotics; clinical considerations]. Minerva Pediatr 1954;6(7):247-8. (In und).
75. Lebl J, Zahradníková M, Bartosová J, Zemková D, Pechová M, Vávrová V. Insulin-like growth factor-I and insulin-like growth factor-binding protein-3 in cystic fibrosis: a positive effect of antibiotic therapy and hyperalimentation. Acta Paediatr 2001;90(8):868-72. (In eng).
76. Lelijveld N, Godbout C, Krietemeyer D, et al. Treating high-risk moderate acute malnutrition using therapeutic food compared with nutrition counseling (Hi-MAM Study): a cluster-randomized controlled trial. The American Journal of Clinical Nutrition 2021;114(3):955-964. DOI: 10.1093/ajcn/nqab137.
77. Lepage P, Bogaerts J, Nsengumuremyi F, et al. Severe multiresistant Salmonella typhimurium systemic infections in Central Africa – clinical features and treatment in a paediatric department. Journal of Antimicrobial Chemotherapy 1984;14(suppl_B):153-159. DOI: 10.1093/jac/14.suppl_B.153.
78. Lewis RA, Bhagat MP, Wagle MM, Kulkarni BS, Satoskar RS. Antibiotic Dietary Supplements in the Therapy of Childhood Protein Mainutrition. The American Journal of Tropical Medicine and Hygiene 1956;5(3):483-496. (In English). DOI: 10.4269/ajtmh.1956.5.483.
79. Maataoui N, Langendorf C, Berthe F, et al. Increased risk of acquisition and transmission of ESBL-producing Enterobacteriaceae in malnourished children exposed to amoxicillin. Journal of Antimicrobial Chemotherapy 2019;75(3):709-717. DOI: 10.1093/jac/dkz487.
80. MACDOUGALL LG. THE EFFECT OF AUREOMYCIN ON UNDERNOURISHED AFRICAN CHILDREN. Journal of Tropical Pediatrics 1957;3(2):74-81. DOI: 10.1093/oxfordjournals.tropej.a057461.
81. MacLean WC, Jr., Lopez de Romaña G, Massa E, Graham GG. Nutritional management of chronic diarrhea and malnutrition: Primary reliance on oral feeding. The Journal of Pediatrics 1980;97(2):316-323. DOI: 10.1016/S0022-3476(80)80507-5.
82. Maitland K, Berkley JA, Shebbe M, Peshu N, English M, Newton CR. Children with severe malnutrition: can those at highest risk of death be identified with the WHO protocol? PLoS Med 2006;3(12):e500. (In eng). DOI: 10.1371/journal.pmed.0030500.

For peer review only

Antibiotic as an adjunct to treatment of severe acute malnutrition in infants less than six months of age. A protocol for the systematic review

List of Exclusions and Reasons for Exclusions.

83. Martischnig E. [Antibiotic therapy of severe nutritional disorders in infants]. Ann Paediatr 1952;179(3):168-87. (In und).
84. Mathew JL. Does Routine Antibiotic Therapy Benefit Children With Severe Acute Malnutrition?: Evidence-based Medicine Viewpoint. Indian Pediatr 2016;53(4):329-32. (In eng). DOI: 10.1007/s13312-016-0846-8.
85. Mathew JL, Rai R. Does routine antibiotic therapy benefit children with severe acute malnutrition? Indian Pediatrics 2016;53(4):329-333. DOI: 10.1007/s13312-016-0846-8.
86. Melaku A, Lulseged S. Chronic suppurative otitis media in a children's hospital in Addis Ababa, Ethiopia. Ethiop Med J 1999;37(4):237-46. (In eng).
87. Moschovis PP, Addo-Yobo EOD, Banajeh S, et al. Stunting is associated with poor outcomes in childhood pneumonia. Tropical medicine & international health : TM & IH 2015;20(10):1320-1328. (In eng). DOI: 10.1111/tmi.12557.
88. Mtango FDE, Neuvians D. Acute respiratory infections in children under five years. Control project in Bagamoyo District, Tanzania. Transactions of The Royal Society of Tropical Medicine and Hygiene 1986;80(6):851-858. DOI: 10.1016/0035-9203(86)90241-5.
89. Muhammad A SY, Nisar MI, Baloch B, Yazdani AT, Yazdani N, Jehan F. . Nutritional support for lactating women with or without azithromycin for infants compared to breastfeeding counseling alone in improving the 6-month growth outcomes among infants of peri-urban slums in Karachi, Pakistan-the protocol for a multiarm assessor-blinded randomized controlled trial Trials 2020;1(21):756.
90. MULHOLLAND EK, FALADE AG, CORRAH PT, et al. A randomized trial of chloramphenicol vs. trimethoprim-sulfamethoxazole for the treatment of malnourished children with community-acquired pneumonia. The Pediatric Infectious Disease Journal 1995;14(11):959-964. (https://journals.lww.com/pidj/Fulltext/1995/11000/A_randomized_trial_of_chloramphenicol_vs_.7.aspx).
91. Muller R. [Antibiotic therapy of nutrition disorders]. Kinderarztl Prax 1953:195-200. (In ger).
92. Murce A. [Current problems concerning etiology and therapy of acute nutritional disorders in early infancy; effect of chemotherapeutic agents and antibiotics]. Rev Bras Med 1955;12(5):306-13. (In por).
93. Muwanguzi E, Oboi JE, Nabbamba A, Wanyama R. Treatment outcome and associated factors for severely malnourished children (1–5 years) admitted to Lacor Hospital and Gulu Regional Referral Hospital in Uganda. Journal of Nutritional Science 2021;10:e33. DOI: 10.1017/jns.2021.11.
94. Nalwanga D, Musiime V, Kizito S, et al. Mortality among children under five years admitted for routine care of severe acute malnutrition: a prospective cohort study from Kampala, Uganda. BMC Pediatrics 2020;20(1):182. DOI: 10.1186/s12887-020-02094-w.
95. Nalwanga D, Musiime V, Kizito S, Kiggundu JB, Musoke P, Tumwine JK. Mortality among Children Hospitalized for Routine Care of Severe Acute Malnutrition. Pediatrics 2020;146(1 MeetingAbstract):299-299. DOI: 10.1542/peds.146.1_MeetingAbstract.299.
96. Nansumba M, Kumbakumba E, Orikiriza P, et al. Treatment outcomes and tolerability of the revised WHO anti-tuberculosis drug dosages for children. The International Journal of Tuberculosis and Lung Disease 2018;22(2):151-157. DOI: 10.5588/ijtld.17.0535.
97. Nantanda R, Hildenwall H, Peterson S, Kaddu-Mulindwa D, Kalyesubula I, K.Tumwine J. Bacterial aetiology and outcome in children with severe pneumonia in Uganda. Annals of Tropical Paediatrics 2008;28(4):253-260. DOI: 10.1179/146532808X375404.
98. International Centre for Diarrhoeal Disease Research B. Trial in Childhood Pneumonia With Malnutrition. 2009 (https://clinicaltrials.gov/ct2/show/NCT00968370).

For peer review only

Antibiotic as an adjunct to treatment of severe acute malnutrition in infants less than six months of age. A protocol for the systematic review

List of Exclusions and Reasons for Exclusions.

99. Medicine WUSo. Effectiveness of Oral Antibiotics in the Treatment of Severe Acute Malnutrition. 2009 (https://clinicaltrials.gov/ct2/show/NCT01000298).
100. University of California SF. Gut and Azithromycin Mechanisms in Infants and Children II (GAMINII). 2020 (https://clinicaltrials.gov/ct2/show/NCT04315272).
101. Washington Uo. Azithromycin to Prevent Post-discharge Morbidity and Mortality in Kenyan Children (Toto Bora). 2015 (https://clinicaltrials.gov/ct2/show/NCT02414399).
102. Epicentre. The Effect of Routine Antibiotic Use in the Outpatient Treatment of Severely Malnourished Children Without Complications. 2012 (https://clinicaltrials.gov/ct2/show/NCT01613547).
103. Yasmeen N, Khan MR. Spectrum of common childhood skin diseases: a single centre experience. J Pak Med Assoc 2005;55(2):60-3. (In eng).
104. O'Brien KS, Sié A, Dah C, et al. Azithromycin for uncomplicated severe acute malnutrition: study protocol for a pilot randomized controlled trial. Pilot and Feasibility Studies 2021;7(1):97. DOI: 10.1186/s40814-021-00836-w.
105. O'Brien KS, Arzika AM, Maliki R, et al. Biannual azithromycin distribution and child mortality among malnourished children: A subgroup analysis of the MORDOR cluster-randomized trial in Niger. PLoS Med 2020;17(9):e1003285. DOI: 10.1371/journal.pmed.1003285.
106. Kieran S. O'Brien AMA, Ramatou Maliki,Farouk Manzo,Alio K. Mamkara,Elodie Lebas,Catherine Cook,Robin L. Bailey,Sheila K. West,Catherine E. Oldenburg,Travis C. Porco,Benjamin Arnold,Jeremy D. Keenan,Thomas M. Lietman. The role of nutritional status in the efficacy of azithromycin to reduce child mortality in Niger: A subgroup analysis of the mordor trial. American Journal of Tropical Medicine and Hygiene 2019. DOI: 10.4269/ajtmh.abstract2019.
107. Page A-L, de Rekeneire N, Sayadi S, et al. Infections in Children Admitted with Complicated Severe Acute Malnutrition in Niger. PLOS ONE 2013;8(7):e68699. DOI: 10.1371/journal.pone.0068699.
108. Pai PM, Desai M, Vachharajani A. Drug therapy in malnourished children. 1985;25:25-28.
109. Parpia TC, Elwood SE, Scharf RJ, et al. Baseline Characteristics of Study Participants in the Early Life Interventions for Childhood Growth and Development in Tanzania (ELICIT) Trial. The American Journal of Tropical Medicine and Hygiene 2020;103(4):1397-1404. (In English). DOI: 10.4269/ajtmh.19-0918.
110. Pinto V. American Academy of Pediatrics Section on Critical Care Scientific Program Scientific Abstracts and Posters AAP National Conference and Exhibition October 21, 2012 – New Orleans, LA. Pediatric Critical Care Medicine 2012;13(6):704-710. DOI: 10.1097/PCC.0b013e31826df088.
111. Polster H. [Chloramphenicol treatment of acute nutrition disorders]. Kinderarztl Prax 1954;22(3):109-13. (In und).
112. Pombo-March P. Clinical profile of children and adolescents with and without comorbidities hospitalized with community acquired pneumonia. Pediatric Pulmonology 2017;52(S46):S100-S176. DOI: https://doi.org/10.1002/ppul.23731.
113. Prentice AM, Nabwera H, Kwambana B, Antonio M, Moore SE. Microbes and the Malnourished Child. Science Translational Medicine 2013;5(180):180fs11-180fs11. DOI: doi:10.1126/scitranslmed.3006212.
114. Rasul C, Aa M, Hasanuzzaman. Outcome of management of severe malnutrition in a medical institution. Paediatrics, Child and Adolescent Health Journal 2006;Paediatrics, Child and Adolescent Health Journal (Singapore) 2006; 46: 156-60.
115. Grais TMRLMAHITKSMMSIEAR. Amoxicillin for Severe Acute Malnutrition in Children. New England Journal of Medicine 2016;375:190-192. (https://www.nejm.org/doi/pdf/10.1056%2FNEJMc1605388).

For peer review only

Antibiotic as an adjunct to treatment of severe acute malnutrition in infants less than six months of age. A protocol for the systematic review

List of Exclusions and Reasons for Exclusions.

116. Reed RP, Wegerhoff FO, Rothberg AD. Bacteraemia in malnourished rural African children. Annals of Tropical Paediatrics 1996;16(1):61-68. DOI: 10.1080/02724936.1996.11747805.

117. Roboz P, Kadar M. [Increase of weight in infant dystrophy following isoniazid therapy]. Orv Hetil 1955;96(9):234-5. (In hun).

118. Rogawski ET, Westreich DJ, Adair LS, et al. Early Life Antibiotic Exposure Is Not Associated with Growth in Young Children of Vellore, India. J Pediatr 2015;167(5):1096-102.e3. (In eng). DOI: 10.1016/j.jpeds.2015.08.015.

119. Rokstad IS. Antibiotics in severe malnutrition in children. 2013. DOI: 10.4045/tidsskr.13.0366.

120. Rosenberg IH, Beisel WR, Gordon JE, et al. Infant and child enteritis-malabsorption-malnutrition: the potential of limited studies with low-dose antibiotic feeding. The American Journal of Clinical Nutrition 1974;27(3):304-309. DOI: 10.1093/ajcn/27.3.304.

121. Roy V, Gupta D, Gupta P, Sethi GR, Mishra TK. Pharmacokinetics of isoniazid in moderately malnourished children with tuberculosis. Int J Tuberc Lung Dis 2010;14(3):374-6. (In eng).

122. Sala F. [Observations on the use of combined chloramphenicol-sulfonamide therapy in acute nutritional disorders in pediatrics]. Gazz Med Ital 1955;114(3):54-8. (In ita).

123. Samotra K, Gupte S, Raina RK. Pharmacokinetics of gentamicin in protein-energy malnutrition. European Journal of Clinical Pharmacology 1985;29(2):255-256. DOI: 10.1007/BF00547433.

124. Samotra K, Gupte S, Raina RK. Effect of malnutrition on chloramphenicol kinetics in Indian children. Zhongguo Yao Li Xue Bao 1986;7(2):162-4. (In eng).

125. Sanogo D, Keita A, Roose A, et al. MANAGEMENT OF DIARRHEA IN MALI: ASSESSMENT OF THE ADHERENCE TO WHO RECOMMENDATIONS IN THE VACCINE IMPACT ON DIARRHEA IN AFRICA (VIDA) STUDY. AMERICAN JOURNAL OF TROPICAL MEDICINE AND HYGIENE: AMER SOC TROP MED & HYGIENE 8000 WESTPARK DR, STE 130, MCLEAN, VA 22101 USA; 2018:591-591.

126. SANTORO A, FERREIRA E, FERRARI AM. Infecciones respiratorias agudas bajas en niños menores de tres años: Referencia a unidades de cuidado intensivo. Archivos de Pediatría del Uruguay 2002;73:196-202. (http://www.scielo.edu.uy/scielo.php?script=sci_arttext&pid=S1688-12492002000400003&nrm=iso).

127. Schapira Y, Richtie J. [Combined therapy of Pseudomonas infection and severe malnutrition]. Harefuah 1971;80(11):608-10. (In heb).

128. Sepehr A, Santos B-JG, Chou C, et al. Antibiotics in head and neck surgery in the setting of malnutrition, tracheotomy, and diabetes. The Laryngoscope 2009;119(3):549-553. DOI: https://doi.org/10.1002/lary.20078.

129. Shahira S. BHAR, El Metwaly; Shoikar, Abdel Salam. Bacterial nosocomial infections among children admitted to wards at El-Shatby university children's hospital. Alexandria Journal of Pediatrics 2002;16:297-304.

130. Soheir M. DH, Shatla; Mona, Salem; Laila, El Sayed; Tahani E.S., Kholeif. Pharmacokinetics of cefazolin and chloramphenicol in protein-energy malnuirition. Journal of the Egyptian Society of Pharmacology and Experimental Therapeutics 1981;2:91-100.

131. Standing JF, Ongas MO, Ogwang C, et al. Dosing of Ceftriaxone and Metronidazole for Children With Severe Acute Malnutrition. Clinical Pharmacology & Therapeutics 2018;104(6):1165-1174. DOI: https://doi.org/10.1002/cpt.1078.

132. Tan R, Kagoro F, Levine GA, et al. Clinical Outcome of Febrile Tanzanian Children with Severe Malnutrition Using Anthropometry in Comparison to Clinical Signs. The American Journal of Tropical Medicine and Hygiene 2020;102(2):427-435. (In English). DOI: 10.4269/ajtmh.19-0553.

For peer review only

Antibiotic as an adjunct to treatment of severe acute malnutrition in infants less than six months of age. A protocol for the systematic review

List of Exclusions and Reasons for Exclusions.

133. Taubenslag L. [Streptomycin in acute nutritional disorders of infants in first half-year]. Dia Med 1950;22(48):1965-9. (In und).
134. Thame M, Stephen C, Wilks R, Forrester TE. The appropriateness of the current antibiotic empiric therapy based on the bacteria isolated from severely malnourished Jamaican children. West Indian Med J 2001;50(2):140-3. (In eng).
135. Thorson SM. Multiple-drug resistant Salmonella enteritis in malnourished children: Dramatic response to cefotaxime. 1989;11:279-287.
136. Tilg H, Moschen AR. Malnutrition and microbiota—a new relationship? Nature Reviews Gastroenterology & Hepatology 2013;10(5):261-262. DOI: 10.1038/nrgastro.2013.48.
137. Tornberg-Belanger S, Tickell K, Mangale D, et al. Pediatric inpatient antibiotic prescription practices in the chain network hospitals at baseline. American Society of Tropical Medicine and Hygiene; 2017.
138. Trehan I, Amthor RE, Maleta K, Manary MJ. Evaluation of the routine use of amoxicillin as part of the home-based treatment of severe acute malnutrition. Tropical Medicine & International Health 2010;15(9):1022-1028. (https://doi.org/10.1111/j.1365-3156.2010.02580.x). DOI: https://doi.org/10.1111/j.1365-3156.2010.02580.x.
139. Trehan I, Goldbach HS, LaGrone LN, et al. Antibiotics as Part of the Management of Severe Acute Malnutrition. New England Journal of Medicine 2013;368(5):425-435. DOI: 10.1056/NEJMoa1202851.
140. Trehan I, Goldbach HS, LaGrone LN, et al. Antibiotics as part of the management of severe acute malnutrition. Malawi Med J 2016;28(3):123-130. (In eng).
141. Trehan I. Amoxicillin for Severe Acute Malnutrition in Children. New England Journal of Medicine 2016;375(2):190-192. DOI: 10.1056/NEJMc1605388.
142. Farina Usman SS, Muhammad Amir, Muhammad Nadeem Chohan, Muhammad Touseef. Efficacy of Ampicillin and Gentamycin Combination Therapy in Children Having Severe Acute Malnutrition Presenting with Acute Diarrhea at Civil Hospital Hyderabad. Medical Forum Monthly 2019.
143. Atara Uzan-Yulzari OT, Anna Belogolovski, Oren Ziv, Christina Kunz, Sarah Perschbacher, Hadar Neuman, Edoardo Pasolli, Aia Oz, Hila Ben-Amram, Himanshu Kumar, Helena Ollila, Anne Kaljonen, Erika Isolauri, Seppo Salminen, Hanna Lagström, Nicola Segata, Itai Sharon, Yoram Louzoun, Regina Ensenauer,. Neonatal antibiotic exposure impairs child growth during the first six years of life by perturbing intestinal microbial colonization. Nature Communications 2021. DOI: 10.1038/s41467-020-20495-4.
144. Vather A, McKerrow NH, Morgan ME. Factors underlying the changing of antibiotics in a paediatric intensive care unit in Pietermaritzburg, KwaZulu-Natal2018.
145. Verani. Abstract Book. The American Journal of Tropical Medicine and Hygiene 2019;101(5_Suppl):1-668. (In English). DOI: 10.4269/ajtmh.abstract2019.
146. Gurovich F, Gómez-Oliván L, Velasco JM. Assessment of the use of chloramphenicol at the Neonatal Unit of a Mexican Paediatric Hospital. Pharmaceutical Care Espana 2003;5:182-185.
147. Vygen SB, Roberfroid D, Captier V, Kolsteren P. Treatment of Severe Acute Malnutrition in Infants Aged <6 Months in Niger. The Journal of Pediatrics 2013;162(3):515-521.e3. DOI: 10.1016/j.jpeds.2012.09.008.
148. Walsh K, Calder N, Olupot-Olupot P, et al. Modifying Intestinal Integrity and MicroBiome in Severe Malnutrition with Legume-Based Feeds (MIMBLE 2.0): protocol for a phase II refined feed and intervention trial [version 1; peer review: 2 approved]. Wellcome Open Research 2018;3(95). DOI: 10.12688/wellcomeopenres.14706.1.
149. Weingaertner L. [A new antibiotic, colistin, in the treatment of infant nutrition disorders]. Munch Med Wochenschr 1961;103:1628-31. (In ger).

Antibiotic as an adjunct to treatment of severe acute malnutrition in infants less than six months of age. A protocol for the systematic review

List of Exclusions and Reasons for Exclusions.

150. Wittmann W, Moodie AD, Hansen JD. A comparison of two antibiotic regimens in the treatment of gastroenteritis in malnourished children with a one-year follow-up of their subsequent progress. S Afr Med J 1967;41(16):414-7. (In eng).
151. WOODD-WALKER RB, HANSEN JDL, SAUNDERS SJ. THE IN VITRO UPTAKE OF LYSINE AND ALANINE BY HUMAN JEJUNAL MUCOSA IN PROTEIN-CALORIE MALNUTRITION, IN GASTROENTERITIS AND AFTER NEOMYCIN. Acta Paediatrica 1972;61(2):140-144. DOI: https://doi.org/10.1111/j.1651-2227.1972.tb15917.x.
152. Zecca E. [Streptomycin therapy in acute and chronic nutrition disorders in hospital]. Lattante 1951;22(9):536-40. (In und).
153. BMJ. Treating malnutrition with antibiotics. 2013:356. (https://regroup-production.s3.amazonaws.com/documents/ReviewReference/357822763/Treating%20malnutrition%20with%20antibiotics.pdf?AWSAccessKeyId=AKIAJBZQODCMKJA4H7DA&Expires=1643053445&Signature=SvDdx2aaEu%2B8i5CwDvlRAgoL52s%3D).
154. Amy Elizabeth Langdon XS, Sheila Isanaka, Gautam, Dantas. Prophylactic broad-spectrum antibiotics for childhood malnutrition. Journal of clinical and translational science 2018:46. (https://regroup-production.s3.amazonaws.com/documents/ReviewReference/357814158/Langdon%202018%20%20.pdf?AWSAccessKeyId=AKIAJBZQODCMKJA4H7DA&Expires=1643053619&Signature=UGJETRPj9mELVYWfjDUkVF%2Fhmqw%3D).
155. News: Antibiotic Supplements Overcome Growth Failure in School Children. Journal of Agricultural and Food Chemistry 1954;2(23):1148-1148. DOI: 10.1021/jf60043a605.
156. [Antibiotic albomycin in the treatment of pneumonias and toxemias in infants during the first year of life]. Feldsher Akush 1951;12:38. (In und).
157. Alam T, Ahmed D, Ahmed T, et al. A double-blind placebo-controlled trial of azithromycin to reduce mortality and improve growth in high-risk young children with non-bloody diarrhoea in low resource settings: the Antibiotics for Children with Diarrhoea (ABCD) trial protocol. Trials 2020;21(1):71. DOI: 10.1186/s13063-019-3829-y.

PRISMA 2020 Checklist

Section and Topic	Item #	Checklist item	Location where item is reported
**TITLE**			
Title	1	Identify the report as a systematic review.	p1
**ABSTRACT**			
Abstract	2	See the PRISMA 2020 for Abstracts checklist.	p3
**INTRODUCTION**			
Rationale	3	Describe the rationale for the review in the context of existing knowledge.	p4
Objectives	4	Provide an explicit statement of the objective(s) or question(s) the review addresses.	p4
**METHODS**			
Eligibility criteria	5	Specify the inclusion and exclusion criteria for the review and how studies were grouped for the syntheses.	p4
Information sources	6	Specify all databases, registers, websites, organisations, reference lists and other sources searched or consulted to identify studies. Specify the date when each source was last searched or consulted.	p5
Search strategy	7	Present the full search strategies for all databases, registers and websites, including any filters and limits used.	p5
Selection process	8	Specify the methods used to decide whether a study met the inclusion criteria of the review, including how many reviewers screened each record and each report retrieved, whether they worked independently, and if applicable, details of automation tools used in the process.	p6
Data collection process	9	Specify the methods used to collect data from reports, including how many reviewers collected data from each report, whether they worked independently, any processes for obtaining or confirming data from study investigators, and if applicable, details of automation tools used in the process.	p6
Data items	10a	List and define all outcomes for which data were sought. Specify whether all results that were compatible with each outcome domain in each study were sought (e.g. for all measures, time points, analyses), and if not, the methods used to decide which results to collect.	p5
	10b	List and define all other variables for which data were sought (e.g. participant and intervention characteristics, funding sources). Describe any assumptions made about any missing or unclear information.	p5
Study risk of bias assessment	11	Specify the methods used to assess risk of bias in the included studies, including details of the tool(s) used, how many reviewers assessed each study and whether they worked independently, and if applicable, details of automation tools used in the process.	p6
Effect measures	12	Specify for each outcome the effect measure(s) (e.g. risk ratio, mean difference) used in the synthesis or presentation of results.	p6
Synthesis methods	13a	Describe the processes used to decide which studies were eligible for each synthesis (e.g. tabulating the study intervention characteristics and comparing against the planned groups for each synthesis (item #5)).	P6
	13b	Describe any methods required to prepare the data for presentation or synthesis, such as handling of missing summary statistics, or data conversions.	P6
	13c	Describe any methods used to tabulate or visually display results of individual studies and syntheses.	P6
	13d	Describe any methods used to synthesize results and provide a rationale for the choice(s). If meta-analysis was performed, describe the model(s), method(s) to identify the presence and extent of statistical heterogeneity, and software package(s) used.	P6
	13e	Describe any methods used to explore possible causes of heterogeneity among study results (e.g. subgroup analysis, meta-regression).	p6
	13f	Describe any sensitivity analyses conducted to assess robustness of the synthesized results.	
Reporting bias assessment	14	Describe any methods used to assess risk of bias due to missing results in a synthesis (arising from reporting biases).	p6-7
Certainty assessment	15	Describe any methods used to assess certainty (or confidence) in the body of evidence for an outcome.	P7

For peer review only

PRISMA 2020 Checklist

Section and Topic	Item #	Checklist item	Location where item is reported
**RESULTS**			
Study selection	16a	Describe the results of the search and selection process, from the number of records identified in the search to the number of studies included in the review, ideally using a flow diagram.	p7
	16b	Cite studies that might appear to meet the inclusion criteria, but which were excluded, and explain why they were excluded.	supplement
Study characteristics	17	Cite each included study and present its characteristics.	NA
Risk of bias in studies	18	Present assessments of risk of bias for each included study.	NA
Results of individual studies	19	For all outcomes, present, for each study: (a) summary statistics for each group (where appropriate) and (b) an effect estimate and its precision (e.g. confidence/credible interval), ideally using structured tables or plots.	NA
Results of syntheses	20a	For each synthesis, briefly summarise the characteristics and risk of bias among contributing studies.	NA
	20b	Present results of all statistical syntheses conducted. If meta-analysis was done, present for each the summary estimate and its precision (e.g. confidence/credible interval) and measures of statistical heterogeneity. If comparing groups, describe the direction of the effect.	NA
	20c	Present results of all investigations of possible causes of heterogeneity among study results.	NA
	20d	Present results of all sensitivity analyses conducted to assess the robustness of the synthesized results.	NA
Reporting biases	21	Present assessments of risk of bias due to missing results (arising from reporting biases) for each synthesis assessed.	NA
Certainty of evidence	22	Present assessments of certainty (or confidence) in the body of evidence for each outcome assessed.	NA
**DISCUSSION**			
Discussion	23a	Provide a general interpretation of the results in the context of other evidence.	p7
	23b	Discuss any limitations of the evidence included in the review.	p7
	23c	Discuss any limitations of the review processes used.	p7
	23d	Discuss implications of the results for practice, policy, and future research.	p7
**OTHER INFORMATION**			
Registration and protocol	24a	Provide registration information for the review, including register name and registration number, or state that the review was not registered.	p4
	24b	Indicate where the review protocol can be accessed, or state that a protocol was not prepared.	p4
	24c	Describe and explain any amendments to information provided at registration or in the protocol.	p4
Support	25	Describe sources of financial or non-financial support for the review, and the role of the funders or sponsors in the review.	p9
Competing interests	26	Declare any competing interests of review authors.	p9
Availability of data, code and other materials	27	Report which of the following are publicly available and where they can be found: template data collection forms; data extracted from included studies; data used for all analyses; analytic code; any other materials used in the review.	p10-14

From: Page MJ, McKenzie JE, Bossuyt PM, Boutron I, Hoffmann TC, Mulrow CD, et al. The PRISMA 2020 statement: an updated guideline for reporting systematic reviews. BMJ 2021;372:n71. doi: 10.1136/bmj.n71

For more information, visit: http://www.prisma-statement.org/

For peer review only - http://bmjopen.bmj.com/site/about/guidelines.xhtml

Routine antibiotics for infants less than six months of age with growth failure/faltering. A systematic review

Journal:	*BMJ Open*
Manuscript ID	bmjopen-2022-071393.R1
Article Type:	Original research
Date Submitted by the Author:	28-Mar-2023
Complete List of Authors:	Imdad, Aamer; SUNY Upstate Medical University, Pediatrics<br>Chen, Fanny F; Upstate University Hospital Department of Medicine,<br>François, Melissa; SUNY Upstate Medical University,<br>Sana, Momal; Monash Health,<br>Tanner-Smith, Emily; University of Oregon,<br>Smith, Abigail; SUNY Upstate Medical University, Health Science Library<br>Tsistinas, Olivia; SUNY Upstate Medical University<br>Das, Jai K.; Aga Khan University, Division of Women and Child Health<br>Bhutta, Zulfiqar; The Aga Khan University, Division of Women and Child Health; Hospital for Sick Children Research Institute, Global Child Health
**Primary Subject Heading**:	Nutrition and metabolism
Secondary Subject Heading:	Gastroenterology and hepatology, Global health, Infectious diseases, Paediatrics
Keywords:	Nutrition < TROPICAL MEDICINE, INFECTIOUS DISEASES, PUBLIC HEALTH

SCHOLARONE™
Manuscripts

I, the Submitting Author has the right to grant and does grant on behalf of all authors of the Work (as defined in the below author licence), an exclusive licence and/or a non-exclusive licence for contributions from authors who are: i) UK Crown employees; ii) where BMJ has agreed a CC-BY licence shall apply, and/or iii) in accordance with the terms applicable for US Federal Government officers or employees acting as part of their official duties; on a worldwide, perpetual, irrevocable, royalty-free basis to BMJ Publishing Group Ltd ("BMJ") its licensees and where the relevant Journal is co-owned by BMJ to the co-owners of the Journal, to publish the Work in this journal and any other BMJ products and to exploit all rights, as set out in our licence.

The Submitting Author accepts and understands that any supply made under these terms is made by BMJ to the Submitting Author unless you are acting as an employee on behalf of your employer or a postgraduate student of an affiliated institution which is paying any applicable article publishing charge ("APC") for Open Access articles. Where the Submitting Author wishes to make the Work available on an Open Access basis (and intends to pay the relevant APC), the terms of reuse of such Open Access shall be governed by a Creative Commons licence – details of these licences and which Creative Commons licence will apply to this Work are set out in our licence referred to above.

Other than as permitted in any relevant BMJ Author's Self Archiving Policies, I confirm this Work has not been accepted for publication elsewhere, is not being considered for publication elsewhere and does not duplicate material already published. I confirm all authors consent to publication of this Work and authorise the granting of this licence.

For peer review only

Routine antibiotics for infants less than six months of age with growth failure/faltering. A systematic review

Aamer Imdad, Fanny F Chen, Melissa François, Momal Sana, Emily Tanner-Smith, Abigail Smith, Olivia Tsistinas, Jai Das, Zulfiqar Ahmed Bhutta

Authors' information
Corresponding Author
Aamer Imdad, MBBS, MPH
Assistant Professor of Pediatrics
Division of Pediatric Gastroenterology, Hepatology, and Nutrition
SUNY Upstate Medical University, Syracuse, NY
725 Irving Street, Syracuse, NY 13210
Email: imdada@upstate.edu
Phone: 315-464-8444
Fax: 315-464-8443

Fanny Chen
SUNY Upstate Medical University
ChenF@upstate.edu

Melissa Francois
SUNY Upstate Medical University
FrancoMe@upstate.edu

Momal Sana
Quaid e Azam Medical College
momalsana42@gmail.com

Emily Tanner-Smith
University of Oregon
etanners@uoregon.edu

Abigail Smith
SUNY Upstate Medical University
SmithAb@upstate.edu

Olivia Tsistinas
SUNY Upstate Medical University
TsistiJO@upstate.edu

Jai Das
Aga Khan University
Jai.das@aku.edu

Zulfiqar Ahmed Bhutta
Institute for Global Health & Development, The Aga Khan University

For peer review only

zulfiqar.bhutta@aku.edu

Key Words: Antibiotics, wasting, malnutrition, growth failure

For peer review only

Abstract

Objective: This systematic review commissioned by WHO aimed to synthesize evidence from current literature on the effects of systematically given, routine use of antibiotics for infants under six months of age with growth failure/faltering.

Settings: Low and Middle-income countries

Participants: The study population was infants less than six months of age with growth failure/faltering.

Intervention: The intervention group was infants who received no antibiotics or antibiotics other than those recommended in 2013 guidelines by WHO to treat childhood severe acute malnutrition. The comparison group was infants who received antibiotics according to the aforementioned guidelines.

Primary and Secondary outcomes: The primary outcome was all-cause mortality, and secondary outcomes: clinical deterioration, antimicrobial resistance, recovery from co-morbidity, adverse events, markers of intestinal inflammation, markers of systemic inflammation, hospital-acquired infections, and non-response. The Grading of Recommendations Assessment, Development, and Evaluation (GRADE) approach was considered to report the overall evidence quality for an outcome.

Results: We screened 5137 titles and abstracts and reviewed the full text of 157 studies. None of the studies from the literature search qualified to answer the question for this systematic review.

Conclusions: There is a paucity of evidence on the routine use of antibiotics for the treatment of malnutrition in infants less than six months of age. Future studies with adequate sample sizes are needed to assess the potential risks and benefits of antibiotics in malnourished infants under six months of age.

Systematic Review registration: This study was registered at the PROSPERO website (ID=CRD42021277073)

Strengths and Limitations

- This World Health Organization funded systematic review was conducted by following the standard methods of Cochrane Collaboration.
- Even though data were available for the use of antibiotics in severely malnourished children 6-59 months of age, no randomized trials were found in infants less than six months of age
- The work was limited due to a lack of studies in this age group. Future studies with large sample size are needed to assess the efficacy and safety of antibiotics in malnourished infants less than six months of age.

For peer review only

Main Manuscript

Introduction

The World Health Organization (WHO) and the United Nations Children's Fund (UNICEF) estimate that nearly 14 million children suffer from severe wasting (low weight for height) worldwide[1]. Infants less than six months of age are particularly vulnerable to the effects of inadequate nutrition. Higher mortality rates secondary to growth failure are seen in this age group compared to older infants and children[2,3]. Despite excess mortality risk and increasing prevalence of wasting in this population, limited studies exist to guide the management of young infants with growth failure and faltering[2-4]. Malnutrition in children increases the risk of severe infections and triples the mortality risk from pneumonia, measles, or diarrhea[5]. Therefore, the current practice for children six months to 5 years of age with wasting is to prescribe routine antibiotics when they get into a nutrition program, inpatient or outpatient[5]. However, current recommendations state that the same general medical care should be used for infants with SAM who are less than six months of age as infants above six months of age, even though there is limited evidence to support this recommendation[2]. Furthermore, even though antibiotics are effective in children 6-59 months of age with SAM, this practice in infants has the potential to harm due to recently identified risks of antibiotic use in infancy, including the diminishment of infant gut microbiome[6], future development of obesity, allergic disorders[7], and autoimmune disorders[8]. The urgency in more targeted management guidelines is further underscored by the physiological differences in renal and gastrointestinal function in infants compared to older children[8]. The WHO recently started the guideline development process for preventing and treating wasting in children, including growth failure/faltering in infants under six months. This systematic review aimed to synthesize evidence from current literature on the effect of systematically given, routine use of antibiotics for infants less than six months of age with growth failure/faltering.

OBJECTIVE

Primary Objective: In infants <6 months with growth failure/faltering, what are the effects of no routine antibiotics or different approaches (e.g., types of antibiotics, doses) compared to routine antibiotics following treatment protocols in 2013 WHO guidelines[2] on the morbidity and mortality outcomes?

METHODS AND ANALYSIS

This systematic review was conducted according to methods described in Cochrane Handbook[9] and reported using PRISMA guidelines 2020[10]. We registered the protocol on the PROSPERO website (ID=CRD42021277073).

Types of studies: We considered both individual and cluster randomized trials. We also considered non-randomized trials and cohort studies with a controlled arm. We excluded case-control studies, case reports, case series, and commentaries.

Population

The population of interest was infants under six months of age with growth failure/faltering. We considered the author's definitions because this age group has no standard definition of growth failure/faltering. We considered studies irrespective of whether they were done in community or hospital settings. We considered studies that included infants infected with HIV. We considered studies with low birth weight or preterm infants; however, we excluded studies on infants admitted to neonatal intensive care units. We excluded studies that only included infants with congenital anomalies.

For peer review only

Intervention
We considered all antibiotic treatments given systemically, such as amoxicillin, Augmentin, cephalosporins, and macrolides. We considered studies irrespective of dosage, frequency, duration, or route of administration; however, topical application of antibiotics was not considered. We considered studies if antibiotics were given empirically at the time of diagnosis of growth failure or faltering, irrespective of the indication, e.g., to treat an infection. We excluded studies where antibiotics were given to prevent wasting in otherwise healthy infants, specifically non-malnourished children or infants with no growth failure/faltering. We excluded studies where antibiotics were given for other reasons, such as suspected serious bacterial infections in otherwise healthy infants, specifically non-malnourished children or infants with no growth failure/faltering.

Comparison
The comparison group was routine antibiotics following treatment protocols detailed in the 2013 WHO guideline[2].

Outcomes
-Mortality (Dichotomous outcome)
-Clinical deterioration [Dichotomous outcome, defined by the development of any danger signs (obstructed breathing, respiratory distress, cyanosis, shock, severe anemia, convulsion, severe dehydration, profuse watery diarrhea, intractable vomiting, and/or impaired consciousness)]
-Recovery from co-morbidity (Dichotomous outcome)
-Markers of intestinal inflammation-fecal calprotectin (Continuous outcome)
-Markers of systemic inflammation-serum C reactive protein (Continuous outcome)
-Hospital-acquired infections (Dichotomous outcome)
-Non-response (e.g., not achieving recovery within four months of initiating treatment) (Dichotomous outcome)

All the primary analyses were considered at the longest follow-up. For the outcome of recovery from morbidity, the recovery could be recovery from diarrhea, pneumonia, measles, etc.

LITERATURE SEARCH

We conducted systematic electronic queries using key terms in multiple databases, including MEDLINE via PubMed, EMBASE, Web of Science, CINAHL, Scopus, LILACS, WHO Global Index Medicus, and BIOSIS Previews. There were no search restrictions on outcomes, publication year, publication status, or publication language. The search strategies for different databases are available in Appendix 1. The references of formerly published reviews and recently published studies were examined for potential inclusion. We also used The Cochrane Central Register for Controlled Trials and ISRCTN registry to identify studies currently underway. We also searched the websites of pertinent international agencies such as the WHO (including WHO's Reproductive Health Library, electronic Library of Evidence of Nutrition Actions and Global database on the Implementation of Nutrition Action), UNICEF, Global Alliance for Improved Nutrition, International Food Policy Research Institute, International Initiative for Impact Evaluation, Nutrition International, World Bank, USAID and affiliates (e.g., FANTA, SPRING), and the World Food Programme. We searched the abstracts of major conferences, such as annual pediatric academic society meetings. Finally, we used the citation tracking function of the included studies in PubMed to look for any other eligible studies.

DATA EXTRACTION AND SYNTHESIS

For peer review only

Selection of studies

Studies identified during the literature search were collected in an electronic reference manager EndNote[11] literature, and duplicated studies were removed. At least two authors screened study titles and abstracts to assess potential eligibility. Studies selected during this initial phase underwent a full-text review by two authors. The software Covidence[12] was used during the screening process. Disagreements were resolved by discussion, and the senior author on the team assisted as needed.

Data extraction

We planned to extract the data for study region/country, study year, study type, intervention exposure (dose, duration, frequency), comparison, outcomes, population characteristics detailed in subgroup analysis, and risk of bias. We also planned to extract information on the intervention's feasibility, acceptability, equity, and resource use and reported these data in separate tables. We planned to remove raw values for the number of events in the intervention and control group in case of dichotomous outcomes. To avoid reviewer bias, we decided a priori the order of preference for extracting outcomes when data were available in several formats.

Studies with missing data

If a study was only available in an abstract, we contacted the authors for full text. If the full text could not be obtained from any sources, we considered the abstract if sufficient details of the study design and outcomes were available. We attempted to find the protocol of each potentially included study to assess the details of the methods. If the study protocol was not publicly available, we contacted the authors for the same. If the randomized trial results were published in more than one report, we considered all the publications related to that study as one study.

Assessment of risk of bias in included studies

We aimed to evaluate the risk of bias from randomized controlled trials with the Cochrane risk of bias (ROB 2.0)[13]. The risk of bias assessment according to ROB-2 is done for each outcome, not for a particular study[13].

Data Synthesis

We planned to report the review findings both qualitatively and quantitatively. A narrative synthesis was considered to report all included studies' characteristics and results. A random-effects meta-analysis was planned when at least two studies possessed sufficient clinical and methodological uniformity for synthesis. The software RevMan was considered for statistical analysis[14]. We planned to assess the dichotomous outcomes using relative risk (R.R.) effect sizes and continuous outcomes with a mean difference and report with 95 % C.I.s.

Assessment of heterogeneity

We aimed to analyze statistical heterogeneity in the pooled data using Tau^2, χ^2, and I^2 statistics. We also aimed to assess statistical heterogeneity through visual inspection of forest plots, utilizing the χ^2 test (assessing the p-value) and calculating the Tau^2 and I^2 statistics. We considered it significant statistical heterogeneity when the p-value was less than 0.1, the I^2 value exceeded 50%, and the inspection of forest plots showed substantial variability in the effect of the intervention. Finally, we considered subgroup analysis to identify reasons for eligible statistical heterogeneity.

Assessment of reporting bias

For peer review only

We aimed to assess the publication bias of small studies using funnel plots and regression tests for funnel plot asymmetry when the meta-analysis included at least ten studies.

Subgroup analysis and investigation of heterogeneity

We planned the following subgroup analysis; however, none were possible due to a lack of studies.

- By different types/definitions of growth failure/faltering
- Age at presentation [newborn (0-28 days), 1-3 months, 4-6 months]
- Gestational age: Preterm birth (< 37 weeks) vs. full-term birth (> 37 weeks)
- Birth weight: Low birth weight (<2500 g) vs. Normal birth weight (> 2500 g)
- HIV exposure: Studies with Participants exposed to HIV VS. Studies with no HIV exposure
- Presentation: Participants with Oedema vs. Patricians with no edema
- Co-morbidities: With or without co-morbidities
- Nutrition: Babies Breastfeeding or non-breastfed babies
- Location of the treatment: inpatient or outpatient/community
- Dose of antibiotics
- Duration of antibiotics: 7 days vs. > 7 days
- Type of antibiotics

Sensitivity analysis

We planned to complete sensitivity analysis by the use of the model for meta-analysis.

Rating of overall quality of evidence

The Grading of Recommendations Assessment, Development, and Evaluation (GRADE) approach was considered to evaluate the overall certainty of evidence using the software GRADEpro[15]. The GRADE approach is a comprehensive framework used to assess the overall certainty of the evidence for an outcome utilizing study characteristics such as study design, inconsistency, indirectness of evidence, risk of bias, publication bias, and imprecision estimates.

Patient and public involvement

Patients and/or the public were not involved in this research's design, conduct, reporting, or dissemination plans.

RESULTS

Literature search

We screened 5137 titles and abstracts; 157 eligible studies were screened for full-text reviews. None of the available studies qualified for inclusion in this review. Figure 1 shows the results of the literature search. The reasons for exclusions are available in the table of excluded studies (supplementary document 1). In summary, 85 studies were excluded because of wrong study design, 55 were excluded due to ineligible patient population, seven were excluded because of the ineligible comparison group, and eight were excluded because of ineligible intervention, two studies were excluded because of wrong indication.

DISCUSSION

This systematic review aimed to assess the effect of routine use of antibiotics for the treatment of malnutrition in infants less than six months of age. Even though we reviewed more than 4000 titles and about 157 full-text studies, none of the studies qualified for inclusion in this review. The three key

For peer review only

reasons to publish this work are to document our study question, to report the methodology transparently in enough detail so that it can be replicated in the future as needed, and to highlight the key gaps in research so that the research investigator can design studies for infants < 6 months with malnutrition as the mortality risk is the highest due to malnutrition in this age group.

We followed the methodology of the Cochrane Collaboration to conduct this systematic review. A detailed protocol was prepared before the review process and was externally reviewed and registered publicly on PROSPERO's international database of prospective systematic reviews. The title and abstracts of the studies were screened independently by two study authors. We planned to study the risk of bias using the revised Cochrane risk of bias 2.0 tool for each outcome within a study rather than determining the risk of bias based on all outcomes for a particular study. However, no eligible study was found to answer the clinical question in this review.

We noted two studies of great interest to our population of interest[16,17]. These studies were excluded because of the lack of a comparison group treated with antibiotics according to the current guideline of the World Health Organization for treating wasting.[2] Both the studies included participants that fall in the age range (i.e., < 6 months) considered for this review; however, they also included participants beyond six months of age[16,17]. Both studies were randomized, double-blind, placebo-controlled trials. One study was conducted in Kenya[16] and another study was a multicounty trial[17]. The study from Kenya [16] used co-trimoxazole in the community settings to prevent mortality in severely malnourished children 2-59 months of age after being treated according to WHO protocol (both groups received antibiotics according to WHO protocol during the stabilization period). Daily Co-trimoxazole after initial treatment for severe malnutrition did not prevent mortality in children 2-59 months of age (Hazard ratio 0·90, 95% CI 0·71-1·16). The results for infants two months to 5 months of age were similar to overall results[16]. The other study used biannual azithromycin in children 1-59 months of age irrespective of nutritional status[17] and showed a 13.5 % reduction in mortality in the azithromycin group compared to placebo. A subgroup analysis for malnourished children showed similar results[18]. Two other studies could have been included; however, both included both nourished and malnourished participants, and we could not obtain the randomized data for our population of interest[19,20]. We also identified a recently completed trial[21] and requested the data for inclusion in this review; however, study investigators were submitting their results to a peer review journal and are not yet willing to share those data.

Implications for research

Future randomized studies, with adequate sample size of infants under six months with malnutrition and/or growth faltering, are needed to confirm the therapeutic effect of antibiotic treatment observed in children 6-59 months of age[2]. Additional data are also required to assess the appropriate antibiotic, dose, route, frequency, and duration of antibiotic treatment. The safety profile regarding acute reactions, such as allergic reactions, gastrointestinal disturbance, etc., and long-term effects, such as effects on the gut microbiome and antibiotic resistance, also need further investigation.

CONCLUSIONS

There is a paucity of evidence to assess the effect of routine use of antibiotics in infants less than six months of age with malnutrition. Future studies with large sample sizes are needed to evaluate the potential risks and benefits of antibiotics in malnourished children under six months of age.

For peer review only

Amendments
We conducted literature searches, screening of titles, selection of studies, data extraction, and analysis according to the plan outlined in the protocol.

Ethics statements
Patient consent for publication
This study does not involve human participants, and ethical approval was not required

Data Sharing Statement
We provide the list of excluded studies and our search strategies. The results of the literature search are available on request.

Funding
This work is funded by World Health Organization grant no 202725572. WHO also provided technical support for this work.

Acknowledgments
We are very thankful to Allison Daniel, Jaden Bendabenda and, Zita Weise Prinzo, Kirrily De Polnay for their input in improving this review.

Competing Interest
There are no conflicts of interest identified in the conduction of this research.

Author Contributions: Conceptualization, A.I..; methodology, A.I., F.C.; validation, A.I., F.C., M.F., M.S..; formal analysis, A.I., F.C.; resources, E.TS., A.S., O.T.; data curation, F.C., M.F., M.S.; writing, A.I., F.C.; review and editing, A.I., F.C., J.D., Z.B.. All authors have read and agreed to the published version of the manuscript.

References

1. United Nations Children's Fund (UNICEF), World Health Organization, International Bank for Reconstruction and Development/The World Bank. Levels and trends in child malnutrition: Key Findings of the 2020 Edition of the Joint Child Malnutrition Estimates. Geneva: World Health Organization. Licence: CC BY-NC-SA 3.0 IGO. 2020.
2. WHO. Guideline: Updates on the management of severe acute malnutrition in infants and children. Geneva: World Health Organization. 2013.

For peer review only

3. Kerac M, Blencowe H, Grijalva-Eternod C, et al. Prevalence of wasting among under 6-month-old infants in developing countries and implications of new case definitions using WHO growth standards: a secondary data analysis. *Arch Dis Child.* 2011;96(11):1008-1013.
4. Patwari AK, Kumar S, Beard J. Undernutrition among infants less than 6 months of age: an underestimated public health problem in India. *Matern Child Nutr.* 2015;11(1):119-126.
5. Alcoba G, Kerac M, Breysse S, et al. Do children with uncomplicated severe acute malnutrition need antibiotics? A systematic review and meta-analysis. *PLoS One.* 2013;8(1):e53184.
6. Stam J, van Stuijvenberg M, Gruber C, et al. Antibiotic use in infants in the first year of life in five European countries. *Acta Paediatr.* 2012;101(9):929-934.
7. Droste JH, Wieringa MH, Weyler JJ, Nelen VJ, Vermeire PA, Van Bever HP. Does the use of antibiotics in early childhood increase the risk of asthma and allergic disease? *Clin Exp Allergy.* 2000;30(11):1547-1553.
8. Trasande L, Blustein J, Liu M, Corwin E, Cox LM, Blaser MJ. Infant antibiotic exposures and early-life body mass. *Int J Obes (Lond).* 2013;37(1):16-23.
9. Higgins JPT TJ, Chandler J, Cumpston M, Li T, Page MJ, Welch VA (editors). Cochrane Handbook for Systematic Reviews of Interventions version 6.2 Available from www.training.cochrane.org/handbook. 2021.
10. Page MJ, McKenzie JE, Bossuyt PM, et al. The PRISMA 2020 statement: an updated guideline for reporting systematic reviews. *BMJ.* 2021;372:n71.
11. *EndNote* [computer program]. Version EndNote X9. Philadelphia, PA: Clarivate; 2013.
12. *Covidence systematic review software* [computer program]. Melbourne, Australia: www.covidence.org; 2021.
13. Sterne JAC, Savovic J, Page MJ, et al. RoB 2: a revised tool for assessing risk of bias in randomised trials. *BMJ.* 2019;366:l4898.
14. *Review Manager (RevMan)* [computer program]. Version 5.3. Copenhagen: The Nordic Cochrane Center TCC; 2014.
15. *GRADEpro GDT: GRADEpro guideline development tool* [computer program]. McMaster University: Evidence Prime, Inc; 2020.
16. Berkley JA, Ngari M, Thitiri J, et al. Daily co-trimoxazole prophylaxis to prevent mortality in children with complicated severe acute malnutrition: a multicentre, double-blind, randomised placebo-controlled trial. *Lancet Glob Health.* 2016;4(7):e464-473.
17. Keenan JD, Bailey RL, West SK, et al. Azithromycin to Reduce Childhood Mortality in Sub-Saharan Africa. *N Engl J Med.* 2018;378(17):1583-1592.
18. O'Brien KS, Arzika AM, Maliki R, et al. Biannual azithromycin distribution and child mortality among malnourished children: A subgroup analysis of the MORDOR cluster-randomized trial in Niger. *PLoS Med.* 2020;17(9):e1003285.
19. Gore-Langton GR, Cairns M, Compaore YD, et al. Effect of adding azithromycin to the antimalarials used for seasonal malaria chemoprevention on the nutritional status of African children. *Trop Med Int Health.* 2020;25(6):740-750.
20. Pavlinac PB, Singa BO, Tickell KD, et al. Azithromycin for the prevention of rehospitalisation and death among Kenyan children being discharged from hospital: a

For peer review only

double-blind, placebo-controlled, randomised controlled trial. *Lancet Glob Health.* 2021;9(11):e1569-e1578.

21. Muhammad A SY, Nisar MI, Baloch B, Yazdani AT, Yazdani N, Jehan F. . Nutritional support for lactating women with or without azithromycin for infants compared to breastfeeding counseling alone in improving the 6-month growth outcomes among infants of peri-urban slums in Karachi, Pakistan-the protocol for a multiarm assessor-blinded randomized controlled trial *Trials.* 2020;1(21):756.

Figure Legend:
Figure 1: PRISMA flow diagram

For peer review only

Figure 1: PRISMA flow diagram

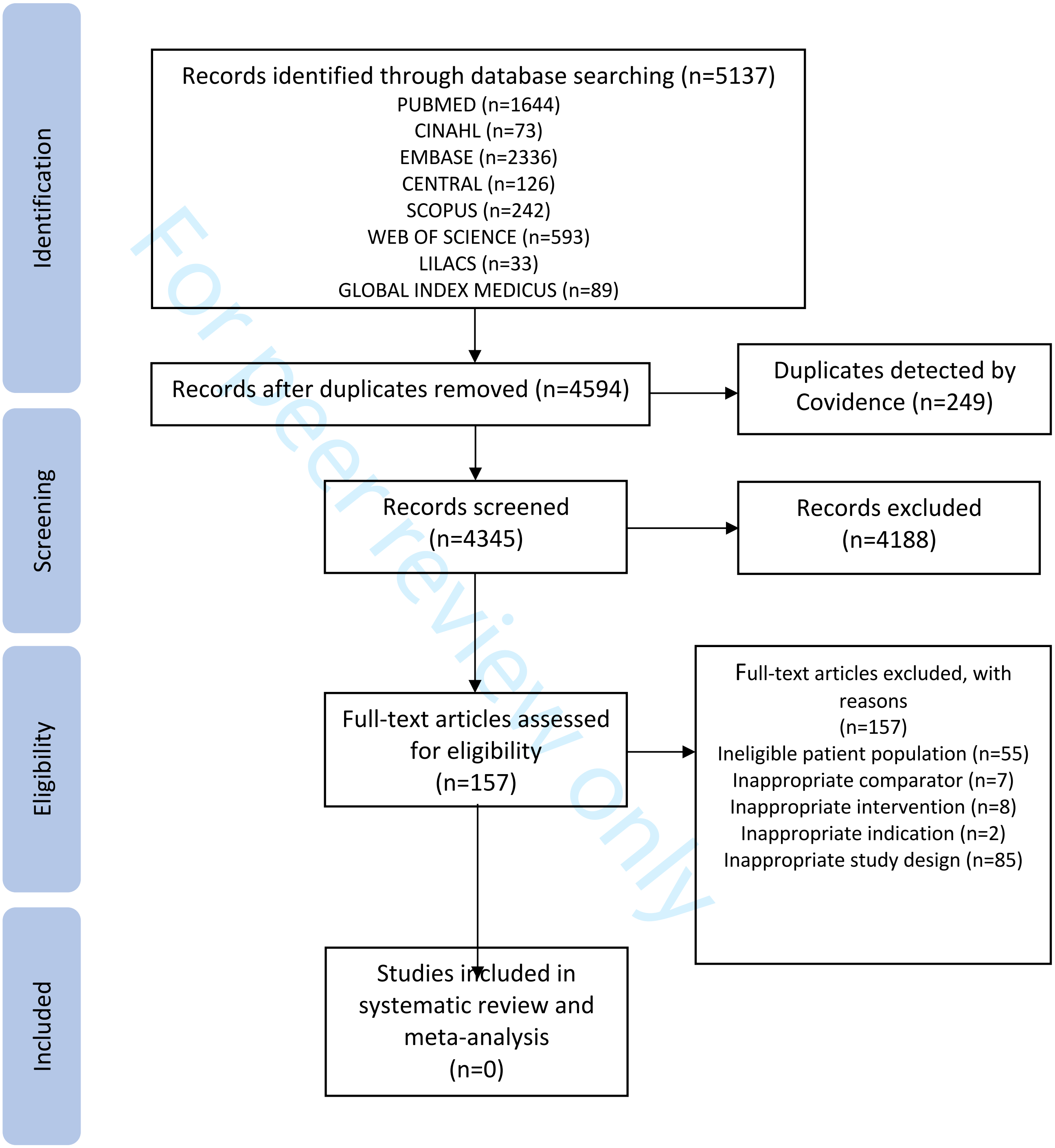

For peer review only

Appendix 1

Search Strategies for Growth Failure AND Antibiotics

PubMed

(("Infant"[MeSH Terms] OR "Child"[MeSH Terms:noexp] OR "Premature Birth"[MeSH Terms] OR ("infant*"[Title/Abstract] OR "infancy"[Title/Abstract] OR "baby"[Title/Abstract] OR "babies"[Title/Abstract] OR "neonat*"[Title/Abstract] OR "neo nat*"[Title/Abstract] OR "newborn*"[Title/Abstract] OR "new born*"[Title/Abstract] OR "newly born*"[Title/Abstract] OR "preterm"[Title/Abstract] OR "preterms"[Title/Abstract] OR "pre term"[Title/Abstract] OR "pre terms"[Title/Abstract] OR "lbw"[Title/Abstract] OR "vlbw"[Title/Abstract] OR "elbw"[Title/Abstract] OR "child*"[Title/Abstract] OR "youth"[Title/Abstract] OR "juvenile*"[Title/Abstract]) OR ("premature*"[Title/Abstract] AND ("birth*"[Title/Abstract] OR "born"[Title/Abstract] OR "deliver*"[Title/Abstract])) OR ("low"[Title/Abstract] AND ("birthweight*"[Title/Abstract] OR "birth weight*"[Title/Abstract]))) AND ("Malnutrition"[MeSH Terms] OR "Failure to Thrive"[MeSH Terms] OR "Growth Disorders"[MeSH Terms:noexp] OR "Severe Acute Malnutrition"[MeSH Terms] OR "Wasting Syndrome"[MeSH Terms:noexp] OR "Infant Nutrition Disorders"[MeSH Terms:noexp] OR ("growth failure*"[Title/Abstract] OR "growth falter*"[Title/Abstract] OR "slow growth"[Title/Abstract] OR "slowed growth"[Title/Abstract] OR "Malnutrition"[Title/Abstract] OR "malnourish*"[Title/Abstract] OR "Severe Acute Malnutrition"[Title/Abstract] OR "SAM"[Title/Abstract] OR "wasting"[Title/Abstract] OR "wasted"[Title/Abstract] OR "growth restrict*"[Title/Abstract] OR "growth retard*"[Title/Abstract] OR "Failure to Thrive"[Title/Abstract]

For peer review only

OR "FTT"[Title/Abstract] OR "growth disorder*"[Title/Abstract] OR "growth arrest*"[Title/Abstract] OR "growth deficien*"[Title/Abstract] OR "growth disturb*"[Title/Abstract])) AND ("Anti-Bacterial Agents"[Pharmacological Action] OR "Anti-Bacterial Agents"[MeSH Terms] OR ("antibiotic*"[Title/Abstract] OR "anti bacterial agent*"[Title/Abstract] OR "antibacterial*"[Title/Abstract] OR "anti bacterial*"[Title/Abstract] OR "bactericid*"[Title/Abstract] OR "bacteriocid*"[Title/Abstract] OR "anti mycobacterial*"[Title/Abstract] OR "antimycobacterial*"[Title/Abstract]))) NOT ("Animals"[MeSH Terms] NOT ("Animals"[MeSH Terms] AND "Humans"[MeSH Terms]))

CINAHL

S1 (MH "Infant+")
S2 (MH "Child")
S3 (MH "Childbirth, Premature")
S4 TI (infant* OR infancy OR baby OR babies OR neonat* OR "neo nat*" OR newborn* OR "new born*" OR "newly born*" OR preterm OR preterms OR "pre term" OR "pre terms" OR lbw OR vlbw OR elbw OR child* OR youth OR juvenile*)
S5 AB (infant* OR infancy OR baby OR babies OR neonat* OR "neo nat*" OR newborn* OR "new born*" OR "newly born*" OR preterm OR preterms OR "pre term" OR "pre terms" OR lbw OR vlbw OR elbw OR child* OR youth OR juvenile*)
S6 TI (premature* N2 (birth* OR born OR deliver*))
S7 AB (premature* N2 (birth* OR born OR deliver*))
S8 TI (low N2 (birthweight* OR "birth weight*"))
S9 AB (low N2 (birthweight* OR "birth weight*"))
S10 S1 OR S2 OR S3 OR S4 OR S5 OR S6 OR S7 OR S8 OR S9

S11 (MH "Malnutrition")
S12 (MH "Failure to Thrive")

For peer review only

S13 (MH "Growth Disorders")
S14 (MH "Wasting Syndrome")
S15 (MH “Infant Nutrition Disorders”)
S16 TI ((grow*) N2 (fail* OR falter* OR slow* OR restrict* OR retard* OR deficien* OR arrest* OR
disturb* OR disorder*))
S17 AB ((grow*) N2 (fail* OR falter* OR slow* OR restrict* OR retard* OR deficien* OR arrest* OR
disturb* OR disorder*))
S18 TI ((fail*) W2 (thrive))
S19 AB ((fail*) W2 (thrive))
S20 TI (Malnutrition OR malnourish* OR "Severe Acute Malnutrition" OR SAM OR wasting OR
wasted OR FTT)
S21 AB (Malnutrition OR malnourish* OR "Severe Acute Malnutrition" OR SAM OR wasting OR
wasted OR FTT)
S22 S11 OR S12 OR S13 OR S14 OR S15 OR S16 OR S17 OR S18 OR S19 OR S20 OR S21
S23 (MH "Antibiotics+")
S24 TI (antibiotic* OR "anti bacterial*" OR "antibacterial*" OR bactericid*OR bacteriocid* OR "anti mycobacterial*" OR "antimycobacterial*")
S25 AB (antibiotic* OR "anti bacterial*" OR "antibacterial*" OR bactericid* OR bacteriocid* OR "anti mycobacterial*" OR "antimycobacterial*")
S26 S23 OR S24 OR S25
S27 (MH "Animals") NOT ((MH "Animals") AND (MH "Human"))
S28 S10 AND S22 AND S26
S29 S28 NOT S27
S30 S28 NOT S27 Exclude Medline Records

Embase

#1'infant'/exp OR 'newborn'/exp OR 'prematurity'/exp OR 'low birth weight'/exp OR 'very low birth

For peer review only

weight'/exp OR 'extremely low birth weight'/exp OR
'juvenile'/de OR 'child'/de
#2 infant*:ti,ab OR infancy:ti,ab OR baby:ti,ab OR babies:ti,ab OR neonat*:ti,ab
OR 'neo nat*':ti,ab
OR newborn*:ti,ab OR 'new born*':ti,ab OR 'newly
born*':ti,ab OR preterm:ti,ab OR preterms:ti,ab
OR 'pre term':ti,ab OR 'pre terms':ti,ab OR lbw:ti,ab OR
vlbw:ti,ab OR elbw:ti,ab OR child*:ti,ab OR
youth:ti,ab OR juvenile*:ti,ab
#3 (premature* NEAR/2 (birth* OR born OR deliver*)):ti,ab
#4 (low NEAR/2 (birthweight* OR 'birth weight*')):ti,ab
#5 #1 OR #2 OR #3 OR #4

#6 'malnutrition'/exp OR 'failure to thrive'/exp OR
'growth disorder'/de OR 'wasting syndrome'/de
OR 'growth retardation'/de
#7(grow* NEAR/2 (fail* OR falter* OR slow* OR restrict* OR retard* OR deficien*
OR arrest* OR
disturb* OR disorder*)):ti,ab
#8 (fail* NEXT/2 thrive):ti,ab
#9 malnutrition:ti,ab OR malnourish*:ti,ab OR 'severe acute
malnutrition':ti,ab OR sam:ti,ab OR
wasting:ti,ab OR wasted:ti,ab OR ftt:ti,ab
#10 #6 OR #7 OR #8 OR #9
#11'antibiotic agent'/exp OR 'bactericide'/de OR
'antimycobacterial agent'/de
#12 antibiotic*:ti,ab OR 'anti bacterial*':ti,ab OR
'antibacterial*':ti,ab OR bactericid*:ti,ab OR
bacteriocid*:ti,ab OR antimycobacterial*:ti,ab OR 'anti
mycobacterial*':ti,ab
#13 #11 OR #12
#14 #5 AND #10 AND #13
#15 #14 NOT ('animals'/exp NOT 'humans'/exp)
#16 #15 NOT [medline]/lim

CENTRAL

#1 MeSH descriptor: [Infant] explode all trees
#2 MeSH descriptor: [Child] this term only

For peer review only

#3 MeSH descriptor: [Premature Birth] explode all trees
#4 infant*:ti,ab OR infancy:ti,ab OR baby:ti,ab OR babies:ti,ab OR neonat*:ti,ab OR newborn*:ti,ab
OR preterm:ti,ab OR preterms:ti,ab OR lbw:ti,ab OR vlbw:ti,ab OR elbw:ti,ab OR child*:ti,ab OR
youth:ti,ab OR juvenile*:ti,ab
#5 (neo NEXT nat* OR new NEXT born* OR newly NEXT born* OR pre NEXT term OR pre NEXT
terms):ti,ab
#6 (premature* NEAR/2 (birth* OR born OR deliver*)):ti,ab
#7 (low NEAR/2 (birthweight* OR birth NEXT weight*)):ti,ab
#8 #1 OR #2 OR #3 OR #4 OR #5 OR #6 OR #7
#9 MeSH descriptor: [Malnutrition] explode all trees
#10 MeSH descriptor: [Failure to Thrive] explode all trees
#11 MeSH descriptor: [Growth Disorders] this term only
#12 MeSH descriptor: [Severe Acute Malnutrition] explode all trees
#13 MeSH descriptor: [Wasting Syndrome] this term only
#14 MeSH descriptor: [Infant Nutrition Disorders] this term only
#15 ((grow*) NEAR/2 (fail* OR falter* OR slow* OR restrict* OR retard* OR deficien* OR arrest* OR
disturb* OR disorder*)):ti,ab

#16 ((fail*) NEXT/2 (thrive)):ti,ab
#17 (severe NEXT acute NEXT malnutrition):ti,ab
#18 malnutrition:ti,ab OR malnourish*:ti,ab OR sam:ti,ab OR wasting:ti,ab OR wasted:ti,ab OR
ftt:ti,ab
#19 #9 OR #10 OR #11 OR #12 OR #13 OR #14 OR #15 OR #16 OR #17 OR #18
#20 MeSH descriptor: [Anti-Bacterial Agents] explode all trees
#21 antibiotic*:ti,ab OR antibacterial*:ti,ab OR bactericid*:ti,ab OR bacteriocid*:ti,ab OR
antimycobacterial*:ti,ab
#22 (anti NEXT bacterial*):ti,ab
#23 (anti NEXT mycobacterial*):ti,ab
#24 #20 OR #21 OR #22 OR #23
#25 #8 AND #19 AND #24
#26 MeSH descriptor: [Animals] explode all trees

For peer review only

#27 MeSH descriptor: [Humans] explode all trees
#28 (#26 NOT (#26 AND #27))
#29 #25 NOT #28
#30 "accession number" NEAR pubmed
#31 #29 NOT #30

Scopus

((TITLE-ABS (infant* OR infancy OR baby OR babies OR neonat* OR "neo
nat*" OR
newborn* OR "new born*" OR "newly born*" OR preterm
OR preterms OR "pre term" OR
"pre terms" OR lbw OR vlbw OR elbw OR child* OR youth OR
juvenile*)) OR (TITLE-ABS
(low W/2 (birthweight* OR "birth weight*"))) OR (TITLE-ABS
(premature* W/2 (birth* OR
born OR deliver*)))) AND ((TITLE-ABS ((grow*) W/2 (fail* OR falter* OR
slow* OR
restrict* OR retard* OR deficien* OR arrest* OR disturb* OR disorder*))) OR
(TITLE-ABS (
(fail*) PRE/2 (thrive))) OR (TITLE-ABS (malnutrition OR malnourish* OR
"Severe Acute
Malnutrition" OR sam OR wasting OR wasted OR ftt))) AND (TITLE-ABS
(antibiotic* OR
"anti bacterial*" OR "antibacterial*" OR bactericid* OR
bacteriocid* OR "anti mycobacterial*"
OR "antimycobacterial*")) AND NOT INDEX (medline)

Web of Science

#1 TI=(infant* OR infancy OR baby OR babies OR neonat* OR "neo
nat*" OR newborn* OR
"new born*" OR "newly born*" OR preterm OR preterms
OR "pre term" OR "pre terms" OR
lbw OR vlbw OR elbw OR child* OR youth OR juvenile*)
#2 AB=(infant* OR infancy OR baby OR babies OR neonat* OR "neo
nat*" OR newborn* OR
"new born*" OR "newly born*" OR preterm OR preterms
OR "pre term" OR "pre terms" OR
lbw OR vlbw OR elbw OR child* OR youth OR juvenile*)
#3 TI=(premature* NEAR/2 (birth* OR born OR deliver*))

For peer review only

#4 AB=(premature* NEAR/2 (birth* OR born OR deliver*))
#5 TI=(low NEAR/2 (birthweight* OR "birth weight*"))
#6 AB=(low NEAR/2 (birthweight* OR "birth weight*"))
#7 #6 OR #5 OR #4 OR #3 OR #2 OR #1
#8 TI=((grow*) NEAR/2 (fail* OR falter* OR slow* OR restrict* OR retard* OR deficien* OR arrest*
OR disturb* OR disorder*))
#9 AB=((grow*) NEAR/2 (fail* OR falter* OR slow* OR restrict* OR retard* OR deficien* OR arrest*
OR disturb* OR disorder*))
#10 TI=((fail*) NEAR/2 (thrive))
#11 AB=((fail*) NEAR/2 (thrive))
#12 TI =(Malnutrition OR malnourish* OR "Severe Acute Malnutrition" OR SAM OR wasting OR
wasted OR FTT)
#13 AB=(Malnutrition OR malnourish* OR "Severe Acute Malnutrition" OR SAM OR wasting OR
wasted OR FTT)
#14 #13 OR #12 OR #11 OR #10 OR #9 OR #8
#15 TI=(antibiotic* OR "anti bacterial*" OR "antibacterial*" OR bactericid* OR bacteriocid* OR "anti mycobacterial*" OR "antimycobacterial*")
#16 AB=(antibiotic* OR "anti bacterial*" OR "antibacterial*" OR bactericid* OR bacteriocid* OR "anti mycobacterial*" OR "antimycobacterial*")
#17 #16 OR #15
#18 #17 AND #14 AND #7

LILACS

(mh:(infant)) OR ((mh:(child))) OR ((mh:("Premature Birth"))) OR ((ti:((premature* AND (birth* OR
born OR deliver*))))) OR ((ab:((premature* AND (birth* OR born OR deliver*))))) OR ((ti:((low AND
(birthweight* OR "birth weight*"))))) OR ((ab:((low AND (birthweight* OR "birth weight*"))))) OR
((ti:(infant* OR infancy OR baby OR babies OR neonat* OR "neo nat*" OR newborn* OR "new

For peer review only

born*" OR "newly born*" OR preterm OR preterms OR "pre term" OR "pre terms" OR lbw OR vlbw OR elbw OR child* OR youth OR juvenile*))) OR ((ab:(infant* OR infancy OR baby OR babies OR neonat* OR "neo nat*" OR newborn* OR "new born*" OR "newly born*" OR preterm OR preterms OR "pre term" OR "pre terms" OR lbw OR vlbw OR elbw OR child* OR youth OR juvenile*))) AND ((mh:(malnutrition)) OR ((mh:("Failure to Thrive"))) OR ((mh:("Growth Disorders"))) OR ((mh:("Severe Acute Malnutrition"))) OR ((mh:("Wasting Syndrome"))) OR ((mh:("Infant Nutrition Disorders"))) OR ((ti:("growth failure*" OR "growth falter*" OR "slow growth" OR "slowed growth" OR malnutrition OR malnourish* OR "Severe Acute Malnutrition" OR sam OR wasting OR wasted OR "growth restrict*" OR "growth retard*" OR "Failure to Thrive" OR ftt OR "growth disorder*" OR "growth arrest*" OR "growth deficien*" OR "growth disturb*"))) OR ((ab:("growth failure*" OR "growth falter*" OR "slow growth" OR "slowed growth" OR malnutrition OR malnourish* OR "Severe Acute Malnutrition" OR sam OR wasting OR wasted OR "growth restrict*" OR "growth retard*" OR "Failure to Thrive" OR ftt OR "growth disorder*" OR "growth arrest*" OR "growth deficien*" OR "growth disturb*")))) AND ((mh:("Anti-Bacterial Agents")) OR ((ti:(antibiotic* OR "anti bacterial agent*" OR antibacterial* OR "anti bacterial*" OR "bactericid*" OR "bacteriocid*"

OR "anti mycobacterial*" OR antimycobacterial*))) OR ((ab:(antibiotic* OR "anti bacterial agent*" OR antibacterial* OR "anti bacterial*" OR "bactericid*" OR "bacteriocid*" OR "anti mycobacterial*" OR antimycobacterial*)))) AND NOT ((mh:(animals)) AND NOT ((mh:(animals)) AND

For peer review only

((mh:(humans)))))

Global Index Medicus

(mh:(infant)) OR ((mh:(child))) OR ((mh:("Premature Birth"))) OR ((ti:((premature* AND (birth* OR born OR deliver*))))) OR ((ab:((premature* AND (birth* OR born OR deliver*))))) OR ((ti:((low AND (birthweight* OR "birth weight*"))))) OR ((ab:((low AND (birthweight* OR "birth weight*"))))) OR ((ti:(infant* OR infancy OR baby OR babies OR neonat* OR "neo nat*" OR newborn* OR "new born*" OR "newly born*" OR preterm OR preterms OR "pre term" OR "pre terms" OR lbw OR vlbw OR elbw OR child* OR youth OR juvenile*))) OR ((ab:(infant* OR infancy OR baby OR babies OR neonat* OR "neo nat*" OR newborn* OR "new born*" OR "newly born*" OR preterm OR preterms OR "pre term" OR "pre terms" OR lbw OR vlbw OR elbw OR child* OR youth OR juvenile*))) AND ((mh:(malnutrition)) OR ((mh:("Failure to Thrive"))) OR ((mh:("Growth Disorders"))) OR ((mh:("Severe Acute Malnutrition"))) OR ((mh:("Wasting Syndrome"))) OR ((mh:("Infant Nutrition Disorders"))) OR ((ti:("growth failure*" OR "growth falter*" OR "slow growth" OR "slowed growth" OR malnutrition OR malnourish* OR "Severe Acute Malnutrition" OR sam OR wasting OR wasted OR "growth restrict*" OR "growth retard*" OR "Failure to Thrive" OR ftt OR "growth disorder*" OR "growth arrest*" OR "growth deficien*" OR "growth disturb*"))) OR ((ab:("growth failure*" OR "growth falter*" OR "slow growth" OR "slowed growth" OR malnutrition OR malnourish* OR "Severe Acute Malnutrition" OR sam OR wasting OR wasted OR "growth restrict*" OR "growth retard*" OR "Failure to Thrive" OR ftt OR "growth disorder*" OR "growth arrest*" OR "growth

For peer review only

deficien*" OR "growth disturb*")))) AND ((mh:("Anti-Bacterial Agents")) OR ((ti:(antibiotic* OR "anti bacterial agent*" OR antibacterial* OR "anti bacterial*" OR "bactericid*" OR "bacteriocid*" OR "anti mycobacterial*" OR antimycobacterial*))) OR ((ab:(antibiotic* OR "anti bacterial agent*" OR antibacterial* OR "anti bacterial*" OR "bactericid*" OR "bacteriocid*" OR "anti mycobacterial*" OR antimycobacterial*)))) AND NOT ((mh:(animals)) AND NOT ((mh:(animals)) AND ((mh:(humans))))) AND (collection_gim:("IMSEAR" OR "WPRIM" OR "IMEMR" OR "AIM"))

For peer review only

Antibiotic as an adjunct to treatment of severe acute malnutrition in infants less than six months of age. A protocol for the systematic review

List of Exclusions and Reasons for Exclusions.

Abate 2020[1]	Inappropriate study design and appropriate control was not available
Adam 1954 [2]	Inappropriate Study Design
Adem 2020[3]	Inappropriate Patient Population. Study was a cohort study and only included children 6-59 months of age
Ahmed 1999[4]	Inappropriate Patient Population. Even though all the children were severely malnourished, all of them had diarrhea. Also, the study was not a randomized study.
Ahmed 2001 [5]	Inappropriate Study Design. Authors discuss a protocol for treatment of severe acute malnutrition. The study was a descriptive review
Akbani 1977[6]	Inappropriate study population. Children with SAM who were infected with tuberculosis.
AmorelliGonzaga 1989[7]	Inappropriate study population. Study investigated miomycin and included both well-nourished and malnourished children.
Amza 2013[8]	Inappropriate study population. Study investigated mass azithromycin distribution and included children irrespective of the nutritional status.
Amza 2014[9]	Inappropriate study population. Study investigated mass azithromycin distribution and included children irrespective of the nutritional status.
Angelakis 2014[10]	Inappropriate study population. Study included patients with Q fever endocarditis patients treated with doxycycline and hydroxychloroquine
Ashorn 2018[11]	Inappropriate study population. Study included children with diarrhea, dysentery and malnutrition.
Ashraf 2012[12]	Inappropriate Comparator. Children aged 2-59 months having severe pneumonia with SAM were randomized to day-care or hospital-care. All the children received antibiotics.
Autret 1989[13]	Inappropriate study Design. Study to assess the pharmacokinetics of gentamicin.
Ayelign 2018[14]	Inappropriate study population. Children with urinary tract infections.
Ayiyi 1987[15]	Inappropriate study Design. The study was a systematic review
Aziz 2015[16]	Inappropriate Intervention. Study investigated vitamin K supplementation.
Berkley 2009 [17]	Wrong comparator
Berkley 2016[18]	Inappropriate Comparator. Both the intervention and comparison group were treated according to the WHO

For peer review only - http://bmjopen.bmj.com/site/about/guidelines.xhtml

Antibiotic as an adjunct to treatment of severe acute malnutrition in infants less than six months of age. A protocol for the systematic review

List of Exclusions and Reasons for Exclusions.

	protocol and then randomized to Co-trimoxazole vs. placebo.
Berkowitz 1992[19]	Inappropriate Study Design. Review article.
Bhatnagar 1992[20]	Inappropriate patient population. All the patients had diarrhea. All of them were given gentamicin
Bhatnagar 1996[21]	Inappropriate Study Design. Observation study in children with persistent diarrhea
Birindwa 2020[22]	Inappropriate Study Design. Observational study in children with severe pneumonia
Bleakly 2014[23]	Inappropriate Study Design. Before and after study to assess the effectiveness of a protocol to treat SAM.
Boesen 1953[24]	Inappropriate Indication. Antibiotics were given for treatment of gastroesophageal reflux.
Bolme 1980[25]	Inappropriate Study Design: Pharmacokinetic study for Penicillin in children
Bolme 1988[26]	Inappropriate Study Design: Pharmacokinetic study for streptomycin in children with Tuberculosis.
Bolme 1995[27]	Inappropriate Study Design. Pharmacokinetic study in malnourished children for Penicillin.
Bravo 1982[28]	Inappropriate study design: Study to assess the pharmacokinetics
Bravo 1984[29]	Inappropriate study design: Study to assess the pharmacokinetics
Bredow 1994[30]	Inappropriate Study Design. Observational study
Brodwall 2016[31]	Inappropriate Study Design. Letter to editor
Bruhn 2016[32]	Inappropriate Study Design. Observational study
Brunozi 2019[33]	Inappropriate Study Design. Observational study
Buchanan 1977[34]	Inappropriate Study Design. Letter to editor
Buchanan 1978[35]	Inappropriate Study Design. Observational study
Buchanan 1979 [36]	Inappropriate Study Design. Observational study
Bunn 2009[37]	Inappropriate Study Design. Commentary
Cakir 2012[38]	Inappropriate Patient Population. Children studied leukemia
Caksen 2000[39]	Inappropriate Study Design. Observational study
Caksen 2000[40]	Inappropriate Study Design. Observational study
Cedrato 1974[41]	Inappropriate Study Design. Observational study
Chisti 2015[42]	Inappropriate Patient Population. Children with respiratory symptoms only
Church 2015[43]	Inappropriate Study Design. Observational study
Cochlovius 1953[44]	Inappropriate intervention. Observational study
Dicko 2016[45]	Inappropriate study population. Intervention was given to all the children irrespective of nutritional status at the time of chemoprophylaxis for malaria.
Dieng 2014[46]	Inappropriate Study Design. Observational study
Dubray 2008[47]	Inappropriate study population. Both the study groups received antibiotics and these were given to children 6-59 months of age
Duffau 1990[48]	Inappropriate study population. Children with diarrhea

Antibiotic as an adjunct to treatment of severe acute malnutrition in infants less than six months of age. A protocol for the systematic review

List of Exclusions and Reasons for Exclusions.

Dutta 2006[49]	Inappropriate study design: Observational study
Eriksson 1983[50]	Inappropriate Patient Population. Also, observational study
Eriksson 1988[51]	Inappropriate Patient Population. Also, observational study
Ford 1976[52]	Inappropriate Study Design. Observational study
Friedrich 2016[53]	Inappropriate Study Design. Commentary
Garcia 1974[54]	Inappropriate Study Design. Observational study
Gardner 2011[55]	Inappropriate Study Design. Commentary
Gennaro 2017[56]	Inappropriate Study Design. Commentary
Gensch 1957[57]	Inappropriate Study Design. Observational study
Gernaat 1998[58]	Inappropriate Study Design. Observational study
Ghosh 1995[59]	Inappropriate Patient Population. Children with persistent diarrhea
Girma 2018[60]	Inappropriate Study Design. Observational study
Gore Langton 2020[61]	Inappropriate Patient Population. The antibiotics were given irrespective of nutritional status and study included children beyond six months of age. The comparison group was not appropriate.
Hecht 2015 [62]	Inappropriate Patient Population. The study included children beyond six months of age up to 18 years.
Heikens 1993 [63]	Inappropriate Patient Population, mean age of children at 1.2years.
Heikens 1993 [64]	Inappropriate Comparator, antibiotics compared with receiving health care from community healthcare aids
Hirschhorn 1971 [65]	Inappropriate Study Design, paper is a commentary on the current state of research.
Howard 1967 [66]	Inappropriate Study Design, all infants studied had acute diarrhea
Isaack 1992 [67]	Inappropriate Study Design, investigates nosocomial infections.
Isanaka 2020 [68]	Inappropriate Patient Population. All patients are above 6 months of age.
Islam 2021 [69]	Inappropriate Outcomes. Antibiotic usage was outcome not treatment group. Also all patients have diarrhea.
ISRCTN 2017 [70]	Wrong patient population. Patient age ranged from 2 months to 13 years, unable to obtain subgroup data for under 6 months.
Kabalo 2017 [71]	Inappropriate Patient Population. Patients aged over 6 months.
Lares-Asseff 1999 [72]	Inappropriate Study Design. Study evaluated blood levels for antibiotic not mortality outcome.
Lares-Asseff 2016 [73]	Inappropriate Comparator. Compared different doses of antibiotics to look for toxicity, not mortality.
Lattes 1954 [74]	Inappropriate Study Design and lack of full text.
Lebl 2001 [75]	Inappropriate Patient Population, age range 2 years to 18 years.

For peer review only

Antibiotic as an adjunct to treatment of severe acute malnutrition in infants less than six months of age. A protocol for the systematic review

List of Exclusions and Reasons for Exclusions.

Lelijveld 2021 [76]	Inappropriate Patient Population, age range 6 months to 59 months.
Lepage 1984 [77]	Inappropriate Study Design, patients all have bacterial infection treated with antibiotics.
Lewis 1956 [78]	Inappropriate Patient Population, patients all over 1 year old.
Maataoui 2020 [79]	Inappropriate Patient Population. Patients all over 6 months of age.
Macdougall 1957 [80]	Inappropriate Patient Population. Average age is 2 years.
MacLean 1980 [81]	Inappropriate Comparator. Antibiotic usage compared to enteral feeding.
Maitland 2006 [82]	Inappropriate Patient Population. Median age of 25 months.
Martischnig 1952 [83]	Inappropriate Intervention and lack of full text.
Mathew 2016 [84]	Wrong Patient Population. Patient all aged over 6months.
Mathew 2016 [85]	Wrong Patient Population. Patient all aged over 6months.
Melaku 1999 [86]	Inappropriate Patient Population. Patient median age 5.9 years.
Moschovis 2014 [87]	Inappropriate Study Design. Patients treated for pneumonia.
Mtango 1986 [88]	Inappropriate Intervention. Patients given antibiotics to treat acute respiratory infections.
Muhammad 2020 [89]	Inappropriate Comparator. Compares nutritional treatment with antibiotics usage
Mulholland 1995 [90]	Inappropriate Patient Population. All patients have pneumonia to treat.
Muller 1953 [91]	Inappropriate Study Design and lack of full text.
Murce 1955 [92]	Lack of Full Text.
Muwanguzi 2021 [93]	Inappropriate Patient Population. Children aged 1 year and above.
Nalwanga 2020 [94]	Inappropriate Patient Population. Mean age 14 months.
Nalwanga 2020 [95]	Inappropriate Patient Population. Mean age 14 months.
Nansumba 2018 [96]	Inappropriate Study Design. observational study
Nantanda 2008 [97]	Inappropriate patient population. paediatric patients with pneumonia
NCT 2009 [98]	Inappropriate patient population. paediatric patients with pneumonia
NCT 2009 [99]	Inappropriate Patient Population. children age 6 months to 5 years
NCT 2020 [100]	Inappropriate outcome. studied the effect of prophylactic azithromycin has on the microbiome
NCT 2015 [101]	Inappropriate patient population . hospitalized patients (not necessarily patients with diarrheal illness) receiving prophylactic course of azithromycin (not antibiotics to treat diarrheal illness) at the time of discharge, outcomes of malnourished patients not published
NCT 2012 [102]	Inappropriate patient population . age 6 months to 5 years
Nuzhat 2005 [103]	Inappropriate study design . observational study

For peer review only

Antibiotic as an adjunct to treatment of severe acute malnutrition in infants less than six months of age. A protocol for the systematic review

List of Exclusions and Reasons for Exclusions.

	Inappropriate population . children with a skin disease
O'Brien 2021 [104]	Inappropriate Patient Population . children age 6 to 59 months
O'Brien 2020 [105]	Inappropriate intervention . studied the effect on biannual prophylactic azithromycin (not antibiotics to treat a diarrheal illness) on the mortality rate of malnourished children
O'Brien 2020 [106]	Inappropriate study design . observational study<br>Inappropriate intervention . biannual prophylactic azithromycin (not antibiotics for diarrheal illness)
Page 2013 [107]	Inappropriate Patient Population . children age 6 to 59 months
Pai 1985 [108]	Inappropriate Study Design
Parpia 2020 [109]	Inappropriate Study Design . antibiotics were given prophylactically to the children in the study (not in the treatment of diarrheal illness)<br>Inappropriate patient population: children did not start receiving the prophylactic antibiotics until after 6 months of age
Pinto 2012 [110]	Inappropriate Setting . PICU<br>Inappropriate study design . observational study
Polster 1954 [111]	Lack of Full Text
Pombo 2017 [112]	Inappropriate patient population . patients were hospitalized with pneumonia, not diarrheal illness<br>Inappropriate study design . observational study
Prentice 2013 [113]	Inappropriate Study Design . this paper is a review of various studies on the management of malnutrition in children
Rasul 2006 [114]	Inappropriate Study Design . randomized control trial but no placebo control, controls were admitted to the hospital prior to the study and thus did not receive the WHO protocol<br>Inappropriate patient population . malnourished children age 1 month to 5 years, included hospitalizations for many reasons not just diarrheal illness<br>Inappropriate intervention . compared WHO protocol vs no WHO protocol, so patients in both groups may have received antibiotics and the difference between the groups includes many interventions other than antibiotics
Rawson 2016 [115]	Inappropriate Study Design . letter to the editor
Reed 1996 [116]	Inappropriate Intervention . desired outcome was the difference in incidence of bacteremia and the difference in mortality rate among bacteremic patients between malnourished and adequately nourished children<br>Inappropriate study design . observational study<br>Inappropriate patient population . age 0 to 5 years
Roboz 1955 [117]	Lack of Full Text
Rogawski 2015 [118]	Inappropriate Study design . observational study

For peer review only

Antibiotic as an adjunct to treatment of severe acute malnutrition in infants less than six months of age. A protocol for the systematic review

List of Exclusions and Reasons for Exclusions.

Rokstad 2013 [119]	Inappropriate Patient Population . this paper is a brief review of a study done by Trehan in 2013, which studied a population of children age 6 to 59 months
Rosenberg 1974 [120]	Inappropriate Study Design . review/opinion paper
Roy 2010 [121]	Inappropriate Patient Population . children age 5 to 12 years<br>Inappropriate indication . desired outcome as whether there was a difference in the pharmacokinetics of isoniazid in malnourished children vs adequately nourished children
Sala 1955 [122]	Inappropriate Intervention and lack of full text
Samotra 1985 [123]	Inappropriate Indication . desired outcome was whether or not there was a difference in the pharmacokinetics of gentamicin in malnourished children vs adequately nourished children
Samotra 1986 [124]	Inappropriate Indication . desired outcome was whether there was a difference in the pharmacokinetics of chloramphenicol in malnourished children vs adequately nourished children
Sanogo 2018 [125]	Inappropriate Indication . desired outcome was the level of adherence among healthcare providers to WHO guidelines regarding management (antibiotics, oral rehydration therapy, and zinc) of paediatric diarrheal illness in Bamako, Mali.
Santoro 2002 [126]	Inappropriate Study Design . observational study.<br>Inappropriate indication . desired outcome was to see how many children admitted to the PICU for lower respiratory tract infections needed ICU level care
Schapira 1971 [127]	Lack of Full Text
Sepehr 2009 [128]	Inappropriate Patient population . age 2 to 94 years<br>Inappropriate study design . observational study<br>Inappropriate indication . desired outcome was optimal length of post-operative antibiotic prophylaxis as well as risk factors associated with post-operative infection
Shahira 2002 [129]	Inappropriate Patient Population . population was all pediatric patients at a teaching hospital<br>Inappropriate study design . observational study<br>Inappropriate indication . desired outcome was risk factors for nosocomial infections
Soheir 1981 [130]	Inappropriate Indication . desired outcome was the appropriate dose of chloramphenicol for malnourished children, not mortality
Standing 2018 [131]	Inappropriate indication . studied the proper dose of IV ceftriaxone and PO metronidazole needed to achieve a therapeutic level in malnourished children
Tan 2020 [132]	Inappropriate Indication . studied the use of anthropometric measurements as opposed to clinical signs to identify malnourished children
Taubenslag 1950 [133]	Inappropriate Study Design .
Thame 2001 [134]	Inappropriate Study Design . observational study

For peer review only

Antibiotic as an adjunct to treatment of severe acute malnutrition in infants less than six months of age. A protocol for the systematic review

List of Exclusions and Reasons for Exclusions.

Thorson 1989 [135]	Inappropriate Indication .
Tilg 2013 [136]	Inappropriate Study Design . this paper is a review of a few different studies.<br>Inappropriate patient population . Of interest, it discusses the results from Trehan 2013, which has a patient population of children age 6 months to 59 months
Tornberg-Belanger 2017 [137]	Inappropriate Study Design . observational study<br>Inappropriate topic . studied adherence to antibiotic guidelines of various hospitals
Trehan 2010 [138]	Inappropriate Patient Population . was children age 6 to 59 months<br>Inappropriate study design . observational study
Trehan 2013 [139]	Inappropriate Patient Population . children age 6 to 59 months
Trehan 2016 [140]	Inappropriate Study Design . same exact study as trehan 2013<br>Inappropriate patient population . children age 6 to 59 months
Trehan 2016 [141]	Inappropriate Patient Population . letter to editor
Usman 2019 [142]	Inappropriate Study Design . observational study
Uzan-Yulzari 2021 [143]	Wrong patient population. Age of patients range from birth to 2 years.
Vather 2018 [144]	Inappropriate Study Design . observational study
Verani 2019 [145]	Inappropriate Study Design . observational study.
Viso Gurovich 2003 [146]	Inappropriate study design . observational study.<br>Inappropriate Intervention . studied the outcomes of paediatric patients who received antibiotics to treat various infections, most commonly bronchopneumonia, complicated pneumonia, and conjunctivitis.
Vygen 2013 [147]	Inappropriate Study Design . case series (descriptive study design) looking at the outcomes of malnourished infants who received care at a nutritional rehab center
Walsh 2018 [148]	Inappropriate Intervention . tested the effectiveness of a lactose free legume based nutritional feed to promote the resolution of diarrhea
Weingaertner 1961 [149]	Lack of Full Text
Wittmann 1967 [150]	Inappropriate Patient Population. age rage was 2 months to 2 years and not all were malnourished
Woodd-Walker 1972 [151]	Inappropriate Study Design, inappropriate patient population . age range was from 1 year to 4 years
Zecca 1951 [152]	Lack of Full Text and study is from 1951
BMJ 2013 [153]	Wrong study design. Research news article.
Langdon 2018 [154]	Wrong patient population. Patient ages ranged from 6 weeks to 60 weeks with no subgroup data available for under 6 months.
Journal of Agricultural and Food Chemistry 1954 [155]	Wrong study design. News report article.
Akush 1951 [156]	Wrong indication. Patients all treated for pneumonia.

For peer review only

Antibiotic as an adjunct to treatment of severe acute malnutrition in infants less than six months of age. A protocol for the systematic review

List of Exclusions and Reasons for Exclusions.

Alam 2020 [157]	Wrong study design. All patients treated for diarrhea.

For peer review only

Antibiotic as an adjunct to treatment of severe acute malnutrition in infants less than six months of age. A protocol for the systematic review

List of Exclusions and Reasons for Exclusions.

1. Abate BB, Tilahun BD, Kassie AM, Kassaw MW. Treatment outcome of Severe Acute Malnutrition and associated factors among under-five children in outpatient therapeutics unit in Gubalafto Wereda, North Wollo Zone, Ethiopia, 2019. PLoS One 2020;15(9):e0238231. DOI: 10.1371/journal.pone.0238231.
2. Adam A. [Antibacterial and symbiontic nutrition of infants; a contribution to the problem of dysbacteria]. Arztl Forsch 1954;8(4):I/168-74. (In und).
3. Adem F, Edessa D, Bayissa B, Mohammed Hassen M, Mohammed MA. Treatment Outcomes and Associated Factors in Hospitalised Children with Severe Acute Malnutrition: A Prospective Cohort Study. Pediatric Health Med Ther 2020;11:235-243. DOI: 10.2147/PHMT.S253396.
4. Ahmed T, Ali M, Ullah MM, et al. Mortality in severely malnourished children with diarrhoea and use of a standardised management protocol. The Lancet 1999;353(9168):1919-1922. DOI: https://doi.org/10.1016/S0140-6736(98)07499-6.
5. Ahmed T, Begum B, Badiuzzaman, Ali M, Fuchs G. Management of severe malnutrition and diarrhea. The Indian Journal of Pediatrics 2001;68(1):45-51. DOI: 10.1007/BF02728857.
6. AKBANI Y, BOLME P, LINDBLAD BS, RAHIMTOOLA RJ. CONTROL OF STREPTOMYCIN AND ISONIAZID IN MALNOURISHED CHILDREN TREATED FOR TUBERCULOSIS. Acta Paediatrica 1977;66(2):237-240. DOI: https://doi.org/10.1111/j.1651-2227.1977.tb07840.x.
7. Amorelli Gonzaga M, Alce M, Mesquita da Cunha MO, Da Sil va Peres PR. The efficacy of miocamycin in eutrophic and malnourished children. Jornal de Pediatria 1989;65(6):223-230.
8. Amza A, Kadri B, Nassirou B, et al. A cluster-randomized controlled trial evaluating the effects of mass azithromycin treatment on growth and nutrition in Niger. Am J Trop Med Hyg 2013;88(1):138-143. (In eng). DOI: 10.4269/ajtmh.2012.12-0284.
9. Amza A, Yu SN, Kadri B, et al. Does mass azithromycin distribution impact child growth and nutrition in Niger? A cluster-randomized trial. PLoS Negl Trop Dis 2014;8(9):e3128. (In eng). DOI: 10.1371/journal.pntd.0003128.
10. Angelakis E, Million M, Kankoe S, et al. Abnormal Weight Gain and Gut Microbiota Modifications Are Side Effects of Long-Term Doxycycline and Hydroxychloroquine Treatment. Antimicrobial Agents and Chemotherapy 2014;58(6):3342-3347. DOI: doi:10.1128/AAC.02437-14.
11. Ashorn P. Empirical antibiotic treatment for children suffering from dysentery, cholera, pneumonia, sepsis or severe acute malnutrition. Paediatrics and International Child Health 2018;38(sup1):S1-S2. DOI: 10.1080/20469047.2018.1443546.
12. Ashraf H, Alam N, Gyr N. 126 Randomized Controlled Trial of Day-Care and Hospitalized Management of Severe Pneumonia with Severe Acute Malnutrition in Children in Bangladesh. Archives of Disease in Childhood 2012;97(Suppl 2):A35-A35. DOI: 10.1136/archdischild-2012-302724.0126.
13. Autret E, Gendrel D, Breteau M. [Digestive absorption of gentamicin in malnourished children]. Presse Med 1989;18(15):776. (In fre).
14. Ayelign B, Abebe B, Shibeshi A, et al. Bacterial isolates and their antimicrobial susceptibility patterns among pediatric patients with urinary tract infections. Turk J Urol 2018;44(1):62-69. (In eng). DOI: 10.5152/tud.2017.33678.
15. Ayiyi B, Hazoume FA, Dan V. A review of purulent meningitis in children in Benin. Revue de Pediatrie 1987;23(4):156-160.
16. Aziz F, Patil P. Role of Prophylactic Vitamin K in Preventing Antibiotic Induced Hypoprothrombinemia. The Indian Journal of Pediatrics 2015;82(4):363-367. DOI: 10.1007/s12098-014-1584-3.
17. Berkley JA. Cotrimoxazole Prophylaxis in Severely Malnourished Children. Clinical Trials (US National Library of Medicine) 2009 (https://clinicaltrials.gov/ct2/show/NCT00934492).

Antibiotic as an adjunct to treatment of severe acute malnutrition in infants less than six months of age. A protocol for the systematic review

List of Exclusions and Reasons for Exclusions.

18. Berkley JA, Ngari M, Thitiri J, et al. Daily co-trimoxazole prophylaxis to prevent mortality in children with complicated severe acute malnutrition: a multicentre, double-blind, randomised placebo-controlled trial. Lancet Glob Health 2016;4(7):e464-73. DOI: 10.1016/S2214-109X(16)30096-1.
19. BERKOWITZ FE. Infections in children with severe protein-energy malnutrition. The Pediatric Infectious Disease Journal 1992;11(9):750-759. (https://journals.lww.com/pidj/Fulltext/1992/09000/Infections_in_children_with_severe_protein_energy.15.aspx).
20. Bhatnagar S, Bhan MK, Sazawal S, et al. Efficacy of Massive Dose Oral Gentamicin Therapy in Nonbloody Persistent Diarrhea with Associated Malnutrition. Journal of Pediatric Gastroenterology and Nutrition 1992;15(2):117-124. (https://journals.lww.com/jpgn/Fulltext/1992/08000/Efficacy_of_Massive_Dose_Oral_Gentamicin_Therapy.3.aspx).
21. Bhatnagar S, Bhan MK, Singh KD, Shrivastav R. Prognostic Factors in Hospitalized Children with Persistent Diarrhea: Implications for Diet Therapy. Journal of Pediatric Gastroenterology and Nutrition 1996;23(2):151-158. (https://journals.lww.com/jpgn/Fulltext/1996/08000/Prognostic_Factors_in_Hospitalized_Children_with.10.aspx).
22. Birindwa AM, Manegabe JT, Mindja A, Nordén R, Andersson R, Skovbjerg S. Decreased number of hospitalized children with severe acute lower respiratory infection after introduction of the pneumococcal conjugate vaccine in the Eastern Democratic Republic of the Congo. Pan Afr Med J 2020;37:211. (In eng). DOI: 10.11604/pamj.2020.37.211.22589.
23. Bleakly NT, Axelrod DM, Maldonado YA, Chisti MJ, Kache S. ABSTRACT 597: CLINICAL OUTCOMES AND MORTALITY IN MALNOURISHED CHILDREN BEFORE AND AFTER IMPLEMENTATION OF A PEDIATRIC SEPSIS PROTOCOL. Pediatric Critical Care Medicine 2014;15(4_suppl):135. DOI: 10.1097/01.pcc.0000449323.13704.d1.
24. Boesen I, SamsØE-Jensen T. [Intramuscular penicillin and oral dihydrostreptomycin therapy of acute dyspepsia in bottlefed infants under 7 months]. Nord Med 1953;50(29):1023-4. (In und).
25. Bolme P, Gebre-Ab T, Hadgu P, Meeuwisse G, Stintzing G. Absorption and elimination of penicillin in children with malnutrition. Ethiop Med J 1980;18(4):151-7. (In eng).
26. Bolme P, Eriksson M, Habte D, Paalzow L. Pharmacokinetics of streptomycin in Ethiopian children with tuberculosis and of different nutritional status. European Journal of Clinical Pharmacology 1988;33(6):647-649. DOI: 10.1007/BF00542504.
27. Bolme P, Eriksson M, Paalzow L, Stintzing G, Zerihun G, Woldemariam T. Malnutrition and Pharmacokinetics of Penicillin in Ethiopian Children. Pharmacology & Toxicology 1995;76(4):259-262. DOI: https://doi.org/10.1111/j.1600-0773.1995.tb00140.x.
28. Bravo ME, Arancibia A, Jarpa S, Carpentier PM, Jahn AN. Pharmacokinetics of gentamicin in malnourrished infants. European Journal of Clinical Pharmacology 1982;21(6):499-504. DOI: 10.1007/BF00542045.
29. Bravo IG. The pharmacokinetics of cotrimoxazole sulphonamide in malnourished (marasmic) infants. Pediatric Pharmacology 01-1984;4(3):167-176.
30. Bredow MT, Jackson AA. Community based, effective, low cost approach to the treatment of severe malnutrition in rural Jamaica. Archives of Disease in Childhood 1994;71(4):297-303. DOI: 10.1136/adc.71.4.297.
31. Brodwall K. Uncertain effect of antibiotics for malnourished children. Tidsskrift for Den Norske Laegeforening 2016;136(6):532.
32. Bruhn C. Reducing the inconceivable deficiency: Studies on the use of antibiotics in undernourished African children. Deutsche Apotheker Zeitung 2016;156(30):425-435.

For peer review only

Antibiotic as an adjunct to treatment of severe acute malnutrition in infants less than six months of age. A protocol for the systematic review

List of Exclusions and Reasons for Exclusions.

33. Brunozi NA, David FL. Antibioticoterapia em crianças indígenas e não indígenas desnutridas. ABCS health sci 44(2):85-91.

34. Buchanan N. Letter To The Editor. Acta Paediatrica 1977;66(5):663-663. DOI: https://doi.org/10.1111/j.1651-2227.1977.tb07966.x.

35. Buchanan N, Eyberg C. Intramuscular tobramycin administration in kwashiorkor. S Afr Med J 1978;53(8):273-4. (In eng).

36. Buchanan N, Davis M, Eyberg C. Gentamicin pharmacokinetics in kwashiorkor. British Journal of Clinical Pharmacology 1979;8(5):451-453. DOI: https://doi.org/10.1111/j.1365-2125.1979.tb01024.x.

37. Bunn JEG. Severe acute malnutrition and HIV in African children. Future HIV Therapy 2009;3(6):595-611.

38. Cakir FB, Aydogan G, Timur C, et al. Effects of malnutrition on oxidative burst functions and infection episodes in children with acute lymphoblastic leukemia. International Journal of Laboratory Hematology 2012;34(6):648-654. DOI: https://doi.org/10.1111/j.1751-553X.2012.01451.x.

39. Çaksen H, Cesur Y, Üner A, et al. Urinary tract infection and antibiotic susceptibility in malnourished children. International Urology and Nephrology 2000;32(2):245-247. DOI: 10.1023/A:1007104326689.

40. Caksen H, Arslan S, Cesur Y, Sar S, Celebi V, Kuru M. Urinary tract infection and antibiotic susceptibility in malnourished children. Ceylon Med J 2000;45(2):77-9. (In eng). DOI: 10.4038/cmj.v45i2.8007.

41. Cedrato A, Larguia A, Galan HM. Administration of cefapirin every 12 hours in children with pneumonia: response according to protein nutritional status. Folha Medica 1974;68(1):1-4.

42. Chisti MJ, Salam MA, Bardhan PK, et al. Treatment Failure and Mortality amongst Children with Severe Acute Malnutrition Presenting with Cough or Respiratory Difficulty and Radiological Pneumonia. PLOS ONE 2015;10(10):e0140327. DOI: 10.1371/journal.pone.0140327.

43. Church JA, Fitzgerald F, Walker AS, Gibb DM, Prendergast AJ. The expanding role of co-trimoxazole in developing countries. The Lancet Infectious Diseases 2015;15(3):327-339. DOI: 10.1016/S1473-3099(14)71011-4.

44. Cochlovius A, Daecke K, Loeschke A. [Effect of various antibiotics in infant nutrition disorders]. Dtsch Med Wochenschr 1953;78(37):1266-70. (In und). DOI: 10.1055/s-0028-1114923.

45. Dicko A, Ouedraogo JB, Zongo I, et al. A trial of seasonal malaria chemoprevention plus azithromycin in African children. The American Society of Tropical Medicine and Hygiene 2016;95(5):481. (In English). DOI: 10.4269/ajtmh.abstract2016.

46. Dieng H, Aboussaleh Y, Sbaibi R, Bengueddour R. Evaluation of the care of infants and children under 5 years with severe undernutrition at the CRENAS of Nouakchott, Mauritania. Nutritional Therapy & Metabolism 2014;32:140-145. DOI: 10.5301/NTM.2014.12648.

47. Dubray C, Ibrahim SA, Abdelmutalib M, et al. Treatment of severe malnutrition with 2-day intramuscular ceftriaxone vs 5-day amoxicillin. Annals of Tropical Paediatrics 2008;28(1):13-22. DOI: 10.1179/146532808X270635.

48. Duffau G, Alvarez P, Chala E, Cruz C. [Manifestations of paralytic ileus in infants hospitalized for acute diarrheal syndrome]. Rev Chil Pediatr 1990;61(6):322-6. (In spa).

49. Dutta S, Chowdhary G, Kumar P, Mukhopadhay K, Narang A. Ciprofloxacin Administration to Very Low Birth Weight Babies has no Effect on Linear Growth in Infancy. Journal of Tropical Pediatrics 2005;52(2):103-106. DOI: 10.1093/tropej/fmi070.

For peer review only

Antibiotic as an adjunct to treatment of severe acute malnutrition in infants less than six months of age. A protocol for the systematic review

List of Exclusions and Reasons for Exclusions.

50. Eriksson M, Paalzow L, Bolme P, Mariam TW. Chloramphenicol pharmacokinetics in Ethiopian children of differing nutritional status. European Journal of Clinical Pharmacology 1983;24(6):819-823. DOI: 10.1007/BF00607094.
51. ERIKSSON M, BOLME P, HABTE D, PAALZOW L. INH and Streptomycin in Ethiopian Children with Tuberculosis and Different Nutritional Status. Acta Paediatrica 1988;77(6):890-894. DOI: https://doi.org/10.1111/j.1651-2227.1988.tb10774.x.
52. Ford GW, Belbin R, Jose DG, Vorbach EA, Kirke DK. Growth and Immune Function in Aboriginal Children during Recovery from Malnutrition and Infection. Australian and New Zealand Journal of Medicine 1976;6(4):321-328. DOI: https://doi.org/10.1111/imj.1976.6.4.321.
53. Friedrich MJ. Antibiotics of No Benefit in Treating Children With Malnutrition. JAMA 2016;315(11):1100-1100. DOI: 10.1001/jama.2016.2266.
54. Garcia de Olarte D, Trujillo H, Agudelo N, Nelson JD, Haltalin KC. Treatment of diarrhea in malnourished infants and children. A double-blind study comparing ampicillin and placebo. Am J Dis Child 1974;127(3):379-88. DOI: 10.1001/archpedi.1974.02110220077011.
55. Gardner R, Dwyer C, Morrison C. MINERVA. BMJ: British Medical Journal (Overseas & Retired Doctors Edition) 2011;343(7821):486.
56. Gennaro S, O'Connor C, Marx M. Global Health of Babies and Children. MCN: The American Journal of Maternal/Child Nursing 2017;42(3):132-138. DOI: 10.1097/nmc.0000000000000322.
57. Gensch F, Burmann R, Goertler H. Anstaltsdyspepsien und Kolisubstitution. Dtsch Med Wochenschr 1957;82(20):810-818. (In De).
58. Gernaat HBPE, Dechering WHJC, Voorhoeve HWA. Mortality in Severe Protein-energy Malnutrition at Nchelenge, Zambia. Journal of Tropical Pediatrics 1998;44(4):211-217. DOI: 10.1093/tropej/44.4.211.
59. Ghosh G, Chakraborty S, Ray J, Mukherjee SK. Parenteral ciprofloxacin in persistent diarrhoea in children. J Indian Med Assoc 1995;93(10):382, 384. (In eng).
60. Girma S, Sisay M, Mengistu G, Amare F, Edessa D. Antimicrobial Utilization Pattern in Pediatric Patients in Tertiary Care Hospital, Eastern Ethiopia: The Need for Antimicrobial Stewardship. Hosp Pharm 2018;53(1):44-54. DOI: 10.1177/0018578717737429.
61. Gore-Langton GR, Cairns M, Compaore YD, et al. Effect of adding azithromycin to the antimalarials used for seasonal malaria chemoprevention on the nutritional status of African children. Trop Med Int Health 2020;25(6):740-750. DOI: 10.1111/tmi.13390.
62. Hecht C, Weber M, Grote V, et al. Disease associated malnutrition correlates with length of hospital stay in children. Clin Nutr 2015;34(1):53-9. (In eng). DOI: 10.1016/j.clnu.2014.01.003.
63. Heikens GT, Schofield WN, Christie CD, Gernay J, Dawson S. The Kingston Project. III. The effects of high energy supplement and metronidazole on malnourished children rehabilitated in the community: morbidity and growth. Eur J Clin Nutr 1993;47(3):174-91. (In eng).
64. Heikens GT, Schofield WN, Dawson S. The Kingston Project. II. The effects of high energy supplement and metronidazole on malnourished children rehabilitated in the community: anthropometry. Eur J Clin Nutr 1993;47(3):160-73. (In eng).
65. Hirschhorn N. Can small daily doses of antibiotics prevent the cycle of diarrhea, malabsorption, and malnutrition in children? The American Journal of Clinical Nutrition 1971;24(7):872-875. DOI: 10.1093/ajcn/24.7.872.
66. Howard JE, Beca JP. CLINICAL ASSESSMENT OF HETACILLIN IN ACUTE DIARRHEA OF MALNOURISHED INFANTS. Annals of the New York Academy of Sciences 1967;145(2):436-441. DOI: https://doi.org/10.1111/j.1749-6632.1967.tb50243.x.

For peer review only

Antibiotic as an adjunct to treatment of severe acute malnutrition in infants less than six months of age. A protocol for the systematic review

List of Exclusions and Reasons for Exclusions.

67. Isaack H, Mbise RL, Hirji KF. Nosocomial bacterial infections among children with severe protein energy malnutrition. East Afr Med J 1992;69(8):433-6. (In eng).
68. Isanaka S, Grantz KH, Berthé F, Schaefer M, Adehossi E, Grais RF. Extended Follow-up From a Randomized Clinical Trial of Routine Amoxicillin in the Treatment of Uncomplicated Severe Acute Malnutrition in Niger. JAMA Pediatrics 2020;174(3):295-297. DOI: 10.1001/jamapediatrics.2019.5189.
69. Islam MR, Nuzhat S, Fahim SM, et al. Antibiotic exposure among young infants suffering from diarrhoea in Bangladesh. Journal of Paediatrics and Child Health 2021;57(3):395-402. DOI: https://doi.org/10.1111/jpc.15233.
70. ISRCTN. A study to compare antibiotics used to treat children with severe acute malnutrition. 2017 (https://trialsearch.who.int/?TrialID=ISRCTN18051843).
71. Kabalo MY, Seifu CN. Treatment outcomes of severe acute malnutrition in children treated within Outpatient Therapeutic Program (OTP) at Wolaita Zone, Southern Ethiopia: retrospective cross-sectional study. Journal of Health, Population and Nutrition 2017;36(1):7. DOI: 10.1186/s41043-017-0083-3.
72. Lares-Asseff I, Lugo-Goytia G, Pérez-Guillé MG, et al. [Bayesian prediction of chloramphenicol blood levels in children with sepsis and malnutrition]. Rev Invest Clin 1999;51(3):159-65. (In spa).
73. Lares-Asseff I, Pérz-Guillé MG, Camacho Vieyra GA, Pérez AG, Peregrina NB, Lugo Goytia G. Population Pharmacokinetics of Gentamicin in Mexican Children With Severe Malnutrition. The Pediatric Infectious Disease Journal 2016;35(8):872-878. DOI: 10.1097/inf.0000000000001204.
74. Lattes E, Piovano L. [Treatment of acute infant nutrition disorders and tests of sensitivity to antibiotics; clinical considerations]. Minerva Pediatr 1954;6(7):247-8. (In und).
75. Lebl J, Zahradníková M, Bartosová J, Zemková D, Pechová M, Vávrová V. Insulin-like growth factor-I and insulin-like growth factor-binding protein-3 in cystic fibrosis: a positive effect of antibiotic therapy and hyperalimentation. Acta Paediatr 2001;90(8):868-72. (In eng).
76. Lelijveld N, Godbout C, Krietemeyer D, et al. Treating high-risk moderate acute malnutrition using therapeutic food compared with nutrition counseling (Hi-MAM Study): a cluster-randomized controlled trial. The American Journal of Clinical Nutrition 2021;114(3):955-964. DOI: 10.1093/ajcn/nqab137.
77. Lepage P, Bogaerts J, Nsengumuremyi F, et al. Severe multiresistant Salmonella typhimurium systemic infections in Central Africa – clinical features and treatment in a paediatric department. Journal of Antimicrobial Chemotherapy 1984;14(suppl_B):153-159. DOI: 10.1093/jac/14.suppl_B.153.
78. Lewis RA, Bhagat MP, Wagle MM, Kulkarni BS, Satoskar RS. Antibiotic Dietary Supplements in the Therapy of Childhood Protein Mainutrition. The American Journal of Tropical Medicine and Hygiene 1956;5(3):483-496. (In English). DOI: 10.4269/ajtmh.1956.5.483.
79. Maataoui N, Langendorf C, Berthe F, et al. Increased risk of acquisition and transmission of ESBL-producing Enterobacteriaceae in malnourished children exposed to amoxicillin. Journal of Antimicrobial Chemotherapy 2019;75(3):709-717. DOI: 10.1093/jac/dkz487.
80. MACDOUGALL LG. THE EFFECT OF AUREOMYCIN ON UNDERNOURISHED AFRICAN CHILDREN. Journal of Tropical Pediatrics 1957;3(2):74-81. DOI: 10.1093/oxfordjournals.tropej.a057461.
81. MacLean WC, Jr., Lopez de Romaña G, Massa E, Graham GG. Nutritional management of chronic diarrhea and malnutrition: Primary reliance on oral feeding. The Journal of Pediatrics 1980;97(2):316-323. DOI: 10.1016/S0022-3476(80)80507-5.
82. Maitland K, Berkley JA, Shebbe M, Peshu N, English M, Newton CR. Children with severe malnutrition: can those at highest risk of death be identified with the WHO protocol? PLoS Med 2006;3(12):e500. (In eng). DOI: 10.1371/journal.pmed.0030500.

For peer review only

Antibiotic as an adjunct to treatment of severe acute malnutrition in infants less than six months of age. A protocol for the systematic review

List of Exclusions and Reasons for Exclusions.

83. Martischnig E. [Antibiotic therapy of severe nutritional disorders in infants]. Ann Paediatr 1952;179(3):168-87. (In und).
84. Mathew JL. Does Routine Antibiotic Therapy Benefit Children With Severe Acute Malnutrition?: Evidence-based Medicine Viewpoint. Indian Pediatr 2016;53(4):329-32. (In eng). DOI: 10.1007/s13312-016-0846-8.
85. Mathew JL, Rai R. Does routine antibiotic therapy benefit children with severe acute malnutrition? Indian Pediatrics 2016;53(4):329-333. DOI: 10.1007/s13312-016-0846-8.
86. Melaku A, Lulseged S. Chronic suppurative otitis media in a children's hospital in Addis Ababa, Ethiopia. Ethiop Med J 1999;37(4):237-46. (In eng).
87. Moschovis PP, Addo-Yobo EOD, Banajeh S, et al. Stunting is associated with poor outcomes in childhood pneumonia. Tropical medicine & international health : TM & IH 2015;20(10):1320-1328. (In eng). DOI: 10.1111/tmi.12557.
88. Mtango FDE, Neuvians D. Acute respiratory infections in children under five years. Control project in Bagamoyo District, Tanzania. Transactions of The Royal Society of Tropical Medicine and Hygiene 1986;80(6):851-858. DOI: 10.1016/0035-9203(86)90241-5.
89. Muhammad A SY, Nisar MI, Baloch B, Yazdani AT, Yazdani N, Jehan F. . Nutritional support for lactating women with or without azithromycin for infants compared to breastfeeding counseling alone in improving the 6-month growth outcomes among infants of peri-urban slums in Karachi, Pakistan-the protocol for a multiarm assessor-blinded randomized controlled trial Trials 2020;1(21):756.
90. MULHOLLAND EK, FALADE AG, CORRAH PT, et al. A randomized trial of chloramphenicol vs. trimethoprim-sulfamethoxazole for the treatment of malnourished children with community-acquired pneumonia. The Pediatric Infectious Disease Journal 1995;14(11):959-964. (https://journals.lww.com/pidj/Fulltext/1995/11000/A_randomized_trial_of_chloramphenicol_vs_.7.aspx).
91. Muller R. [Antibiotic therapy of nutrition disorders]. Kinderarztl Prax 1953:195-200. (In ger).
92. Murce A. [Current problems concerning etiology and therapy of acute nutritional disorders in early infancy; effect of chemotherapeutic agents and antibiotics]. Rev Bras Med 1955;12(5):306-13. (In por).
93. Muwanguzi E, Oboi JE, Nabbamba A, Wanyama R. Treatment outcome and associated factors for severely malnourished children (1–5 years) admitted to Lacor Hospital and Gulu Regional Referral Hospital in Uganda. Journal of Nutritional Science 2021;10:e33. DOI: 10.1017/jns.2021.11.
94. Nalwanga D, Musiime V, Kizito S, et al. Mortality among children under five years admitted for routine care of severe acute malnutrition: a prospective cohort study from Kampala, Uganda. BMC Pediatrics 2020;20(1):182. DOI: 10.1186/s12887-020-02094-w.
95. Nalwanga D, Musiime V, Kizito S, Kiggundu JB, Musoke P, Tumwine JK. Mortality among Children Hospitalized for Routine Care of Severe Acute Malnutrition. Pediatrics 2020;146(1 MeetingAbstract):299-299. DOI: 10.1542/peds.146.1_MeetingAbstract.299.
96. Nansumba M, Kumbakumba E, Orikiriza P, et al. Treatment outcomes and tolerability of the revised WHO anti-tuberculosis drug dosages for children. The International Journal of Tuberculosis and Lung Disease 2018;22(2):151-157. DOI: 10.5588/ijtld.17.0535.
97. Nantanda R, Hildenwall H, Peterson S, Kaddu-Mulindwa D, Kalyesubula I, K.Tumwine J. Bacterial aetiology and outcome in children with severe pneumonia in Uganda. Annals of Tropical Paediatrics 2008;28(4):253-260. DOI: 10.1179/146532808X375404.
98. International Centre for Diarrhoeal Disease Research B. Trial in Childhood Pneumonia With Malnutrition. 2009 (https://clinicaltrials.gov/ct2/show/NCT00968370).

For peer review only

Antibiotic as an adjunct to treatment of severe acute malnutrition in infants less than six months of age. A protocol for the systematic review

List of Exclusions and Reasons for Exclusions.

99. Medicine WUSo. Effectiveness of Oral Antibiotics in the Treatment of Severe Acute Malnutrition. 2009 (https://clinicaltrials.gov/ct2/show/NCT01000298).

100. University of California SF. Gut and Azithromycin Mechanisms in Infants and Children II (GAMINII). 2020 (https://clinicaltrials.gov/ct2/show/NCT04315272).

101. Washington Uo. Azithromycin to Prevent Post-discharge Morbidity and Mortality in Kenyan Children (Toto Bora). 2015 (https://clinicaltrials.gov/ct2/show/NCT02414399).

102. Epicentre. The Effect of Routine Antibiotic Use in the Outpatient Treatment of Severely Malnourished Children Without Complications. 2012 (https://clinicaltrials.gov/ct2/show/NCT01613547).

103. Yasmeen N, Khan MR. Spectrum of common childhood skin diseases: a single centre experience. J Pak Med Assoc 2005;55(2):60-3. (In eng).

104. O'Brien KS, Sié A, Dah C, et al. Azithromycin for uncomplicated severe acute malnutrition: study protocol for a pilot randomized controlled trial. Pilot and Feasibility Studies 2021;7(1):97. DOI: 10.1186/s40814-021-00836-w.

105. O'Brien KS, Arzika AM, Maliki R, et al. Biannual azithromycin distribution and child mortality among malnourished children: A subgroup analysis of the MORDOR cluster-randomized trial in Niger. PLoS Med 2020;17(9):e1003285. DOI: 10.1371/journal.pmed.1003285.

106. Kieran S. O'Brien AMA, Ramatou Maliki,Farouk Manzo,Alio K. Mamkara,Elodie Lebas,Catherine Cook,Robin L. Bailey,Sheila K. West,Catherine E. Oldenburg,Travis C. Porco,Benjamin Arnold,Jeremy D. Keenan,Thomas M. Lietman. The role of nutritional status in the efficacy of azithromycin to reduce child mortality in Niger: A subgroup analysis of the mordor trial. American Journal of Tropical Medicine and Hygiene 2019. DOI: 10.4269/ajtmh.abstract2019.

107. Page A-L, de Rekeneire N, Sayadi S, et al. Infections in Children Admitted with Complicated Severe Acute Malnutrition in Niger. PLOS ONE 2013;8(7):e68699. DOI: 10.1371/journal.pone.0068699.

108. Pai PM, Desai M, Vachharajani A. Drug therapy in malnourished children. 1985;25:25-28.

109. Parpia TC, Elwood SE, Scharf RJ, et al. Baseline Characteristics of Study Participants in the Early Life Interventions for Childhood Growth and Development in Tanzania (ELICIT) Trial. The American Journal of Tropical Medicine and Hygiene 2020;103(4):1397-1404. (In English). DOI: 10.4269/ajtmh.19-0918.

110. Pinto V. American Academy of Pediatrics Section on Critical Care Scientific Program Scientific Abstracts and Posters AAP National Conference and Exhibition October 21, 2012 – New Orleans, LA. Pediatric Critical Care Medicine 2012;13(6):704-710. DOI: 10.1097/PCC.0b013e31826df088.

111. Polster H. [Chloramphenicol treatment of acute nutrition disorders]. Kinderarztl Prax 1954;22(3):109-13. (In und).

112. Pombo-March P. Clinical profile of children and adolescents with and without comorbidities hospitalized with community acquired pneumonia. Pediatric Pulmonology 2017;52(S46):S100-S176. DOI: https://doi.org/10.1002/ppul.23731.

113. Prentice AM, Nabwera H, Kwambana B, Antonio M, Moore SE. Microbes and the Malnourished Child. Science Translational Medicine 2013;5(180):180fs11-180fs11. DOI: doi:10.1126/scitranslmed.3006212.

114. Rasul C, Aa M, Hasanuzzaman. Outcome of management of severe malnutrition in a medical institution. Paediatrics, Child and Adolescent Health Journal 2006;Paediatrics, Child and Adolescent Health Journal (Singapore) 2006; 46: 156-60.

115. Grais TMRLMAHITKSMMSIEAR. Amoxicillin for Severe Acute Malnutrition in Children. New England Journal of Medicine 2016;375:190-192. (https://www.nejm.org/doi/pdf/10.1056%2FNEJMc1605388).

For peer review only

Antibiotic as an adjunct to treatment of severe acute malnutrition in infants less than six months of age. A protocol for the systematic review

List of Exclusions and Reasons for Exclusions.

116. Reed RP, Wegerhoff FO, Rothberg AD. Bacteraemia in malnourished rural African children. Annals of Tropical Paediatrics 1996;16(1):61-68. DOI: 10.1080/02724936.1996.11747805.
117. Roboz P, Kadar M. [Increase of weight in infant dystrophy following isoniazid therapy]. Orv Hetil 1955;96(9):234-5. (In hun).
118. Rogawski ET, Westreich DJ, Adair LS, et al. Early Life Antibiotic Exposure Is Not Associated with Growth in Young Children of Vellore, India. J Pediatr 2015;167(5):1096-102.e3. (In eng). DOI: 10.1016/j.jpeds.2015.08.015.
119. Rokstad IS. Antibiotics in severe malnutrition in children. 2013. DOI: 10.4045/tidsskr.13.0366.
120. Rosenberg IH, Beisel WR, Gordon JE, et al. Infant and child enteritis-malabsorption-malnutrition: the potential of limited studies with low-dose antibiotic feeding. The American Journal of Clinical Nutrition 1974;27(3):304-309. DOI: 10.1093/ajcn/27.3.304.
121. Roy V, Gupta D, Gupta P, Sethi GR, Mishra TK. Pharmacokinetics of isoniazid in moderately malnourished children with tuberculosis. Int J Tuberc Lung Dis 2010;14(3):374-6. (In eng).
122. Sala F. [Observations on the use of combined chloramphenicol-sulfonamide therapy in acute nutritional disorders in pediatrics]. Gazz Med Ital 1955;114(3):54-8. (In ita).
123. Samotra K, Gupte S, Raina RK. Pharmacokinetics of gentamicin in protein-energy malnutrition. European Journal of Clinical Pharmacology 1985;29(2):255-256. DOI: 10.1007/BF00547433.
124. Samotra K, Gupte S, Raina RK. Effect of malnutrition on chloramphenicol kinetics in Indian children. Zhongguo Yao Li Xue Bao 1986;7(2):162-4. (In eng).
125. Sanogo D, Keita A, Roose A, et al. MANAGEMENT OF DIARRHEA IN MALI: ASSESSMENT OF THE ADHERENCE TO WHO RECOMMENDATIONS IN THE VACCINE IMPACT ON DIARRHEA IN AFRICA (VIDA) STUDY. AMERICAN JOURNAL OF TROPICAL MEDICINE AND HYGIENE: AMER SOC TROP MED & HYGIENE 8000 WESTPARK DR, STE 130, MCLEAN, VA 22101 USA; 2018:591-591.
126. SANTORO A, FERREIRA E, FERRARI AM. Infecciones respiratorias agudas bajas en niños menores de tres años: Referencia a unidades de cuidado intensivo. Archivos de Pediatría del Uruguay 2002;73:196-202. (http://www.scielo.edu.uy/scielo.php?script=sci_arttext&pid=S1688-12492002000400003&nrm=iso).
127. Schapira Y, Richtie J. [Combined therapy of Pseudomonas infection and severe malnutrition]. Harefuah 1971;80(11):608-10. (In heb).
128. Sepehr A, Santos B-JG, Chou C, et al. Antibiotics in head and neck surgery in the setting of malnutrition, tracheotomy, and diabetes. The Laryngoscope 2009;119(3):549-553. DOI: https://doi.org/10.1002/lary.20078.
129. Shahira S. BHAR, El Metwaly; Shoikar, Abdel Salam. Bacterial nosocomial infections among children admitted to wards at El-Shatby university children's hospital. Alexandria Journal of Pediatrics 2002;16:297-304.
130. Soheir M. DH, Shatla; Mona, Salem; Laila, El Sayed; Tahani E.S., Kholeif. Pharmacokinetics of cefazolin and chloramphenicol in protein-energy malnuirition. Journal of the Egyptian Society of Pharmacology and Experimental Therapeutics 1981;2:91-100.
131. Standing JF, Ongas MO, Ogwang C, et al. Dosing of Ceftriaxone and Metronidazole for Children With Severe Acute Malnutrition. Clinical Pharmacology & Therapeutics 2018;104(6):1165-1174. DOI: https://doi.org/10.1002/cpt.1078.
132. Tan R, Kagoro F, Levine GA, et al. Clinical Outcome of Febrile Tanzanian Children with Severe Malnutrition Using Anthropometry in Comparison to Clinical Signs. The American Journal of Tropical Medicine and Hygiene 2020;102(2):427-435. (In English). DOI: 10.4269/ajtmh.19-0553.

For peer review only

Antibiotic as an adjunct to treatment of severe acute malnutrition in infants less than six months of age. A protocol for the systematic review

List of Exclusions and Reasons for Exclusions.

133. Taubenslag L. [Streptomycin in acute nutritional disorders of infants in first half-year]. Dia Med 1950;22(48):1965-9. (In und).

134. Thame M, Stephen C, Wilks R, Forrester TE. The appropriateness of the current antibiotic empiric therapy based on the bacteria isolated from severely malnourished Jamaican children. West Indian Med J 2001;50(2):140-3. (In eng).

135. Thorson SM. Multiple-drug resistant Salmonella enteritis in malnourished children: Dramatic response to cefotaxime. 1989;11:279-287.

136. Tilg H, Moschen AR. Malnutrition and microbiota—a new relationship? Nature Reviews Gastroenterology & Hepatology 2013;10(5):261-262. DOI: 10.1038/nrgastro.2013.48.

137. Tornberg-Belanger S, Tickell K, Mangale D, et al. Pediatric inpatient antibiotic prescription practices in the chain network hospitals at baseline. American Society of Tropical Medicine and Hygiene; 2017.

138. Trehan I, Amthor RE, Maleta K, Manary MJ. Evaluation of the routine use of amoxicillin as part of the home-based treatment of severe acute malnutrition. Tropical Medicine & International Health 2010;15(9):1022-1028. (https://doi.org/10.1111/j.1365-3156.2010.02580.x). DOI: https://doi.org/10.1111/j.1365-3156.2010.02580.x.

139. Trehan I, Goldbach HS, LaGrone LN, et al. Antibiotics as Part of the Management of Severe Acute Malnutrition. New England Journal of Medicine 2013;368(5):425-435. DOI: 10.1056/NEJMoa1202851.

140. Trehan I, Goldbach HS, LaGrone LN, et al. Antibiotics as part of the management of severe acute malnutrition. Malawi Med J 2016;28(3):123-130. (In eng).

141. Trehan I. Amoxicillin for Severe Acute Malnutrition in Children. New England Journal of Medicine 2016;375(2):190-192. DOI: 10.1056/NEJMc1605388.

142. Farina Usman SS, Muhammad Amir, Muhammad Nadeem Chohan, Muhammad Touseef. Efficacy of Ampicillin and Gentamycin Combination Therapy in Children Having Severe Acute Malnutrition Presenting with Acute Diarrhea at Civil Hospital Hyderabad. Medical Forum Monthly 2019.

143. Atara Uzan-Yulzari OT, Anna Belogolovski, Oren Ziv, Christina Kunz, Sarah Perschbacher, Hadar Neuman, Edoardo Pasolli, Aia Oz, Hila Ben-Amram, Himanshu Kumar, Helena Ollila, Anne Kaljonen, Erika Isolauri, Seppo Salminen, Hanna Lagström, Nicola Segata, Itai Sharon, Yoram Louzoun, Regina Ensenauer,. Neonatal antibiotic exposure impairs child growth during the first six years of life by perturbing intestinal microbial colonization. Nature Communications 2021. DOI: 10.1038/s41467-020-20495-4.

144. Vather A, McKerrow NH, Morgan ME. Factors underlying the changing of antibiotics in a paediatric intensive care unit in Pietermaritzburg, KwaZulu-Natal2018.

145. Verani. Abstract Book. The American Journal of Tropical Medicine and Hygiene 2019;101(5_Suppl):1-668. (In English). DOI: 10.4269/ajtmh.abstract2019.

146. Gurovich F, Gómez-Oliván L, Velasco JM. Assessment of the use of chloramphenicol at the Neonatal Unit of a Mexican Paediatric Hospital. Pharmaceutical Care Espana 2003;5:182-185.

147. Vygen SB, Roberfroid D, Captier V, Kolsteren P. Treatment of Severe Acute Malnutrition in Infants Aged <6 Months in Niger. The Journal of Pediatrics 2013;162(3):515-521.e3. DOI: 10.1016/j.jpeds.2012.09.008.

148. Walsh K, Calder N, Olupot-Olupot P, et al. Modifying Intestinal Integrity and MicroBiome in Severe Malnutrition with Legume-Based Feeds (MIMBLE 2.0): protocol for a phase II refined feed and intervention trial [version 1; peer review: 2 approved]. Wellcome Open Research 2018;3(95). DOI: 10.12688/wellcomeopenres.14706.1.

149. Weingaertner L. [A new antibiotic, colistin, in the treatment of infant nutrition disorders]. Munch Med Wochenschr 1961;103:1628-31. (In ger).

For peer review only - http://bmjopen.bmj.com/site/about/guidelines.xhtml

Antibiotic as an adjunct to treatment of severe acute malnutrition in infants less than six months of age. A protocol for the systematic review

List of Exclusions and Reasons for Exclusions.

150. Wittmann W, Moodie AD, Hansen JD. A comparison of two antibiotic regimens in the treatment of gastroenteritis in malnourished children with a one-year follow-up of their subsequent progress. S Afr Med J 1967;41(16):414-7. (In eng).
151. WOODD-WALKER RB, HANSEN JDL, SAUNDERS SJ. THE IN VITRO UPTAKE OF LYSINE AND ALANINE BY HUMAN JEJUNAL MUCOSA IN PROTEIN-CALORIE MALNUTRITION, IN GASTROENTERITIS AND AFTER NEOMYCIN. Acta Paediatrica 1972;61(2):140-144. DOI: https://doi.org/10.1111/j.1651-2227.1972.tb15917.x.
152. Zecca E. [Streptomycin therapy in acute and chronic nutrition disorders in hospital]. Lattante 1951;22(9):536-40. (In und).
153. BMJ. Treating malnutrition with antibiotics. 2013:356. (https://regroup-production.s3.amazonaws.com/documents/ReviewReference/357822763/Treating%20malnutrition%20with%20antibiotics.pdf?AWSAccessKeyId=AKIAJBZQODCMKJA4H7DA&Expires=1643053445&Signature=SvDdx2aaEu%2B8i5CwDvlRAgoL52s%3D).
154. Amy Elizabeth Langdon XS, Sheila Isanaka, Gautam, Dantas. Prophylactic broad-spectrum antibiotics for childhood malnutrition. Journal of clinical and translational science 2018:46. (https://regroup-production.s3.amazonaws.com/documents/ReviewReference/357814158/Langdon%202018%20%20.pdf?AWSAccessKeyId=AKIAJBZQODCMKJA4H7DA&Expires=1643053619&Signature=UGJETRPj9mELVYWfjDUkVF%2Fhmqw%3D).
155. News: Antibiotic Supplements Overcome Growth Failure in School Children. Journal of Agricultural and Food Chemistry 1954;2(23):1148-1148. DOI: 10.1021/jf60043a605.
156. [Antibiotic albomycin in the treatment of pneumonias and toxemias in infants during the first year of life]. Feldsher Akush 1951;12:38. (In und).
157. Alam T, Ahmed D, Ahmed T, et al. A double-blind placebo-controlled trial of azithromycin to reduce mortality and improve growth in high-risk young children with non-bloody diarrhoea in low resource settings: the Antibiotics for Children with Diarrhoea (ABCD) trial protocol. Trials 2020;21(1):71. DOI: 10.1186/s13063-019-3829-y.

For peer review only

PRISMA 2020 Checklist

Section and Topic	Item #	Checklist item	Location where item is reported
**TITLE**			
Title	1	Identify the report as a systematic review.	p1
**ABSTRACT**			
Abstract	2	See the PRISMA 2020 for Abstracts checklist.	p3
**INTRODUCTION**			
Rationale	3	Describe the rationale for the review in the context of existing knowledge.	p4
Objectives	4	Provide an explicit statement of the objective(s) or question(s) the review addresses.	p4
**METHODS**			
Eligibility criteria	5	Specify the inclusion and exclusion criteria for the review and how studies were grouped for the syntheses.	p4
Information sources	6	Specify all databases, registers, websites, organisations, reference lists and other sources searched or consulted to identify studies. Specify the date when each source was last searched or consulted.	p5
Search strategy	7	Present the full search strategies for all databases, registers and websites, including any filters and limits used.	p5
Selection process	8	Specify the methods used to decide whether a study met the inclusion criteria of the review, including how many reviewers screened each record and each report retrieved, whether they worked independently, and if applicable, details of automation tools used in the process.	p6
Data collection process	9	Specify the methods used to collect data from reports, including how many reviewers collected data from each report, whether they worked independently, any processes for obtaining or confirming data from study investigators, and if applicable, details of automation tools used in the process.	p6
Data items	10a	List and define all outcomes for which data were sought. Specify whether all results that were compatible with each outcome domain in each study were sought (e.g. for all measures, time points, analyses), and if not, the methods used to decide which results to collect.	p5
	10b	List and define all other variables for which data were sought (e.g. participant and intervention characteristics, funding sources). Describe any assumptions made about any missing or unclear information.	p5
Study risk of bias assessment	11	Specify the methods used to assess risk of bias in the included studies, including details of the tool(s) used, how many reviewers assessed each study and whether they worked independently, and if applicable, details of automation tools used in the process.	p6
Effect measures	12	Specify for each outcome the effect measure(s) (e.g. risk ratio, mean difference) used in the synthesis or presentation of results.	p6
Synthesis methods	13a	Describe the processes used to decide which studies were eligible for each synthesis (e.g. tabulating the study intervention characteristics and comparing against the planned groups for each synthesis (item #5)).	P6
	13b	Describe any methods required to prepare the data for presentation or synthesis, such as handling of missing summary statistics, or data conversions.	P6
	13c	Describe any methods used to tabulate or visually display results of individual studies and syntheses.	P6
	13d	Describe any methods used to synthesize results and provide a rationale for the choice(s). If meta-analysis was performed, describe the model(s), method(s) to identify the presence and extent of statistical heterogeneity, and software package(s) used.	P6
	13e	Describe any methods used to explore possible causes of heterogeneity among study results (e.g. subgroup analysis, meta-regression).	p6
	13f	Describe any sensitivity analyses conducted to assess robustness of the synthesized results.	
Reporting bias assessment	14	Describe any methods used to assess risk of bias due to missing results in a synthesis (arising from reporting biases).	p6-7
Certainty assessment	15	Describe any methods used to assess certainty (or confidence) in the body of evidence for an outcome.	P7

For peer review only - http://bmjopen.bmj.com/site/about/guidelines.xhtml

PRISMA 2020 Checklist

Section and Topic	Item #	Checklist item	Location where item is reported
**RESULTS**			
Study selection	16a	Describe the results of the search and selection process, from the number of records identified in the search to the number of studies included in the review, ideally using a flow diagram.	p7
	16b	Cite studies that might appear to meet the inclusion criteria, but which were excluded, and explain why they were excluded.	supplement
Study characteristics	17	Cite each included study and present its characteristics.	NA
Risk of bias in studies	18	Present assessments of risk of bias for each included study.	NA
Results of individual studies	19	For all outcomes, present, for each study: (a) summary statistics for each group (where appropriate) and (b) an effect estimate and its precision (e.g. confidence/credible interval), ideally using structured tables or plots.	NA
Results of syntheses	20a	For each synthesis, briefly summarise the characteristics and risk of bias among contributing studies.	NA
	20b	Present results of all statistical syntheses conducted. If meta-analysis was done, present for each the summary estimate and its precision (e.g. confidence/credible interval) and measures of statistical heterogeneity. If comparing groups, describe the direction of the effect.	NA
	20c	Present results of all investigations of possible causes of heterogeneity among study results.	NA
	20d	Present results of all sensitivity analyses conducted to assess the robustness of the synthesized results.	NA
Reporting biases	21	Present assessments of risk of bias due to missing results (arising from reporting biases) for each synthesis assessed.	NA
Certainty of evidence	22	Present assessments of certainty (or confidence) in the body of evidence for each outcome assessed.	NA
**DISCUSSION**			
Discussion	23a	Provide a general interpretation of the results in the context of other evidence.	p7
	23b	Discuss any limitations of the evidence included in the review.	p7
	23c	Discuss any limitations of the review processes used.	p7
	23d	Discuss implications of the results for practice, policy, and future research.	p7
**OTHER INFORMATION**			
Registration and protocol	24a	Provide registration information for the review, including register name and registration number, or state that the review was not registered.	p4
	24b	Indicate where the review protocol can be accessed, or state that a protocol was not prepared.	p4
	24c	Describe and explain any amendments to information provided at registration or in the protocol.	p4
Support	25	Describe sources of financial or non-financial support for the review, and the role of the funders or sponsors in the review.	p9
Competing interests	26	Declare any competing interests of review authors.	p9
Availability of data, code and other materials	27	Report which of the following are publicly available and where they can be found: template data collection forms; data extracted from included studies; data used for all analyses; analytic code; any other materials used in the review.	p10-14

From: Page MJ, McKenzie JE, Bossuyt PM, Boutron I, Hoffmann TC, Mulrow CD, et al. The PRISMA 2020 statement: an updated guideline for reporting systematic reviews. BMJ 2021;372:n71. doi: 10.1136/bmj.n71

For more information, visit: http://www.prisma-statement.org/

For peer review only
